# The effect of flower position on variation and covariation in floral traits in a wild hermaphrodite plant

**DOI:** 10.1186/1471-2229-10-91

**Published:** 2010-05-20

**Authors:** Zhi-Gang Zhao, Guo-Zhen Du, Shuang-Quan Huang

**Affiliations:** 1 College of Life Sciences, Wuhan University, Wuhan 430072, China; 2 Key Laboratory of Arid and Grassland Ecology of Ministry of Education at Lanzhou University, Lanzhou 730000, China

## Abstract

**Background:**

Floral traits within plants can vary with flower position or flowering time. Within an inflorescence, sexual allocation of early produced basal flowers is often female-biased while later produced distal flowers are male-biased. Such temporal adjustment of floral resource has been considered one of the potential advantages of modularity (regarding a flower as a module) in hermaphrodites. However, flowers are under constraints of independent evolution of a given trait. To understand flower diversification within inflorescences, here we examine variation and covariation in floral traits within racemes at the individual and the maternal family level respectively in an alpine herb *Aconitum gymnandrum *(Ranunculaceae).

**Results:**

We found that floral traits varied significantly with flower position and among families, and position effects were family-specific. Most of the variance of floral traits was among individuals rather than among flowers within individuals or among families. Significant phenotypic correlations between traits were not affected by position, indicating trait integration under shared developmental regulation. In contrast, positive family-mean correlations in floral traits declined gradually from basal to distal flowers (nine significant correlations among floral traits in basal flowers and only three in distal flowers), showing position-specificity. Therefore, the pattern and magnitude of genetic correlations decreased with flower position.

**Conclusions:**

This finding on covariation pattern in floral reproductive structures within racemes has not been revealed before, providing insights into temporal variation and position effects in floral traits within plants and the potential advantages of modularity in hermaphrodites.

## Background

The flower as a reproductive organ can be regarded as a phenotypic module [1,2], in which floral traits are necessarily correlated with each other because the functional effectiveness of functionally linked floral parts (e.g. precise "fit" with pollinators, or optimal allocation to flower structures) depends on the ability to work together, and fitness depends on their interactions. Floral traits are thus expected to be more canalized and integrated than vegetative traits [3,4]. Studies based on comparisons between vegetative and floral characters support this expectation [5-11], although some conflicting evidence showed that this pattern may depend on plant species [9,11].

However, variation in floral traits occurs among populations, individuals, and flowers within one plant [12-18]. For example, Williams & Conner [17] studied sources of floral variation among different levels in wild radish (*Raphanus raphanistrum*) and found high intra-plant variance and high floral variation between flowers measured in different weeks. A common pattern observed in hermaphrodites is a reduction in the number or size of reproductive structures in sequentially blooming flowers [19,20]. This intra-inflorescence variation in floral allocation has been attributed to the effects of resource competition [21-23], architectural effects [20,24,25] or mating environments [26,27]. Although the ability to adjust sex allocation over time is one potential advantage of modularity in hermaphrodites [26,28,29], there are few empirical studies that examine temporal variation in floral traits [30,31], particularly considering trait correlations among sequentially blooming flowers.

Although strong correlation between floral traits may play an important role in the independent evolution of one floral trait [32], trait correlations frequently appear plastic [2,33,34], reflecting the environmental sensitivity. For example, genetic correlations among floral and vegetative traits in *Arabidopsis thaliana *were significantly influenced by the light environment [10]. Less attention has been paid to the possibility that intrinsic factors (i.e. ontogenetic or position effects) may be an important source of variation in trait correlations when there is great intra-individual variation of floral traits [31,35]. In an early multilevel analysis in *Dalechampia scandens*, Armbruster [12] showed that covariance between two of three measured reproductive traits was evenly distributed among three levels (within genets, among genets and among populations), although one of the traits was lack of ontogenetically-induced variation. A study on plasticity of floral traits of *Campanula rapunculoides *with respect to genotype, environment and ontogeny suggested that correlation patterns among floral traits depended on the environment and the trade-off relationship between male and female function was confounded by ontogenetic effects [36]. Considering ontogenetic variation in floral traits of *Iris gracilipes*, Ishii & Morinaga [18] found the pattern of correlations within individuals was basically similar to that among individuals. Comparing correlations among floral traits in closely related *Nicotiana *species, Bissell & Diggle [31] provided evidence of common developmental regulation of correlated traits but there were some independent trait variation with flower position and age. Although ontogenetic or positional effects have been considered in relation to covariation of floral traits, the importance of this aspect is still underappreciated for a full understanding of variation in floral traits. A more powerful approach involving calculation of genetic variance and covariance by measuring multiple traits in the offspring of full-sib or half-sib families has seldom been adopted in such studies. To our knowledge, only Mazer & Delesalle [14] have measured floral traits in half-sib families in *Spergularia marina *(Caryophyllaceae). They found that the statistical significance of correlations among six floral traits (number of ovules, normal anthers, abnormal anthers and petals, single petal area and total petal area) changed over sampling time.

In a previous study we observed variation in floral allocation in an alpine protandrous herb *Aconitum gymnandrum *(Ranunculaceae) with a racemose inflorescence, in which floral sex allocation shifted from female-biased to male-biased from basal to distal flowers [37]. To demonstrate the genetic basis of such temporal shift of floral traits that occurs in many plants, here we estimate broad sense heritability by a common garden experiment. We examine effects of flower position on floral traits and trait correlations in the herb, respectively at the individual level and at the maternal family level. Specifically, we (1) address variation of floral traits within racemes and among families, and examine whether position effects are family-specific; (2) explore correlations among floral traits at the individual and family levels, and their relationship to flower position, to examine whether correlations of floral traits vary within racemes.

## Results

### Variation of floral traits with flower position and among families

We examined the basal three, middle three and distal three flowers on each raceme in *Aconitum gymnandrum*. We measured seven traits in each raceme from 100 plants from 25 families that we cultivated in 100 pots, including height of the sepal galea, dry mass of floral structures (androecium, gynoecium and calyx), stamens and carpels per flower, and total flowers per raceme (see Additional file 1). Most of the floral traits varied significantly within racemes and/or among families. Effects of position and family on galea height (*F*_2, 99 _= 4.023, p = 0.011; *F*_24, 99 _= 2.812, *P *= 0.012, respectively), anther number (*F*_2, 99 _= 7. 654, *P *= 0.002; *F*_24, 99 _= 2.457, *P *= 0.021) and carpel number (*F*_2, 99 _= 86.715, *P *< 0.0001; *F*_24, 99 _= 2.703, *P *= 0.016) were significant (repeated measures ANOVA). For gynoecium mass, effect of position was significant (*F*_2, 99 _= 12. 349, *P *< 0.0001), while effect of family was not (*F*_24, 99 _= 1.714, *P *= 0.141). Significant position × family interactions were found in androecium mass (*F*_44, 99 _= 1.958, *P *= 0.022), anther number (*F*_44, 99 _= 2. 447, *P *= 0.003) and calyx mass (*F*_44, 99 _= 2.186, *P *= 0.006), showing that the intra-raceme variation was family-specific.

Significant among-family variation in galea height, anther number and carpel number was independent of flower position within racemes (Table 1). Among-family variations in androecium mass, gynoecium mass and calyx mass were not significant, and this finding is consistent across different positions.

**Table 1:** Summary of one-way ANOVAs to detect significant differences among maternal families with respect to floral traits at different positions within racemes.

		Flower position		
		
Traits	Stats.	Basal	Middle	Distal
Galea height (cm)	F_24, 99_	2.592	2.359	2.248
	Sig.	**0.018**	**0.029**	**0.037**
Androecium mass (g)	F_24, 99_	1.599	1.901	1.485
	Sig.	0.148	0.077	0.189
Anther number	F_24, 99_	2.415	2.41	2.679
	Sig.	**0.026**	**0.026**	**0.015**
Gynoecium mass (g)	F_24, 99_	1.315	2.091	1.354
	Sig.	0.271	0.051	0.249
Carpel number	F_24, 99_	2.183	2.814	2.639
	Sig.	**0.042**	**0.012**	**0.017**
Calyx mass (g)	F_24, 99_	0.698	0.764	1.18
	Sig.	0.794	0.731	0.357

Significant p-values appear in boldface type.

### Variance components of floral traits

**Figure 1:**
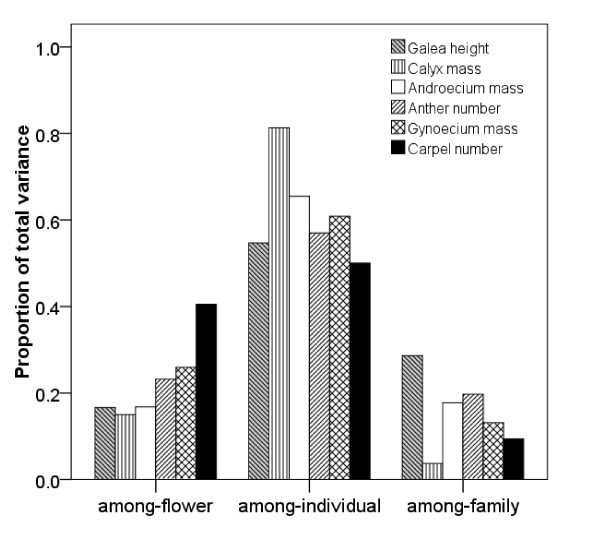
**Variance components from a nested ANOVA for floral traits at flower, individual and family levels, respectively**.

The largest variation in floral traits was among individuals (variance components from 0.50 to 0.81). Among-flower variation in these traits (variance components from 0.15 to 0.40) was higher than among-family variation (variance components from 0.04 to 0.29), except for galea height and androecium mass (Figure 1).

### Trait correlation and flower position

**Figure 2:**
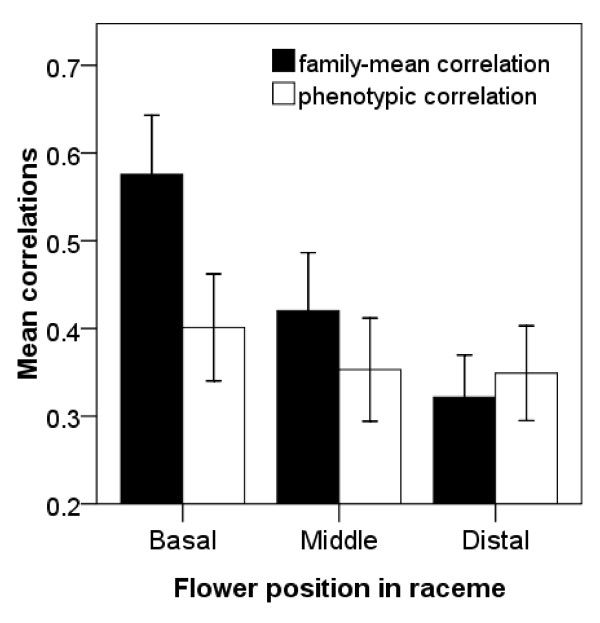
**Mean partial correlation coefficients among floral traits at different positions within racemes**. (Bars are mean ± SE. Results of Pearson correlation coefficients are consistent and not given.).

Partial correlation analysis indicated significant phenotypic correlations between most traits except for galea height (Table 2). The amounts of significant correlations were independent of flower positions, and phenotypic correlations did not vary within racemes. CPC analysis indicated that phenotypic correlations were similar across different positions with respect to equality, proportionality, and shared common principal components (*P *> 0.1 in all cases for the step-up and jump-up methods). The magnitude of the mean absolute correlation coefficients did not change significantly within racemes (Figure 2, basal vs. middle, *P *= 0.28, basal vs. distal, *P *= 0.253, bootstrapping estimation). Galea height was more closely correlated with gynoecium and calyx mass than it was with androecium mass.

**Table 2:** Partial correlations of floral traits among maternal family means at each of three different positions within racemes, controlling for variation in total flower number.

	Position	Galea Height (cm)	Androecium mass (g)	Anther number	Gynoecium mass (g)	Carpel number	Calyx mass (g)
Galea Height (cm)	Basal	1	0.221 (0.3)	-0.02 (0.926)	0.434 (0.034)	0.222 (0.297)	**0.519 **(0.009)
	Middle		-0.159 (0.457)	-0.188 (0.379)	0.409 (0.047)	0.208 (0.329)	0.055 (0.799)
	Distal		0.067 (0.755)	-0.242 (0.254)	0.172 (0.422)	0.006 (0.980)	-0.268 (0.206)
Androecium mass (g)	Basal	**0.149 (0.002)**	1	0.898***	0.722***	**0.518 **(0.01)	0.765***
	Middle	**0.112 **(0.009)		0.900***	**0.575 (0.003)**	**0.476 **(0.019)	**0.474 **(0.019)
	Distal	0.171***		**0.589 (0.002)**	0.360 (0.084)	0.231 (0.278)	**0.521 **(0.009)
Anther number	Basal	-0.03 (0.535)	0.77***	1	0.666***	**0.557 (0.005)**	**0.631 (0.001)**
	Middle	-0.031 (0.47)	0.742***		**0.595 (0.002)**	**0.579 (0.003)**	0.384 (0.064)
	Distal	0.032 (0.502)	0.70***		**0.573 (0.003)**	0.404 (0.050)	0.371 (0.075)
Gynoecium mass (g)	Basal	0.27***	0.41***	**0.145 (0.002)**	1	0.859***	0.876***
	Middle	0.253***	0.359***	0.181***		0.835***	0.401 (0.052)
	Distal	0.228***	0.361***	0.17***		**0.573 (0.003)**	0.295 (0.161)
Carpel number	Basal	**0.162 (0.001)**	0.424***	0.333***	0.738***	1	0.728***
	Middle	**0.113 **(0.008)	0.258***	0.254***	0.744***		0.066 (0.761)
	Distal	**0.12 **(0.012)	0.352***	0.28***	0.715***		0.152 (0.478)
Calyx mass (g)	Basal	0.3***	0.723***	0.396***	0.633***	0.535***	1
	Middle	0.327***	0.694***	0.374***	0.515***	0.339***	
	Distal	0.283***	0.644***	0.298***	0.504***	0.378***	

Genetic correlations among maternal family means occur above the diagonal, n = 25; phenotypic correlations at individual level occur below the diagonal, n = 100. Significant coefficients, following Bonferroni corrections on each correlation matrix using a table-wide *p *< 0.05, appear in boldface type. *** indicates *p *<**0.0001**.

**Figure 3:**
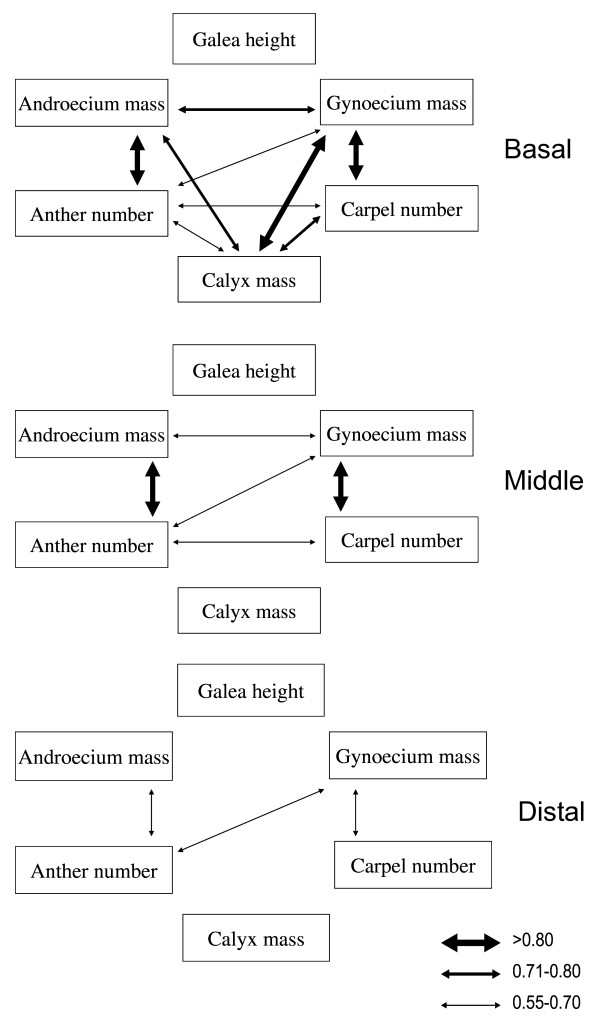
**Diagrams for family-mean correlations among floral traits at different positions within racemes**. (Arrow thickness depends on correlation coefficients taken from the results of partial correlation. Only significant coefficients after Bonferroni corrections were used).

The relative numbers of significant correlations among family means depended strongly on flower position within racemes (Table 2 and Figure 3): there were nine significant correlations among floral traits in basal flowers, but only three in distal flowers. Correlations between androecium mass, anther number, gynoecium mass, carpel number and calyx mass were positive in basal flowers, but these correlations with calyx mass were not significant in either middle or distal flowers (Figure 3). Correlations between androecium mass and anther number, gynoecium mass and carpel number occurred in both basal and middle flowers, but were lost in distal flowers. And mean absolute coefficients of family-mean correlation declined significantly from basal to distal positions within racemes (Figure 2, basal vs. middle, *P *= 0.046, basal vs. distal, *P *= 0.0012, bootstrapping estimation). CPC analysis showed that family-mean correlation matrices were not similar or not proportional across positions (*P *< 0.01, using either methods), but shared common principal components (*P *= 0.760 using the step-up method, *P *= 0.696 using the jump-up method).

Significantly positive correlations were observed between male (androecium mass, anther number) and female (gynoecium mass, carpel number) allocation (Figure 3 and Table 2). However, the significant correlations depended in part on flower position within racemes, e.g. anther number (but not androecium mass) was significantly correlated with gynoecium mass irrespective of flower position (Figure 3 and Table 2).

## Discussion

### Variation in floral traits

We demonstrated that most floral traits varied significantly within racemes in *A. gymnandrum*, indicating that traits were affected by flower position. Positional or temporal effects on floral traits were also found in *Dalechampia *[12,38], *Spergularia marina *[14,15], *Raphanus raphanistrum *[17] and in *Nicotiana *[31]. Temporal variation in floral trait expression could mask underlying genetic differences among individuals, families, populations or species in floral phenotype [15,35,39], resulting in sensitivity of heritability estimates to the position of measured flowers. In our studied herb, significant variation among families for most floral traits (except for gynoecium mass) was independent of flower position within racemes. The magnitude of differences among populations and among maternal family means in most floral traits of *S*. *marina*, however, was observed to change through the flowering period [15]. We observed significant position × family interactions in androecium mass, anther number and calyx mass, showing that the intra-raceme pattern in *A. gymnandrum *was family-specific. A genotype-specific intra-inflorescence pattern was observed for pollen and ovule number per flower in *Fragaria virginiana *(Rosaceae) as shown by significant genotype-by-flower position interactions [40]. In *S. marina*, no family-specific effects of sampling week on floral traits were detected except in the case of ovule number per flower which showed significant week × family interactions [15]. The family-differential intra-raceme patterns in *A. gymnandrum *suggest that genetic sources of variation in floral traits contribute significantly to floral phenotype.

It has been suggested that larger variation in floral traits within individuals than among individuals may cushion differential selection pressures on floral evolution [14,17]. For example, if pollinator-mediated selection acts as one force influencing floral variation, the evolutionary response to selection will be weakened if there is a wide range of floral variation within individuals rather than among individuals. But evidence supporting this expectation has been scarce [12,41]. We found that variation in floral traits was greater among plants than among flowers within individuals or among families in bumblebee-pollinated *Aconitum gymnandrum*, consistent with the expectation. In contrast to plants pollinated by specialists, studies on generalist-pollinated species showed intra-plant variation in floral traits to be larger than inter-plant variation [16,17]. For example, Williams & Conner [17] found that high within-plant variance of floral traits in the field accorded with the weak selection on floral traits in wild radish which was pollinated by diverse insects. In another Brassicaceae species *Brassica rapa*, however, the inter-plant variance of floral traits was similar to the intra-plant component [42]. Variation in floral traits was also similar between intra- and inter-plant levels in *Iris gracilipes *[18]. Given that few studies consider variation in floral traits both within and among individuals, at present it is impossible to assess whether the pattern of high inter-plant variation in floral traits is associated with the pollinator specialization level.

### Variation in trait correlation

We observed that significant phenotypic correlations between most traits (except for galea height) were stable and independent of flower position. Although these traits independently varied among positions as exhibited above, it is not great enough to decouple the strong correlation between traits. Similarly, a study on *Nicotiana *exhibited stability of correlation patterns of length characters rather than width characters of flowers independent of cyme position, flower position and flower age, showing a pattern of shared developmental regulation [31]. Variation in floral traits rather than trait correlations across environments has also been documented in other studies [41,43]. Constant correlation patterns prove that these floral traits are developmental integrated; on the other hand, if there is indirect or correlated selection on one of the floral traits in *A. gymnandrum*, it would not be strongly influenced by flower position within racemes.

In contrast, we observed that significant correlations among family means depended strongly on flower position in *A. gymnandrum*, and the correlation matrices varied among different positions. Floral traits of basal flowers were more significantly correlated than traits of distal flowers. Positive family-mean correlations between floral traits weakened gradually from basal to distal flowers, i.e. the strength of correlations were position-specific. This suggests that later flowers within racemes are subject to less genetic constraint or developmental stability than earlier produced distal flowers. At the same time, it also indicates a high potential for evolutionary modification because of the low genetic correlation among traits; variation observed among maternal sibships could reflect genetic differences to some extent [38,44,45]. To our knowledge, the position-dependent decline in the magnitude of family-mean correlations rather than phenotypic correlations has not been reported before. Our result is mirrored in *Spergularia marina *in which the phenotypic and among-family correlations among traits changed over the sampling period although the trend was inconsistent [14]. It has been suggested that genetic correlations are variable and could be affected by the developmental age of an individual or by environmental factors [46,47]. For example, genetic correlations of life history traits in *Geranium *changed from generally negative in the early juvenile stage to strongly positive in the adult [48]. Genetic correlations among floral and vegetative traits were significantly influenced by the light environment in *Arabidopsis thaliana *[10]. The variability of genetic correlation is critical for understanding trait evolution under natural selection.

The greater variation of floral traits in later flowers compared to early flowers of *A. gymnandrum *may be in part due to weakened trait correlations of family means (i.e. weakened genetic correlation). Strong genetic correlations would restrict independent evolution of floral traits [31,49], but intra-raceme variation in genetic correlations of floral traits would cushion selection on floral traits and trait integration within racemes, and consequently on the intra-raceme pattern of *A. gymnandrum*. For example, at an early developmental stage of the raceme, evolutionary changes of basal flowers may be constrained in terms of high genetic correlation; at a later stage, evolutionary changes of distal flowers may be less constrained due to low genetic correlation. It has been suggested that floral traits and their correlations could be regulated at the level of individual flowers [18,28], and that developmental plasticity and canalization are independently controlled [38]. Genetic factors and diverse environmental factors have been demonstrated to increase developmental plasticity in plants [41]. In andromonoecious species, basal flowers invariably developed into perfect flowers but distal flowers were plastic, capable of developing into either a staminate flower or a perfect flower [50]. Such temporal variation of later-produced flowers, with the potential to specialize as males [26], can be better understood if the intra-inflorescence pattern of trait correlations can be revealed, as in our demonstration in *A. gymnandrum *that later or distal flowers were more labile than early basal flowers. Thus, our findings provide insight into the plasticity of sexual expression and regulation of floral development in hermaphrodite plants.

## Conclusions

In conclusion, floral traits in *A. gymnandrum *varied significantly within racemes and among maternal families, and the position effect was family-specific. Variation of floral traits among individuals was greater than that among flowers within individuals or among families. Although significant phenotypic correlations in floral traits did not change among flower positions, the pattern of family-mean correlations varied and the magnitude declined gradually from basal to distal flowers, exhibiting position-specific correlations. This shows that flowers within an individual have dissimilar evolutionary potential. Thus, data from *A. gymnandrum *suggest that position effect on the magnitude of covariation in floral traits would confound the evolution of different flowers within individuals and consequently influence selection on intra-raceme pattern in flowering plants. Our findings on variation and covariation in floral traits with position provide genetic basis of flower functional specialization within individuals [20,26].

## Methods

### Plant material

*Aconitum gymnandrum *Maxim. (Ranunculaceae) is an annual herb, widely distributed in alpine meadows (1600-3800 m a.s.l) in the Qinghai-Tibet Plateau, China. Individual plants generally produce one erect raceme consisting of 2-30 blue-purple zygomorphic flowers, which open sequentially from bottom to top. Each flower has 6-14 separate carpels (each with 8-14 ovules) surrounded by 30-90 stamens. The galea (or hood), formed from one of five petaloid sepals, contains two stalked petals with nectaries. The species is self-compatible, strongly protandrous like other related species in the same genus [51] and bumblebee-pollinated. The anthers dehisce over 4-5 days and stigmas become receptive 1-2 days later. Plants commonly bloom from June through August and single flowers last 6-10 days. Fruit maturation requires 20-30 days.

### Experimental methods

In this experiment seedlings from different maternal plants were transplanted into pots to investigate among-family variation and covariation of floral traits. In September 2003, we collected seeds from 25 maternal plants of *A. gymnandrum *from populations of an alpine meadow in northeastern Qinghai-Tibet Plateau near Hezuo County, Gansu Province, China. Plants were at least 10 m apart to ensure genetic differences among individuals. Seeds were stored in envelopes at room temperature.

On 7 May 2004, seeds from the 25 maternal plants were germinated in Petri dishes with distilled water. Eight ten-day-old seedlings of uniform size were selected from each family, and seedlings were transplanted in pairs into assigned positions in each of four plastic pots (diameter 26 cm) filled with mixed soil collected from a local natural site where *A. gymnandrum *grows naturally. The soil had previously been mixed and covered with film for four months to eliminate preexisting seeds. These plants were used to measure floral traits when they flowered. Irrespective of families, all pots were randomly arranged at the field station of Lanzhou University at Hezuo County (E102°53', N34°55') and exposed to the natural environment. This design was to simulate natural growing conditions in the field.

In July 2005, all of the experimental plants bloomed, and natural mortality was less than 10%. For the pots of 25 families, one plant per pot was picked randomly, resulting in a sample of four for each family. We collected all sequentially blooming flowers within racemes of a total of 100 plants from 25 families, when the flowers had just opened. Racemes of all plants contained more than 9 flowers. We grouped flowers into three positions within racemes: the basal three, the middle three and the distal three on each raceme. For each flower we measured the height of the sepal galea (from the base of the sepal to the top of the galea, to the nearest 0.01 mm, with a vernier caliper), weighed the dry mass of floral structures (androecium, gynoecium and calyx, after drying at 80°C for 24h), and counted stamens and carpels per flower and the total flowers per raceme.

### Data analysis

Variation in the traits within racemes and variation of all traits among families were analyzed by repeated measures ANOVA (the GLM procedure of SPSS), with flower position as within-subject factor (three levels) and family as subject factor. We also analyzed variation of floral traits among families with one-way ANOVA (GLM model of SPSS for Windows) at different positions separately, with family as a random factor. To examine the relative magnitudes of sources of variation in floral traits, among flowers within individuals, among individuals and among families, variance components from fully nested random models were estimated using REML (mixed model of SPSS).

Family-mean correlations between traits were estimated separately at each position; each family's mean was calculated from the phenotypic means of the individuals representing it. Family-mean correlations reflect the genetic base to some extent and can be regarded as genetic correlation [38,44,45]. To remove the effect of difference in plant size, we controlled for differences among families in plant size by estimating partial correlation coefficients between traits, controlling for total flower number. We also estimated phenotypic correlations between traits across positions, removing the effect of difference in plant size using the same methods as we used for the among-family correlations. To determine if correlations (genetic and phenotypic) among floral traits vary with flower position, common principal component analysis (Flury hierarchical method) was used to compare these correlation matrices between positions by the step-up method and the jump-up method [52,53]. Differences between flower positions in the mean absolute coefficients of family-mean and phenotypic correlations were estimated by a bootstrapping method (n = 10 000 permutations, using Data Pilot ver.1.03, [54]), because the values used in calculating correlation coefficients were not independent. To reduce the probability of spurious results caused by the simultaneous evaluation of multiple statistical tests, sequential Bonferroni corrections [55] were used in each correlation matrix (family correlations at each of flower positions separately), with a table-wide significance value of p < 0.05. All analyses were completed in SPSS statistical software (Version 12.0 for Windows).

## Authors' contributions

ZGZ, GZD and SQH designed the research. ZGZ performed the research and analyzed the data. ZGZ drafted the manuscript. SQH contributed to writing the manuscript. All authors read and approved the final version.

## Supplementary Material

Additional file 1 **Data of floral traits at different positions within racemes of *Aconitum gymnandrum***. Seven traits for flowers from basal, middle and distal racemes were measured from 100 plants from 25 families. ID represents individuals; Position 1, 2 and 3 represents basal, middle, distal respectively. Click here for file
