# Supplementary material for: The effect of flower position on variation and covariation in floral traits in a wild hermaphrodite plant
Source: BMC Plant Biol. 2010 May 20;10:91. doi: 10.1186/1471-2229-10-91 (PMC3095358; doi:10.1186/1471-2229-10-91)
Supplement: Additional file 1 — Data of floral traits at different positions within racemes of Aconitum gymnandrum. Seven traits for flowers from basal, middle and distal racemes were measured from 100 plants from 25 families. ID represents individuals; Position 1, 2 and 3 represents basal, middle, distal respectively. [file 1471-2229-10-91-S1.PDF]

| Family | ID | Position | Galea height | Androecium mass | Anther number | Gynoecium mass | Carpel number | Calyx mass | Flower number |
|---|---|---|---|---|---|---|---|---|---|
| a | 4 | 1 | 1.62 | 0.00685 | 75 | 0.00678 | 20 | 0.01371 | 9 |
| a | 4 | 1 | 1.732 | 0.00786 | 75 | 0.00746 | 16 | 0.01719 | 9 |
| a | 4 | 1 | 1.768 | 0.01034 | 82 | 0.00133 | 8 | 0.01411 | 9 |
| a | 4 | 2 | 1.266 | 0.0038 | 83 | 0.00321 | 14 | 0.00928 | 9 |
| a | 4 | 2 | 1.23 | 0.00766 | 86 | 0.00257 | 11 | 0.01269 | 9 |
| a | 4 | 2 | 1.732 | 0.00908 | 90 | 0.00127 | 7 | 0.01286 | 9 |
| a | 4 | 3 | 1.642 | 0.00478 | 72 | 0.00423 | 16 | 0.01142 | 9 |
| a | 4 | 3 | 1.516 | 0.00897 | 85 | 0.00291 | 11 | 0.01414 | 9 |
| a | 4 | 3 | 1.388 | 0.00465 | 73 | 0.00394 | 12 | 0.0102 | 9 |
| a | 5 | 1 | 1.674 | 0.0092 | 60 | 0.00493 | 13 | 0.01618 | 19 |
| a | 5 | 1 | 1.52 | 0.00847 | 63 | 0.00372 | 12 | 0.01649 | 19 |
| a | 5 | 1 | 1.674 | 0.00873 | 62 | 0.00616 | 10 | 0.01322 | 19 |
| a | 5 | 1 | 1.564 | 0.00766 | 64 | 0.00364 | 9 | 0.01569 | 19 |
| a | 5 | 1 | 1.92 | 0.00754 | 61 | 0.00581 | 9 | 0.01733 | 19 |
| a | 5 | 1 | 1.688 | 0.00582 | 69 | 0.00772 | 9 | 0.01531 | 19 |
| a | 5 | 2 | 1.942 | 0.006 | 61 | 0.00828 | 13 | 0.01551 | 19 |
| a | 5 | 2 | 1.648 | 0.00836 | 66 | 0.00326 | 7 | 0.01434 | 19 |
| a | 5 | 2 | 1.904 | 0.00577 | 65 | 0.00522 | 13 | 0.01497 | 19 |
| a | 5 | 2 | 1.688 | 0.00609 | 62 | 0.00699 | 15 | 0.01771 | 19 |
| a | 5 | 2 | 1.71 | 0.00625 | 63 | 0.00859 | 17 | 0.01661 | 19 |
| a | 5 | 2 | 1.884 | 0.00526 | 58 | 0.00202 | 7 | 0.01079 | 19 |
| a | 5 | 2 | 1.868 | 0.00631 | 64 | 0.00186 | 6 | 0.01131 | 19 |
| a | 5 | 3 | 1.904 | 0.00619 | 63 | 0.00122 | 7 | 0.01142 | 19 |
| a | 5 | 3 | 1.878 | 0.0064 | 61 | 0.00123 | 6 | 0.01099 | 19 |
| a | 5 | 3 | 1.674 | 0.00606 | 67 | 0.00099 | 5 | 0.01144 | 19 |
| a | 5 | 3 | 1.636 | 0.00655 | 69 | 0.00083 | 5 | 0.01125 | 19 |
| a | 5 | 3 | 1.738 | 0.00663 | 68 | 0.00083 | 6 | 0.01162 | 19 |
| a | 5 | 3 | 1.89 | 0.00656 | 64 | 0.00175 | 6 | 0.01031 | 19 |
| a | 6 | 1 | 1.828 | 0.00867 | 65 | 0.00184 | 10 | 0.01554 | 21 |
| a | 6 | 1 | 1.832 | 0.00875 | 65 | 0.00384 | 8 | 0.01694 | 21 |
| a | 6 | 1 | 1.876 | 0.00926 | 70 | 0.00745 | 12 | 0.01679 | 21 |
| a | 6 | 1 | 1.98 | 0.01135 | 68 | 0.00563 | 11 | 0.01724 | 21 |
| a | 6 | 1 | 1.938 | 0.01035 | 68 | 0.0058 | 8 | 0.01695 | 21 |
| a | 6 | 1 | 1.914 | 0.00988 | 67 | 0.0106 | 13 | 0.01747 | 21 |
| a | 6 | 1 | 1.924 | 0.01059 | 63 | 0.0102 | 13 | 0.01776 | 21 |
| a | 6 | 2 | 1.918 | 0.00982 | 69 | 0.00516 | 9 | 0.01864 | 21 |
| a | 6 | 2 | 1.71 | 0.01029 | 66 | 0.0058 | 8 | 0.01776 | 21 |
| a | 6 | 2 | 1.932 | 0.01136 | 67 | 0.00514 | 8 | 0.0202 | 21 |
| a | 6 | 2 | 1.572 | 0.00964 | 61 | 0.00602 | 9 | 0.00858 | 21 |
| a | 6 | 2 | 1.688 | 0.00568 | 46 | 0.00385 | 8 | 0.01415 | 21 |
| a | 6 | 2 | 1.92 | 0.00878 | 57 | 0.0066 | 12 | 0.01598 | 21 |

| | | | | | | | | | |
|---|---|---|---|---|---|---|---|---|---|
| a | 6 | 2 | 1.74 | 0.00632 | 47 | 0.00409 | 8 | 0.01536 | 21 |
| a | 6 | 3 | 1.826 | 0.00728 | 48 | 0.00613 | 11 | 0.01782 | 21 |
| a | 6 | 3 | 1.802 | 0.00901 | 52 | 0.00564 | 10 | 0.01842 | 21 |
| a | 6 | 3 | 1.89 | 0.00938 | 55 | 0.00695 | 12 | 0.01688 | 21 |
| a | 6 | 3 | 1.962 | 0.00744 | 64 | 0.00765 | 11 | 0.01835 | 21 |
| a | 6 | 3 | 1.828 | 0.00796 | 69 | 0.00644 | 10 | 0.01748 | 21 |
| a | 6 | 3 | 1.702 | 0.01153 | 52 | 0.00481 | 6 | 0.01277 | 21 |
| a | 7 | 1 | 2.016 | 0.01153 | 64 | 0.00983 | 12 | 0.01939 | 25 |
| a | 7 | 1 | 1.854 | 0.01241 | 70 | 0.00856 | 12 | 0.01894 | 25 |
| a | 7 | 1 | 1.888 | 0.01229 | 72 | 0.00655 | 12 | 0.01816 | 25 |
| a | 7 | 1 | 1.904 | 0.01046 | 62 | 0.01214 | 13 | 0.01916 | 25 |
| a | 7 | 1 | 1.916 | 0.01161 | 67 | 0.01005 | 13 | 0.01985 | 25 |
| a | 7 | 1 | 1.946 | 0.01136 | 67 | 0.0089 | 13 | 0.01791 | 25 |
| a | 7 | 1 | 1.782 | 0.0132 | 75 | 0.00469 | 8 | 0.01901 | 25 |
| a | 7 | 1 | 1.99 | 0.01235 | 71 | 0.01304 | 13 | 0.0192 | 25 |
| a | 7 | 1 | 1.924 | 0.01204 | 72 | 0.0086 | 11 | 0.01972 | 25 |
| a | 7 | 2 | 1.794 | 0.01193 | 74 | 0.00513 | 10 | 0.01889 | 25 |
| a | 7 | 2 | 1.806 | 0.01138 | 70 | 0.011 | 13 | 0.018 | 25 |
| a | 7 | 2 | 1.916 | 0.01161 | 77 | 0.00693 | 10 | 0.01889 | 25 |
| a | 7 | 2 | 1.936 | 0.01215 | 77 | 0.0052 | 8 | 0.02042 | 25 |
| a | 7 | 2 | 1.854 | 0.01196 | 66 | 0.00432 | 8 | 0.02034 | 25 |
| a | 7 | 2 | 1.958 | 0.01197 | 69 | 0.00462 | 8 | 0.01924 | 25 |
| a | 7 | 2 | 1.946 | 0.01217 | 74 | 0.00492 | 8 | 0.01971 | 25 |
| a | 7 | 2 | 1.68 | 0.01112 | 73 | 0.0036 | 9 | 0.01784 | 25 |
| a | 7 | 2 | 1.946 | 0.01025 | 63 | 0.00395 | 8 | 0.01952 | 25 |
| a | 7 | 2 | 1.76 | 0.01069 | 67 | 0.00611 | 12 | 0.02055 | 25 |
| a | 7 | 2 | 1.988 | 0.01056 | 65 | 0.00536 | 12 | 0.02008 | 25 |
| a | 7 | 3 | 1.858 | 0.01212 | 69 | 0.005 | 8 | 0.02091 | 25 |
| a | 7 | 3 | 1.884 | 0.00813 | 52 | 0.00525 | 9 | 0.0155 | 25 |
| a | 7 | 3 | 1.684 | 0.012 | 73 | 0.00437 | 9 | 0.02063 | 25 |
| a | 7 | 3 | 1.906 | 0.01185 | 68 | 0.00401 | 9 | 0.01715 | 25 |
| a | 7 | 3 | 1.972 | 0.01235 | 70 | 0.00352 | 8 | 0.01872 | 25 |
| a | 7 | 3 | 1.984 | 0.01118 | 66 | 0.00419 | 8 | 0.0202 | 25 |
| a | 7 | 3 | 2.002 | 0.01176 | 69 | 0.0044 | 9 | 0.01855 | 25 |
| a | 7 | 3 | 1.844 | 0.01049 | 65 | 0.00353 | 8 | 0.01873 | 25 |
| a | 7 | 3 | 1.769 | 0.00531 | 32 | 0.00327 | 6 | 0.01658 | 25 |
| b | 1 | 1 | 1.87 | 0.00664 | 64 | 0.00299 | 8 | 0.01094 | 10 |
| b | 1 | 1 | 1.71 | 0.00962 | 62 | 0.0041 | 10 | 0.01742 | 10 |
| b | 1 | 1 | 1.832 | 0.01294 | 71 | 0.00933 | 15 | 0.02061 | 10 |
| b | 1 | 1 | 1.746 | 0.00626 | 61 | 0.00294 | 8 | 0.01175 | 10 |
| b | 1 | 2 | 1.708 | 0.00895 | 62 | 0.00317 | 8 | 0.01734 | 10 |
| b | 1 | 2 | 1.802 | 0.00878 | 63 | 0.00568 | 11 | 0.01604 | 10 |
| b | 1 | 2 | 1.866 | 0.00713 | 66 | 0.00308 | 7 | 0.01252 | 10 |

| | | | | | | | | | |
|---|---|---|---|---|---|---|---|---|---|
| b | 1 | 3 | 1.72 | 0.00976 | 65 | 0.00459 | 9 | 0.01605 | 10 |
| b | 1 | 3 | 1.694 | 0.00933 | 65 | 0.00385 | 8 | 0.01574 | 10 |
| b | 1 | 3 | 1.662 | 0.007 | 67 | 0.00133 | 5 | 0.01068 | 10 |
| b | 2 | 1 | 1.67 | 0.00726 | 52 | 0.00247 | 8 | 0.01247 | 11 |
| b | 2 | 1 | 1.724 | 0.00875 | 64 | 0.00314 | 8 | 0.01373 | 11 |
| b | 2 | 1 | 1.768 | 0.00742 | 68 | 0.00224 | 7 | 0.01308 | 11 |
| b | 2 | 1 | 1.828 | 0.00735 | 69 | 0.00236 | 8 | 0.0107 | 11 |
| b | 2 | 2 | 1.728 | 0.00649 | 46 | 0.00519 | 10 | 0.01218 | 11 |
| b | 2 | 2 | 1.732 | 0.00909 | 68 | 0.00257 | 8 | 0.01292 | 11 |
| b | 2 | 2 | 1.768 | 0.00688 | 62 | 0.00209 | 8 | 0.01175 | 11 |
| b | 2 | 2 | 1.68 | 0.00615 | 61 | 0.0024 | 5 | 0.0105 | 11 |
| b | 2 | 3 | 1.612 | 0.00797 | 65 | 0.0031 | 8 | 0.0134 | 11 |
| b | 2 | 3 | 1.53 | 0.00978 | 68 | 0.00354 | 8 | 0.01314 | 11 |
| b | 2 | 3 | 1.604 | 0.00684 | 59 | 0.0028 | 8 | 0.01188 | 11 |
| b | 3 | 1 | 1.682 | 0.00726 | 65 | 0.00204 | 8 | 0.01736 | 9 |
| b | 3 | 1 | 1.63 | 0.00809 | 66 | 0.00202 | 8 | 0.0164 | 9 |
| b | 3 | 1 | 1.658 | 0.00612 | 59 | 0.00281 | 8 | 0.01592 | 9 |
| b | 3 | 2 | 1.602 | 0.00742 | 63 | 0.00224 | 7 | 0.01809 | 9 |
| b | 3 | 2 | 1.728 | 0.00721 | 59 | 0.00296 | 7 | 0.01528 | 9 |
| b | 3 | 2 | 1.536 | 0.00607 | 70 | 0.00149 | 8 | 0.01259 | 9 |
| b | 3 | 3 | 1.584 | 0.00761 | 67 | 0.00175 | 7 | 0.01377 | 9 |
| b | 3 | 3 | 1.586 | 0.00739 | 65 | 0.00243 | 8 | 0.01455 | 9 |
| b | 3 | 3 | 1.674 | 0.00555 | 60 | 0.00186 | 5 | 0.01312 | 9 |
| b | 4 | 1 | 1.728 | 0.00709 | 60 | 0.00221 | 8 | 0.01186 | 11 |
| b | 4 | 1 | 1.665 | 0.00754 | 64 | 0.00201 | 8 | 0.01006 | 11 |
| b | 4 | 1 | 1.702 | 0.00721 | 72 | 0.00189 | 8 | 0.01141 | 11 |
| b | 4 | 1 | 1.66 | 0.0075 | 79 | 0.00116 | 8 | 0.0104 | 11 |
| b | 4 | 2 | 1.468 | 0.00667 | 68 | 0.00085 | 8 | 0.00912 | 11 |
| b | 4 | 2 | 1.752 | 0.00677 | 73 | 0.00166 | 8 | 0.00989 | 11 |
| b | 4 | 2 | 1.612 | 0.00694 | 70 | 0.0012 | 8 | 0.00976 | 11 |
| b | 4 | 2 | 1.634 | 0.00683 | 71 | 0.00097 | 7 | 0.00983 | 11 |
| b | 4 | 3 | 1.53 | 0.00684 | 66 | 0.00058 | 5 | 0.00997 | 11 |
| b | 4 | 3 | 1.464 | 0.0069 | 70 | 0.00061 | 6 | 0.00842 | 11 |
| b | 4 | 3 | 1.564 | 0.00645 | 76 | 0.00063 | 6 | 0.00934 | 11 |
| | | | | | | | | | |
| c | 1 | 1 | 1.322 | 0.00704 | 81 | 0.00275 | 17 | 0.01025 | 9 |
| c | 1 | 1 | 1.492 | 0.00638 | 74 | 0.00366 | 20 | 0.00973 | 9 |
| c | 1 | 1 | 1.354 | 0.00636 | 76 | 0.00286 | 17 | 0.00881 | 9 |
| c | 1 | 2 | 1.252 | 0.00578 | 73 | 0.00168 | 16 | 0.00802 | 9 |
| c | 1 | 2 | 1.716 | 0.00562 | 83 | 0.00226 | 14 | 0.009 | 9 |
| c | 1 | 2 | 1.61 | 0.0062 | 82 | 0.00309 | 14 | 0.00881 | 9 |
| c | 1 | 3 | 1.694 | 0.01243 | 91 | 0.00872 | 15 | 0.01769 | 9 |
| c | 1 | 3 | 1.788 | 0.01186 | 86 | 0.0064 | 12 | 0.01745 | 9 |

| | | | | | | | | | |
|---|---|---|---|---|---|---|---|---|---|
| c | 1 | 3 | 1.69 | 0.01131 | 85 | 0.00521 | 11 | 0.01707 | 9 |
| c | 2 | 1 | 1.7 | 0.0103 | 68 | 0.0049 | 13 | 0.01805 | 15 |
| c | 2 | 1 | 1.672 | 0.01071 | 67 | 0.00536 | 15 | 0.01965 | 15 |
| c | 2 | 1 | 1.644 | 0.01128 | 71 | 0.00444 | 13 | 0.01861 | 15 |
| c | 2 | 1 | 1.682 | 0.01057 | 66 | 0.0057 | 13 | 0.01918 | 15 |
| c | 2 | 1 | 1.712 | 0.01154 | 75 | 0.00455 | 13 | 0.01876 | 15 |
| c | 2 | 2 | 1.902 | 0.00994 | 75 | 0.00474 | 11 | 0.01843 | 15 |
| c | 2 | 2 | 1.864 | 0.01227 | 75 | 0.0037 | 11 | 0.01838 | 15 |
| c | 2 | 2 | 1.576 | 0.01197 | 74 | 0.00528 | 12 | 0.0207 | 15 |
| c | 2 | 2 | 1.67 | 0.01147 | 79 | 0.00376 | 9 | 0.0168 | 15 |
| c | 2 | 2 | 1.688 | 0.01043 | 77 | 0.00361 | 9 | 0.01693 | 15 |
| c | 2 | 3 | 1.732 | 0.01215 | 76 | 0.00299 | 8 | 0.01721 | 15 |
| c | 2 | 3 | 1.452 | 0.01159 | 80 | 0.00301 | 9 | 0.01634 | 15 |
| c | 2 | 3 | 1.682 | 0.01133 | 80 | 0.0034 | 8 | 0.0185 | 15 |
| c | 2 | 3 | 1.758 | 0.01137 | 83 | 0.0037 | 8 | 0.01856 | 15 |
| c | 2 | 3 | 1.704 | 0.01036 | 70 | 0.00266 | 8 | 0.01607 | 15 |
| c | 3 | 1 | 1.87 | 0.01066 | 57 | 0.01117 | 14 | 0.0228 | 21 |
| c | 3 | 1 | 1.912 | 0.00811 | 74 | 0.01375 | 20 | 0.02052 | 21 |
| c | 3 | 1 | 1.788 | 0.00721 | 51 | 0.00902 | 15 | 0.01449 | 21 |
| c | 3 | 1 | 1.81 | 0.00597 | 48 | 0.00758 | 14 | 0.01501 | 21 |
| c | 3 | 1 | 1.786 | 0.00947 | 61 | 0.01134 | 16 | 0.01979 | 21 |
| c | 3 | 1 | 1.818 | 0.0109 | 65 | 0.0084 | 13 | 0.02531 | 21 |
| c | 3 | 1 | 1.654 | 0.0096 | 63 | 0.00515 | 12 | 0.01864 | 21 |
| c | 3 | 2 | 1.73 | 0.00681 | 52 | 0.00636 | 12 | 0.01425 | 21 |
| c | 3 | 2 | 1.88 | 0.00791 | 60 | 0.00743 | 14 | 0.01556 | 21 |
| c | 3 | 2 | 1.784 | 0.00874 | 70 | 0.00513 | 9 | 0.01721 | 21 |
| c | 3 | 2 | 1.8 | 0.00854 | 63 | 0.00349 | 8 | 0.01529 | 21 |
| c | 3 | 2 | 1.876 | 0.01023 | 65 | 0.00541 | 9 | 0.01731 | 21 |
| c | 3 | 2 | 1.816 | 0.01131 | 71 | 0.00868 | 15 | 0.02323 | 21 |
| c | 3 | 2 | 1.578 | 0.01003 | 72 | 0.00551 | 8 | 0.01801 | 21 |
| c | 3 | 3 | 1.654 | 0.00718 | 63 | 0.00452 | 9 | 0.01798 | 21 |
| c | 3 | 3 | 1.622 | 0.00916 | 65 | 0.00368 | 10 | 0.01954 | 21 |
| c | 3 | 3 | 1.572 | 0.00943 | 68 | 0.00345 | 8 | 0.01857 | 21 |
| c | 3 | 3 | 1.67 | 0.01243 | 77 | 0.00471 | 10 | 0.01798 | 21 |
| c | 3 | 3 | 1.764 | 0.01135 | 74 | 0.0047 | 9 | 0.01834 | 21 |
| c | 3 | 3 | 1.802 | 0.01145 | 74 | 0.00319 | 9 | 0.01622 | 21 |
| c | 3 | 3 | 1.726 | 0.01183 | 71 | 0.00351 | 9 | 0.01724 | 21 |
| c | 4 | 1 | 1.821 | 0.00836 | 75 | 0.01453 | 21 | 0.01892 | 12 |
| c | 4 | 1 | 1.758 | 0.00757 | 56 | 0.00932 | 16 | 0.01544 | 12 |
| c | 4 | 1 | 1.803 | 0.00558 | 49 | 0.00785 | 14 | 0.01491 | 12 |
| c | 4 | 1 | 1.768 | 0.00962 | 60 | 0.01213 | 15 | 0.01799 | 12 |
| c | 4 | 2 | 1.747 | 0.00847 | 71 | 0.00623 | 9 | 0.01644 | 12 |
| c | 4 | 2 | 1.803 | 0.00866 | 62 | 0.00431 | 8 | 0.01624 | 12 |

| | | | | | | | | | |
|---|---|---|---|---|---|---|---|---|---|
| c | 4 | 2 | 1.863 | 0.01001 | 66 | 0.00455 | 8 | 0.01811 | 12 |
| c | 4 | 2 | 1.822 | 0.01088 | 70 | 0.00756 | 14 | 0.02451 | 12 |
| c | 4 | 3 | 1.631 | 0.00924 | 66 | 0.00371 | 10 | 0.01864 | 12 |
| c | 4 | 3 | 1.603 | 0.00912 | 67 | 0.00353 | 8 | 0.01879 | 12 |
| c | 4 | 3 | 1.656 | 0.01125 | 78 | 0.00467 | 10 | 0.01764 | 12 |
| c | 4 | 3 | 1.798 | 0.01231 | 75 | 0.00488 | 9 | 0.01832 | 12 |
| d | 1 | 1 | 1.576 | 0.0098 | 64 | 0.00424 | 11 | 0.01577 | 15 |
| d | 1 | 1 | 1.52 | 0.01023 | 70 | 0.00254 | 11 | 0.01222 | 15 |
| d | 1 | 1 | 1.6 | 0.0103 | 73 | 0.00417 | 13 | 0.01372 | 15 |
| d | 1 | 1 | 1.518 | 0.01049 | 74 | 0.00495 | 13 | 0.01388 | 15 |
| d | 1 | 1 | 1.578 | 0.0103 | 73 | 0.00294 | 9 | 0.01557 | 15 |
| d | 1 | 2 | 1.722 | 0.01043 | 71 | 0.00315 | 10 | 0.01412 | 15 |
| d | 1 | 2 | 1.814 | 0.00761 | 65 | 0.00107 | 9 | 0.01199 | 15 |
| d | 1 | 2 | 1.772 | 0.00783 | 67 | 0.00102 | 7 | 0.01344 | 15 |
| d | 1 | 2 | 1.64 | 0.01095 | 74 | 0.00267 | 8 | 0.0148 | 15 |
| d | 1 | 2 | 1.604 | 0.01028 | 79 | 0.00232 | 8 | 0.01298 | 15 |
| d | 1 | 3 | 1.558 | 0.01027 | 80 | 0.0029 | 7 | 0.0141 | 15 |
| d | 1 | 3 | 1.73 | 0.01016 | 80 | 0.00396 | 8 | 0.01338 | 15 |
| d | 1 | 3 | 1.648 | 0.01026 | 75 | 0.00332 | 8 | 0.01412 | 15 |
| d | 1 | 3 | 1.52 | 0.01039 | 73 | 0.00312 | 8 | 0.01489 | 15 |
| d | 1 | 3 | 1.72 | 0.01001 | 74 | 0.00473 | 8 | 0.01503 | 15 |
| d | 2 | 1 | 1.756 | 0.0107 | 65 | 0.00383 | 8 | 0.01362 | 13 |
| d | 2 | 1 | 1.716 | 0.00783 | 59 | 0.00303 | 8 | 0.01245 | 13 |
| d | 2 | 1 | 1.64 | 0.00893 | 68 | 0.00261 | 8 | 0.01314 | 13 |
| d | 2 | 1 | 1.73 | 0.00834 | 95 | 0.00322 | 8 | 0.01266 | 13 |
| d | 2 | 2 | 1.834 | 0.0095 | 74 | 0.00252 | 8 | 0.01298 | 13 |
| d | 2 | 2 | 1.738 | 0.00968 | 69 | 0.00209 | 8 | 0.0137 | 13 |
| d | 2 | 2 | 1.658 | 0.01012 | 69 | 0.00211 | 7 | 0.01458 | 13 |
| d | 2 | 2 | 1.868 | 0.00961 | 65 | 0.0021 | 6 | 0.01468 | 13 |
| d | 2 | 2 | 1.868 | 0.01062 | 70 | 0.00186 | 7 | 0.01399 | 13 |
| d | 2 | 3 | 1.768 | 0.00993 | 74 | 0.0019 | 7 | 0.01459 | 13 |
| d | 2 | 3 | 1.576 | 0.01011 | 74 | 0.00194 | 8 | 0.01487 | 13 |
| d | 2 | 3 | 1.88 | 0.00961 | 76 | 0.00231 | 8 | 0.01516 | 13 |
| d | 2 | 3 | 1.786 | 0.00833 | 78 | 0.00337 | 8 | 0.0134 | 13 |
| d | 3 | 1 | 1.54 | 0.01117 | 79 | 0.00512 | 11 | 0.01617 | 14 |
| d | 3 | 1 | 1.516 | 0.00977 | 69 | 0.00556 | 11 | 0.01657 | 14 |
| d | 3 | 1 | 1.576 | 0.00967 | 73 | 0.00468 | 13 | 0.01552 | 14 |
| d | 3 | 1 | 1.732 | 0.01012 | 75 | 0.00381 | 10 | 0.01458 | 14 |
| d | 3 | 2 | 1.62 | 0.00999 | 55 | 0.00502 | 13 | 0.01424 | 14 |
| d | 3 | 2 | 1.608 | 0.00867 | 74 | 0.00397 | 12 | 0.01375 | 14 |
| d | 3 | 2 | 1.644 | 0.00862 | 74 | 0.0037 | 10 | 0.01532 | 14 |
| d | 3 | 2 | 1.48 | 0.00959 | 74 | 0.00284 | 9 | 0.0156 | 14 |
| d | 3 | 2 | 1.704 | 0.00989 | 76 | 0.00302 | 8 | 0.01629 | 14 |

| | | | | | | | | | |
|---|---|---|---|---|---|---|---|---|---|
| d | 3 | 2 | 1.604 | 0.00809 | 63 | 0.00281 | 9 | 0.01452 | 14 |
| d | 3 | 3 | 1.62 | 0.00883 | 67 | 0.00399 | 10 | 0.01877 | 14 |
| d | 3 | 3 | 1.558 | 0.01023 | 77 | 0.00283 | 9 | 0.01675 | 14 |
| d | 3 | 3 | 1.556 | 0.0078 | 64 | 0.00498 | 13 | 0.01337 | 14 |
| d | 3 | 3 | 1.658 | 0.01043 | 81 | 0.00426 | 13 | 0.01616 | 14 |
| d | 5 | 1 | 1.612 | 0.00947 | 63 | 0.00328 | 9 | 0.01337 | 11 |
| d | 5 | 1 | 1.534 | 0.00941 | 64 | 0.00489 | 10 | 0.01348 | 11 |
| d | 5 | 1 | 1.608 | 0.00983 | 66 | 0.0034 | 9 | 0.01461 | 11 |
| d | 5 | 2 | 1.506 | 0.00978 | 62 | 0.00288 | 8 | 0.01361 | 11 |
| d | 5 | 2 | 1.44 | 0.00935 | 68 | 0.00261 | 8 | 0.01335 | 11 |
| d | 5 | 2 | 1.402 | 0.01057 | 72 | 0.0027 | 8 | 0.01322 | 11 |
| d | 5 | 2 | 1.638 | 0.00914 | 69 | 0.00351 | 9 | 0.01216 | 11 |
| d | 5 | 2 | 1.608 | 0.00875 | 68 | 0.00265 | 8 | 0.01212 | 11 |
| d | 5 | 3 | 1.594 | 0.00926 | 72 | 0.00314 | 8 | 0.01492 | 11 |
| d | 5 | 3 | 1.534 | 0.00935 | 72 | 0.00298 | 6 | 0.01334 | 11 |
| d | 5 | 3 | 1.536 | 0.00892 | 68 | 0.00261 | 8 | 0.01432 | 11 |
| e | 1 | 1 | 1.724 | 0.01055 | 72 | 0.00339 | 11 | 0.01468 | 9 |
| e | 1 | 1 | 1.914 | 0.00945 | 65 | 0.00455 | 10 | 0.0141 | 9 |
| e | 1 | 1 | 1.83 | 0.01025 | 67 | 0.00419 | 9 | 0.0146 | 9 |
| e | 1 | 2 | 1.774 | 0.00915 | 61 | 0.00497 | 11 | 0.01766 | 9 |
| e | 1 | 2 | 1.756 | 0.01047 | 69 | 0.00309 | 10 | 0.01574 | 9 |
| e | 1 | 2 | 1.736 | 0.00947 | 73 | 0.00289 | 8 | 0.01369 | 9 |
| e | 1 | 3 | 1.692 | 0.00972 | 73 | 0.00398 | 8 | 0.01383 | 9 |
| e | 1 | 3 | 1.654 | 0.00962 | 72 | 0.00313 | 8 | 0.01531 | 9 |
| e | 1 | 3 | 1.516 | 0.0089 | 65 | 0.00289 | 8 | 0.01525 | 9 |
| e | 2 | 1 | 1.572 | 0.00953 | 73 | 0.00498 | 12 | 0.01512 | 14 |
| e | 2 | 1 | 1.546 | 0.00982 | 72 | 0.00468 | 13 | 0.01553 | 14 |
| e | 2 | 1 | 1.548 | 0.00961 | 74 | 0.0046 | 13 | 0.01495 | 14 |
| e | 2 | 1 | 1.53 | 0.01002 | 78 | 0.00474 | 13 | 0.01469 | 14 |
| e | 2 | 2 | 1.644 | 0.00913 | 73 | 0.00326 | 8 | 0.01525 | 14 |
| e | 2 | 2 | 1.718 | 0.01014 | 74 | 0.00232 | 10 | 0.01519 | 14 |
| e | 2 | 2 | 1.53 | 0.00982 | 77 | 0.00228 | 9 | 0.01427 | 14 |
| e | 2 | 2 | 1.556 | 0.00993 | 72 | 0.00225 | 7 | 0.01253 | 14 |
| e | 2 | 2 | 1.576 | 0.0095 | 77 | 0.00239 | 8 | 0.01365 | 14 |
| e | 2 | 2 | 1.478 | 0.00989 | 79 | 0.00274 | 8 | 0.01359 | 14 |
| e | 2 | 3 | 1.576 | 0.0076 | 71 | 0.00311 | 8 | 0.01212 | 14 |
| e | 2 | 3 | 1.432 | 0.00929 | 77 | 0.00299 | 8 | 0.01369 | 14 |
| e | 2 | 3 | 1.598 | 0.00843 | 77 | 0.00403 | 8 | 0.01272 | 14 |
| e | 2 | 3 | 1.684 | 0.00837 | 77 | 0.00274 | 7 | 0.01311 | 14 |
| e | 3 | 1 | 1.806 | 0.01133 | 80 | 0.00641 | 15 | 0.01698 | 12 |
| e | 3 | 1 | 1.79 | 0.01134 | 81 | 0.00556 | 13 | 0.01762 | 12 |
| e | 3 | 1 | 1.76 | 0.01202 | 82 | 0.00524 | 15 | 0.01774 | 12 |
| e | 3 | 1 | 1.95 | 0.01129 | 73 | 0.00905 | 14 | 0.01818 | 12 |

| | | | | | | | | | |
|---|---|---|---|---|---|---|---|---|---|
| e | 3 | 2 | 1.844 | 0.01245 | 86 | 0.00611 | 14 | 0.01906 | 12 |
| e | 3 | 2 | 1.788 | 0.01219 | 85 | 0.00481 | 13 | 0.01775 | 12 |
| e | 3 | 2 | 1.774 | 0.01274 | 84 | 0.00527 | 12 | 0.019 | 12 |
| e | 3 | 2 | 1.658 | 0.01198 | 84 | 0.00405 | 11 | 0.0176 | 12 |
| e | 3 | 3 | 1.85 | 0.01266 | 88 | 0.00617 | 13 | 0.01918 | 12 |
| e | 3 | 3 | 1.556 | 0.00464 | 33 | 0.00226 | 6 | 0.01173 | 12 |
| e | 3 | 3 | 1.804 | 0.00876 | 61 | 0.00323 | 7 | 0.01648 | 12 |
| e | 3 | 3 | 1.776 | 0.0063 | 50 | 0.00334 | 6 | 0.01645 | 12 |
| e | 4 | 1 | 1.692 | 0.00755 | 65 | 0.00394 | 10 | 0.01398 | 17 |
| e | 4 | 1 | 1.722 | 0.00805 | 71 | 0.00296 | 13 | 0.01366 | 17 |
| e | 4 | 1 | 1.584 | 0.00833 | 67 | 0.00465 | 13 | 0.01395 | 17 |
| e | 4 | 1 | 1.664 | 0.00785 | 71 | 0.00539 | 13 | 0.01359 | 17 |
| e | 4 | 1 | 1.638 | 0.00857 | 71 | 0.00512 | 13 | 0.01351 | 17 |
| e | 4 | 2 | 1.714 | 0.00818 | 68 | 0.00461 | 12 | 0.01316 | 17 |
| e | 4 | 2 | 1.51 | 0.00892 | 75 | 0.00279 | 9 | 0.01247 | 17 |
| e | 4 | 2 | 1.628 | 0.00861 | 75 | 0.0035 | 11 | 0.01283 | 17 |
| e | 4 | 2 | 1.694 | 0.00782 | 71 | 0.00266 | 8 | 0.01188 | 17 |
| e | 4 | 2 | 1.506 | 0.00787 | 76 | 0.00367 | 11 | 0.01229 | 17 |
| e | 4 | 2 | 1.56 | 0.00765 | 72 | 0.00251 | 9 | 0.01242 | 17 |
| e | 4 | 2 | 1.636 | 0.01109 | 80 | 0.0063 | 10 | 0.01363 | 17 |
| e | 4 | 3 | 1.886 | 0.01235 | 85 | 0.00549 | 11 | 0.01582 | 17 |
| e | 4 | 3 | 1.57 | 0.0097 | 72 | 0.00372 | 8 | 0.01475 | 17 |
| e | 4 | 3 | 1.644 | 0.00127 | 83 | 0.00493 | 11 | 0.01641 | 17 |
| e | 4 | 3 | 1.542 | 0.00742 | 69 | 0.00399 | 8 | 0.01384 | 17 |
| e | 4 | 3 | 1.702 | 0.00678 | 62 | 0.00233 | 8 | 0.01354 | 17 |
| f | 1 | 1 | 1.904 | 0.0092 | 65 | 0.00798 | 15 | 0.01465 | 15 |
| f | 1 | 1 | 1.872 | 0.00918 | 65 | 0.00728 | 15 | 0.01419 | 15 |
| f | 1 | 1 | 1.71 | 0.01015 | 69 | 0.00587 | 13 | 0.01424 | 15 |
| f | 1 | 1 | 1.658 | 0.01056 | 69 | 0.00559 | 12 | 0.01469 | 15 |
| f | 1 | 1 | 2.032 | 0.0095 | 70 | 0.00492 | 9 | 0.01799 | 15 |
| f | 1 | 2 | 1.968 | 0.01161 | 77 | 0.00413 | 8 | 0.01851 | 15 |
| f | 1 | 2 | 1.542 | 0.00649 | 57 | 0.00358 | 10 | 0.01312 | 15 |
| f | 1 | 2 | 1.716 | 0.00848 | 68 | 0.00444 | 9 | 0.01432 | 15 |
| f | 1 | 2 | 1.63 | 0.00983 | 70 | 0.00556 | 11 | 0.01445 | 15 |
| f | 1 | 2 | 1.556 | 0.0041 | 41 | 0.00385 | 7 | 0.00844 | 15 |
| f | 1 | 3 | 1.732 | 0.00896 | 66 | 0.00533 | 10 | 0.01426 | 15 |
| f | 1 | 3 | 1.746 | 0.00838 | 65 | 0.00737 | 10 | 0.01241 | 15 |
| f | 1 | 3 | 1.75 | 0.01008 | 68 | 0.00496 | 9 | 0.01622 | 15 |
| f | 1 | 3 | 1.648 | 0.00911 | 64 | 0.00341 | 8 | 0.01539 | 15 |
| f | 1 | 3 | 1.708 | 0.00932 | 64 | 0.00323 | 8 | 0.01525 | 15 |
| f | 2 | 1 | 1.756 | 0.00775 | 62 | 0.00244 | 9 | 0.01225 | 13 |
| f | 2 | 1 | 1.7 | 0.00794 | 66 | 0.00361 | 9 | 0.01268 | 13 |
| f | 2 | 1 | 1.622 | 0.00825 | 62 | 0.00234 | 8 | 0.01283 | 13 |

| | | | | | | | | | |
|---|---|---|---|---|---|---|---|---|---|
| f | 2 | 1 | 1.67 | 0.00766 | 62 | 0.00369 | 13 | 0.01211 | 13 |
| f | 2 | 2 | 1.7 | 0.00812 | 59 | 0.0019 | 7 | 0.01226 | 13 |
| f | 2 | 2 | 1.684 | 0.00916 | 68 | 0.00182 | 8 | 0.01328 | 13 |
| f | 2 | 2 | 1.732 | 0.00863 | 60 | 0.00238 | 8 | 0.01332 | 13 |
| f | 2 | 2 | 1.754 | 0.00799 | 63 | 0.00268 | 8 | 0.01298 | 13 |
| f | 2 | 2 | 1.87 | 0.00785 | 64 | 0.00208 | 7 | 0.01264 | 13 |
| f | 2 | 3 | 1.564 | 0.00681 | 68 | 0.00415 | 8 | 0.01257 | 13 |
| f | 2 | 3 | 1.704 | 0.00366 | 58 | 0.00658 | 8 | 0.0118 | 13 |
| f | 2 | 3 | 1.702 | 0.01012 | 71 | 0.00483 | 9 | 0.01467 | 13 |
| f | 2 | 3 | 1.684 | 0.00962 | 70 | 0.00297 | 8 | 0.0134 | 13 |
| f | 4 | 1 | 1.726 | 0.01127 | 69 | 0.00565 | 9 | 0.01678 | 15 |
| f | 4 | 1 | 1.858 | 0.0107 | 69 | 0.01017 | 13 | 0.0163 | 15 |
| f | 4 | 1 | 1.914 | 0.01046 | 69 | 0.00537 | 9 | 0.01592 | 15 |
| f | 4 | 1 | 1.982 | 0.01143 | 72 | 0.00435 | 8 | 0.01736 | 15 |
| f | 4 | 1 | 1.862 | 0.01042 | 68 | 0.00601 | 10 | 0.01761 | 15 |
| f | 4 | 2 | 1.89 | 0.01149 | 75 | 0.00434 | 8 | 0.019 | 15 |
| f | 4 | 2 | 1.752 | 0.01042 | 68 | 0.0052 | 13 | 0.01483 | 15 |
| f | 4 | 2 | 1.754 | 0.00972 | 70 | 0.00607 | 12 | 0.01524 | 15 |
| f | 4 | 2 | 1.752 | 0.00971 | 70 | 0.00519 | 13 | 0.01517 | 15 |
| f | 4 | 2 | 1.89 | 0.01192 | 74 | 0.00471 | 8 | 0.01977 | 15 |
| f | 4 | 3 | 1.896 | 0.01069 | 71 | 0.0045 | 8 | 0.0157 | 15 |
| f | 4 | 3 | 1.688 | 0.00639 | 61 | 0.00272 | 8 | 0.02187 | 15 |
| f | 4 | 3 | 1.63 | 0.00648 | 63 | 0.00241 | 8 | 0.0107 | 15 |
| f | 4 | 3 | 1.698 | 0.0074 | 69 | 0.00189 | 7 | 0.01124 | 15 |
| f | 4 | 3 | 1.472 | 0.00672 | 65 | 0.00145 | 8 | 0.01097 | 15 |
| f | 5 | 1 | 1.702 | 0.00855 | 67 | 0.00149 | 8 | 0.01114 | 11 |
| f | 5 | 1 | 1.742 | 0.00861 | 70 | 0.00224 | 8 | 0.01041 | 11 |
| f | 5 | 1 | 1.708 | 0.00817 | 66 | 0.00259 | 8 | 0.01086 | 11 |
| f | 5 | 2 | 1.588 | 0.00821 | 70 | 0.00228 | 9 | 0.00982 | 11 |
| f | 5 | 2 | 1.696 | 0.00822 | 70 | 0.00216 | 8 | 0.01008 | 11 |
| f | 5 | 2 | 1.602 | 0.0081 | 76 | 0.00179 | 8 | 0.01114 | 11 |
| f | 5 | 2 | 1.59 | 0.00861 | 74 | 0.00148 | 8 | 0.0109 | 11 |
| f | 5 | 2 | 1.622 | 0.00801 | 69 | 0.00319 | 8 | 0.01275 | 11 |
| f | 5 | 3 | 1.662 | 0.00805 | 65 | 0.00372 | 8 | 0.01218 | 11 |
| f | 5 | 3 | 1.542 | 0.00781 | 74 | 0.00136 | 8 | 0.01072 | 11 |
| f | 5 | 3 | 1.732 | 0.00806 | 73 | 0.00212 | 8 | 0.012 | 11 |
| g | 1 | 1 | 1.718 | 0.00914 | 70 | 0.00266 | 8 | 0.01575 | 23 |
| g | 1 | 1 | 1.664 | 0.00751 | 67 | 0.00243 | 9 | 0.01449 | 23 |
| g | 1 | 1 | 2.008 | 0.00729 | 45 | 0.00632 | 11 | 0.01425 | 23 |
| g | 1 | 1 | 2.018 | 0.00613 | 40 | 0.00562 | 11 | 0.0125 | 23 |
| g | 1 | 1 | 1.66 | 0.00514 | 63 | 0.00235 | 7 | 0.0107 | 23 |
| g | 1 | 1 | 1.762 | 0.00911 | 76 | 0.00263 | 8 | 0.01937 | 23 |
| g | 1 | 1 | 1.846 | 0.0101 | 72 | 0.00819 | 13 | 0.01694 | 23 |

| | | | | | | | | | |
|---|---|---|---|---|---|---|---|---|---|
| g | 1 | 2 | 1.83 | 0.00967 | 72 | 0.00619 | 13 | 0.01421 | 23 |
| g | 1 | 2 | 1.908 | 0.01167 | 83 | 0.00517 | 13 | 0.01867 | 23 |
| g | 1 | 2 | 1.728 | 0.01157 | 80 | 0.00373 | 11 | 0.01638 | 23 |
| g | 1 | 2 | 1.838 | 0.01226 | 84 | 0.00558 | 13 | 0.01763 | 23 |
| g | 1 | 2 | 1.586 | 0.01094 | 75 | 0.00442 | 12 | 0.01662 | 23 |
| g | 1 | 2 | 1.876 | 0.01265 | 86 | 0.00457 | 13 | 0.01741 | 23 |
| g | 1 | 2 | 1.692 | 0.0101 | 83 | 0.00437 | 12 | 0.01461 | 23 |
| g | 1 | 2 | 1.776 | 0.01131 | 83 | 0.0034 | 9 | 0.01693 | 23 |
| g | 1 | 2 | 1.78 | 0.01214 | 87 | 0.00388 | 10 | 0.01579 | 23 |
| g | 1 | 3 | 1.75 | 0.01221 | 85 | 0.00356 | 10 | 0.01544 | 23 |
| g | 1 | 3 | 1.736 | 0.01167 | 84 | 0.00355 | 9 | 0.01601 | 23 |
| g | 1 | 3 | 1.564 | 0.0102 | 81 | 0.00274 | 8 | 0.01549 | 23 |
| g | 1 | 3 | 1.38 | 0.00995 | 81 | 0.00279 | 8 | 0.01607 | 23 |
| g | 1 | 3 | 1.818 | 0.01124 | 87 | 0.00298 | 8 | 0.01298 | 23 |
| g | 1 | 3 | 1.882 | 0.01107 | 85 | 0.00346 | 8 | 0.01521 | 23 |
| g | 1 | 3 | 1.426 | 0.00774 | 74 | 0.00166 | 8 | 0.01126 | 23 |
| g | 2 | 1 | 1.696 | 0.00858 | 62 | 0.00255 | 8 | 0.01533 | 15 |
| g | 2 | 1 | 1.768 | 0.00803 | 65 | 0.00276 | 8 | 0.01364 | 15 |
| g | 2 | 1 | 1.75 | 0.00965 | 73 | 0.00195 | 8 | 0.01216 | 15 |
| g | 2 | 1 | 1.732 | 0.00971 | 71 | 0.00162 | 8 | 0.01256 | 15 |
| g | 2 | 1 | 1.664 | 0.00731 | 60 | 0.00265 | 8 | 0.01145 | 15 |
| g | 2 | 2 | 1.682 | 0.00792 | 61 | 0.00273 | 8 | 0.01205 | 15 |
| g | 2 | 2 | 1.624 | 0.00769 | 61 | 0.00206 | 8 | 0.01154 | 15 |
| g | 2 | 2 | 1.658 | 0.00676 | 55 | 0.00378 | 8 | 0.01179 | 15 |
| g | 2 | 2 | 1.678 | 0.008 | 61 | 0.00277 | 8 | 0.01316 | 15 |
| g | 2 | 2 | 1.728 | 0.00827 | 62 | 0.00269 | 8 | 0.01234 | 15 |
| g | 2 | 3 | 1.702 | 0.00806 | 57 | 0.00158 | 8 | 0.01252 | 15 |
| g | 2 | 3 | 1.602 | 0.00765 | 64 | 0.00185 | 8 | 0.0125 | 15 |
| g | 2 | 3 | 1.792 | 0.00796 | 67 | 0.00208 | 7 | 0.01234 | 15 |
| g | 2 | 3 | 1.758 | 0.00813 | 65 | 0.00221 | 8 | 0.012 | 15 |
| g | 2 | 3 | 1.794 | 0.00803 | 76 | 0.00162 | 6 | 0.01203 | 15 |
| g | 3 | 1 | 1.848 | 0.00656 | 46 | 0.00531 | 8 | 0.01437 | 12 |
| g | 3 | 1 | 1.968 | 0.00729 | 45 | 0.00414 | 8 | 0.01413 | 12 |
| g | 3 | 1 | 1.93 | 0.00836 | 56 | 0.00628 | 14 | 0.01423 | 12 |
| g | 3 | 1 | 1.678 | 0.00424 | 32 | 0.00457 | 8 | 0.01275 | 12 |
| g | 3 | 2 | 1.61 | 0.00521 | 38 | 0.00364 | 5 | 0.01169 | 12 |
| g | 3 | 2 | 1.898 | 0.00775 | 59 | 0.00669 | 12 | 0.01295 | 12 |
| g | 3 | 2 | 1.698 | 0.00575 | 39 | 0.00574 | 10 | 0.01663 | 12 |
| g | 3 | 2 | 1.812 | 0.00665 | 48 | 0.00412 | 11 | 0.01294 | 12 |
| g | 3 | 3 | 1.654 | 0.00843 | 61 | 0.00257 | 8 | 0.01528 | 12 |
| g | 3 | 3 | 1.91 | 0.00841 | 55 | 0.00912 | 14 | 0.01813 | 12 |
| g | 3 | 3 | 1.576 | 0.00913 | 61 | 0.00439 | 10 | 0.01718 | 12 |
| g | 3 | 3 | 2.07 | 0.00804 | 61 | 0.00353 | 8 | 0.0184 | 12 |

| | | | | | | | | | |
|---|---|---|---|---|---|---|---|---|---|
| g | 4 | 1 | 1.634 | 0.00783 | 61 | 0.00149 | 7 | 0.0134 | 11 |
| g | 4 | 1 | 1.738 | 0.00666 | 54 | 0.00104 | 7 | 0.01106 | 11 |
| g | 4 | 1 | 1.788 | 0.0078 | 62 | 0.00115 | 7 | 0.01225 | 11 |
| g | 4 | 2 | 1.76 | 0.00747 | 65 | 0.00107 | 7 | 0.01114 | 11 |
| g | 4 | 2 | 1.648 | 0.0072 | 67 | 0.00103 | 8 | 0.01141 | 11 |
| g | 4 | 2 | 1.732 | 0.00727 | 62 | 0.00099 | 7 | 0.01168 | 11 |
| g | 4 | 2 | 1.716 | 0.00804 | 69 | 0.00073 | 6 | 0.01185 | 11 |
| g | 4 | 2 | 1.754 | 0.00752 | 69 | 0.0009 | 6 | 0.01169 | 11 |
| g | 4 | 3 | 1.66 | 0.00699 | 70 | 0.00126 | 6 | 0.01154 | 11 |
| g | 4 | 3 | 1.608 | 0.00702 | 69 | 0.00105 | 5 | 0.01083 | 11 |
| g | 4 | 3 | 1.662 | 0.00686 | 70 | 0.00108 | 5 | 0.01116 | 11 |
| h | 1 | 1 | 1.892 | 0.00796 | 64 | 0.00262 | 8 | 0.01273 | 16 |
| h | 1 | 1 | 1.43 | 0.0101 | 79 | 0.00685 | 11 | 0.01347 | 16 |
| h | 1 | 1 | 1.494 | 0.00939 | 74 | 0.00482 | 13 | 0.01347 | 16 |
| h | 1 | 1 | 1.496 | 0.00963 | 73 | 0.00757 | 14 | 0.01402 | 16 |
| h | 1 | 1 | 1.556 | 0.00854 | 86 | 0.00532 | 13 | 0.01208 | 16 |
| h | 1 | 2 | 1.682 | 0.01046 | 86 | 0.00572 | 13 | 0.01292 | 16 |
| h | 1 | 2 | 1.522 | 0.00676 | 80 | 0.00474 | 12 | 0.01265 | 16 |
| h | 1 | 2 | 1.498 | 0.01109 | 85 | 0.0049 | 13 | 0.01288 | 16 |
| h | 1 | 2 | 1.402 | 0.00966 | 86 | 0.00506 | 11 | 0.01064 | 16 |
| h | 1 | 2 | 1.528 | 0.00938 | 90 | 0.00514 | 12 | 0.01074 | 16 |
| h | 1 | 2 | 1.418 | 0.0104 | 90 | 0.00381 | 10 | 0.0132 | 16 |
| h | 1 | 3 | 1.48 | 0.00977 | 84 | 0.00362 | 11 | 0.01032 | 16 |
| h | 1 | 3 | 1.438 | 0.01064 | 89 | 0.00344 | 10 | 0.01373 | 16 |
| h | 1 | 3 | 1.494 | 0.01064 | 91 | 0.00245 | 8 | 0.01326 | 16 |
| h | 1 | 3 | 1.314 | 0.01086 | 95 | 0.00212 | 8 | 0.01408 | 16 |
| h | 1 | 3 | 1.356 | 0.01015 | 88 | 0.00125 | 8 | 0.01014 | 16 |
| h | 2 | 1 | 1.694 | 0.00716 | 55 | 0.00237 | 8 | 0.01035 | 18 |
| h | 2 | 1 | 1.628 | 0.00691 | 61 | 0.00171 | 8 | 0.01073 | 18 |
| h | 2 | 1 | 1.478 | 0.00933 | 62 | 0.00736 | 14 | 0.01516 | 18 |
| h | 2 | 1 | 1.536 | 0.00956 | 67 | 0.00507 | 13 | 0.01365 | 18 |
| h | 2 | 1 | 1.566 | 0.00935 | 64 | 0.0071 | 16 | 0.01528 | 18 |
| h | 2 | 1 | 1.544 | 0.00909 | 68 | 0.00337 | 11 | 0.01264 | 18 |
| h | 2 | 2 | 1.542 | 0.0102 | 75 | 0.00394 | 13 | 0.01373 | 18 |
| h | 2 | 2 | 1.552 | 0.00952 | 74 | 0.00473 | 13 | 0.01365 | 18 |
| h | 2 | 2 | 1.602 | 0.01098 | 71 | 0.00433 | 12 | 0.0158 | 18 |
| h | 2 | 2 | 1.53 | 0.01081 | 75 | 0.00395 | 13 | 0.01542 | 18 |
| h | 2 | 2 | 1.608 | 0.01032 | 70 | 0.00466 | 12 | 0.01564 | 18 |
| h | 2 | 2 | 1.576 | 0.01036 | 82 | 0.00274 | 9 | 0.01535 | 18 |
| h | 2 | 3 | 1.41 | 0.01009 | 71 | 0.00245 | 9 | 0.01503 | 18 |
| h | 2 | 3 | 1.434 | 0.01008 | 74 | 0.00406 | 13 | 0.01438 | 18 |
| h | 2 | 3 | 1.484 | 0.00969 | 62 | 0.00651 | 16 | 0.01667 | 18 |
| h | 2 | 3 | 1.512 | 0.01071 | 72 | 0.00477 | 13 | 0.01452 | 18 |

| | | | | | | | | | |
|---|---|---|---|---|---|---|---|---|---|
| h | 2 | 3 | 1.528 | 0.0077 | 65 | 0.00233 | 9 | 0.01336 | 18 |
| h | 2 | 3 | 1.468 | 0.00986 | 69 | 0.00326 | 8 | 0.01585 | 18 |
| h | 3 | 1 | 1.726 | 0.00668 | 54 | 0.00245 | 8 | 0.01185 | 6 |
| h | 3 | 1 | 1.828 | 0.00743 | 67 | 0.00221 | 8 | 0.01258 | 6 |
| h | 3 | 2 | 1.722 | 0.00726 | 61 | 0.00205 | 8 | 0.01274 | 6 |
| h | 3 | 2 | 1.77 | 0.00792 | 64 | 0.00155 | 8 | 0.01286 | 6 |
| h | 3 | 3 | 1.864 | 0.00764 | 62 | 0.00195 | 8 | 0.0175 | 6 |
| h | 3 | 3 | 1.69 | 0.00837 | 68 | 0.0018 | 7 | 0.0128 | 6 |
| h | 4 | 1 | 1.474 | 0.01093 | 70 | 0.00764 | 13 | 0.01779 | 22 |
| h | 4 | 1 | 1.63 | 0.01075 | 74 | 0.00639 | 15 | 0.01688 | 22 |
| h | 4 | 1 | 1.654 | 0.01002 | 76 | 0.00868 | 15 | 0.0171 | 22 |
| h | 4 | 1 | 1.606 | 0.01112 | 77 | 0.00613 | 13 | 0.01592 | 22 |
| h | 4 | 1 | 1.606 | 0.01113 | 73 | 0.00612 | 13 | 0.01632 | 22 |
| h | 4 | 1 | 1.568 | 0.01109 | 72 | 0.00574 | 14 | 0.01823 | 22 |
| h | 4 | 1 | 1.614 | 0.01159 | 76 | 0.00766 | 14 | 0.01856 | 22 |
| h | 4 | 2 | 1.638 | 0.01173 | 79 | 0.00601 | 15 | 0.01577 | 22 |
| h | 4 | 2 | 1.408 | 0.01044 | 78 | 0.00472 | 13 | 0.00945 | 22 |
| h | 4 | 2 | 1.442 | 0.01108 | 78 | 0.0049 | 12 | 0.01549 | 22 |
| h | 4 | 2 | 1.49 | 0.01217 | 80 | 0.00456 | 11 | 0.01515 | 22 |
| h | 4 | 2 | 1.526 | 0.01037 | 73 | 0.00434 | 13 | 0.01514 | 22 |
| h | 4 | 2 | 1.416 | 0.0087 | 74 | 0.00393 | 11 | 0.01567 | 22 |
| h | 4 | 2 | 1.428 | 0.0097 | 81 | 0.00372 | 11 | 0.01667 | 22 |
| h | 4 | 2 | 1.476 | 0.01149 | 78 | 0.00535 | 13 | 0.01627 | 22 |
| h | 4 | 3 | 1.546 | 0.01032 | 80 | 0.00499 | 12 | 0.01443 | 22 |
| h | 4 | 3 | 1.49 | 0.00995 | 83 | 0.00589 | 14 | 0.01298 | 22 |
| h | 4 | 3 | 1.356 | 0.00622 | 63 | 0.00175 | 7 | 0.0108 | 22 |
| h | 4 | 3 | 1.442 | 0.00925 | 79 | 0.00398 | 10 | 0.01477 | 22 |
| h | 4 | 3 | 1.546 | 0.01029 | 84 | 0.00428 | 9 | 0.01364 | 22 |
| h | 4 | 3 | 1.342 | 0.00457 | 47 | 0.00122 | 4 | 0.01077 | 22 |
| h | 4 | 3 | 1.318 | 0.00966 | 76 | 0.00395 | 8 | 0.01798 | 22 |
| i | 1 | 1 | 1.876 | 0.00724 | 55 | 0.00355 | 8 | 0.01264 | 9 |
| i | 1 | 1 | 1.61 | 0.00832 | 61 | 0.0024 | 8 | 0.01285 | 9 |
| i | 1 | 1 | 1.68 | 0.00804 | 60 | 0.00172 | 7 | 0.01288 | 9 |
| i | 1 | 2 | 1.706 | 0.00809 | 60 | 0.00127 | 6 | 0.01233 | 9 |
| i | 1 | 2 | 1.65 | 0.00812 | 68 | 0.001 | 5 | 0.01222 | 9 |
| i | 1 | 2 | 1.626 | 0.0078 | 61 | 0.00145 | 5 | 0.01295 | 9 |
| i | 1 | 3 | 1.628 | 0.00774 | 57 | 0.00121 | 5 | 0.01179 | 9 |
| i | 1 | 3 | 1.916 | 0.0077 | 60 | 0.00184 | 6 | 0.01242 | 9 |
| i | 1 | 3 | 1.688 | 0.00631 | 52 | 0.00147 | 5 | 0.01154 | 9 |
| i | 2 | 1 | 1.684 | 0.00779 | 69 | 0.00251 | 5 | 0.01547 | 10 |
| i | 2 | 1 | 1.438 | 0.00686 | 69 | 0.0022 | 5 | 0.01487 | 10 |
| i | 2 | 1 | 1.567 | 0.00797 | 68 | 0.00243 | 5 | 0.01474 | 10 |
| i | 2 | 1 | 1.478 | 0.00716 | 66 | 0.00231 | 5 | 0.01513 | 10 |

| | | | | | | | | | |
|---|---|---|---|---|---|---|---|---|---|
| i | 2 | 2 | 1.602 | 0.00872 | 65 | 0.00217 | 6 | 0.01581 | 10 |
| i | 2 | 2 | 1.632 | 0.00842 | 67 | 0.00223 | 6 | 0.01477 | 10 |
| i | 2 | 2 | 1.564 | 0.00795 | 64 | 0.00204 | 5 | 0.01656 | 10 |
| i | 2 | 3 | 1.543 | 0.00782 | 66 | 0.00264 | 6 | 0.01589 | 10 |
| i | 2 | 3 | 1.46 | 0.0078 | 69 | 0.0021 | 5 | 0.01544 | 10 |
| i | 2 | 3 | 1.474 | 0.00966 | 68 | 0.00259 | 5 | 0.01665 | 10 |
| i | 3 | 1 | 1.632 | 0.00845 | 70 | 0.00237 | 7 | 0.01147 | 12 |
| i | 3 | 1 | 1.582 | 0.00886 | 73 | 0.0018 | 8 | 0.01189 | 12 |
| i | 3 | 1 | 1.672 | 0.00914 | 69 | 0.00148 | 6 | 0.01276 | 12 |
| i | 3 | 1 | 1.534 | 0.00904 | 72 | 0.00149 | 7 | 0.01186 | 12 |
| i | 3 | 2 | 1.67 | 0.00878 | 71 | 0.00105 | 8 | 0.01188 | 12 |
| i | 3 | 2 | 1.332 | 0.00871 | 69 | 0.00163 | 8 | 0.01173 | 12 |
| i | 3 | 2 | 1.436 | 0.00872 | 70 | 0.00096 | 5 | 0.01151 | 12 |
| i | 3 | 2 | 1.58 | 0.00897 | 75 | 0.00144 | 7 | 0.011 | 12 |
| i | 3 | 3 | 1.656 | 0.00908 | 80 | 0.00133 | 7 | 0.01248 | 12 |
| i | 3 | 3 | 1.508 | 0.00923 | 81 | 0.00099 | 6 | 0.01278 | 12 |
| i | 3 | 3 | 1.556 | 0.00766 | 68 | 0.00111 | 8 | 0.01309 | 12 |
| i | 3 | 3 | 1.592 | 0.00693 | 69 | 0.001 | 7 | 0.01326 | 12 |
| i | 4 | 1 | 1.568 | 0.00687 | 58 | 0.00268 | 8 | 0.01068 | 10 |
| i | 4 | 1 | 1.556 | 0.00737 | 59 | 0.00256 | 8 | 0.01215 | 10 |
| i | 4 | 1 | 1.404 | 0.00814 | 66 | 0.00267 | 8 | 0.01295 | 10 |
| i | 4 | 2 | 1.412 | 0.008 | 47 | 0.00213 | 8 | 0.0124 | 10 |
| i | 4 | 2 | 1.332 | 0.00801 | 67 | 0.00159 | 7 | 0.01308 | 10 |
| i | 4 | 2 | 1.342 | 0.00778 | 68 | 0.00307 | 8 | 0.01146 | 10 |
| i | 4 | 2 | 1.368 | 0.00791 | 65 | 0.00233 | 8 | 0.01325 | 10 |
| i | 4 | 3 | 1.456 | 0.00767 | 67 | 0.00199 | 7 | 0.0112 | 10 |
| i | 4 | 3 | 1.374 | 0.0083 | 68 | 0.002 | 8 | 0.01201 | 10 |
| i | 4 | 3 | 1.514 | 0.00787 | 64 | 0.00217 | 8 | 0.01091 | 10 |
| j | 1 | 1 | 1.834 | 0.01053 | 77 | 0.00281 | 8 | 0.01544 | 11 |
| j | 1 | 1 | 1.772 | 0.01104 | 74 | 0.00174 | 8 | 0.01368 | 11 |
| j | 1 | 1 | 1.816 | 0.01064 | 72 | 0.00163 | 8 | 0.01505 | 11 |
| j | 1 | 2 | 1.554 | 0.01214 | 78 | 0.0018 | 7 | 0.01636 | 11 |
| j | 1 | 2 | 1.764 | 0.01103 | 81 | 0.00198 | 8 | 0.01417 | 11 |
| j | 1 | 2 | 1.772 | 0.01098 | 79 | 0.00195 | 7 | 0.01478 | 11 |
| j | 1 | 2 | 1.732 | 0.01127 | 79 | 0.00155 | 8 | 0.01496 | 11 |
| j | 1 | 2 | 1.872 | 0.01057 | 81 | 0.00243 | 8 | 0.01408 | 11 |
| j | 1 | 3 | 1.836 | 0.01103 | 83 | 0.00195 | 8 | 0.01569 | 11 |
| j | 1 | 3 | 1.7 | 0.01069 | 82 | 0.00156 | 7 | 0.01539 | 11 |
| j | 1 | 3 | 1.782 | 0.01093 | 81 | 0.00164 | 6 | 0.01628 | 11 |
| j | 2 | 1 | 1.628 | 0.00681 | 62 | 0.00464 | 13 | 0.01619 | 20 |
| j | 2 | 1 | 1.894 | 0.0079 | 55 | 0.00889 | 13 | 0.01722 | 20 |
| j | 2 | 1 | 1.9 | 0.0075 | 56 | 0.00854 | 14 | 0.0154 | 20 |
| j | 2 | 1 | 1.642 | 0.00631 | 55 | 0.00673 | 13 | 0.01474 | 20 |

| | | | | | | | | | |
|---|---|---|---|---|---|---|---|---|---|
| j | 2 | 1 | 1.786 | 0.00789 | 63 | 0.00561 | 13 | 0.01447 | 20 |
| j | 2 | 1 | 2.074 | 0.00733 | 61 | 0.00605 | 13 | 0.01517 | 20 |
| j | 2 | 2 | 1.99 | 0.00769 | 63 | 0.00501 | 12 | 0.01491 | 20 |
| j | 2 | 2 | 1.69 | 0.00848 | 63 | 0.00504 | 13 | 0.01652 | 20 |
| j | 2 | 2 | 1.898 | 0.00845 | 65 | 0.00525 | 11 | 0.01551 | 20 |
| j | 2 | 2 | 1.814 | 0.00833 | 63 | 0.00364 | 11 | 0.01429 | 20 |
| j | 2 | 2 | 1.808 | 0.0074 | 74 | 0.00474 | 11 | 0.015 | 20 |
| j | 2 | 2 | 1.746 | 0.00867 | 63 | 0.00396 | 10 | 0.0134 | 20 |
| j | 2 | 2 | 1.662 | 0.00882 | 68 | 0.00369 | 10 | 0.01542 | 20 |
| j | 2 | 2 | 1.772 | 0.00844 | 68 | 0.0043 | 10 | 0.01358 | 20 |
| j | 2 | 3 | 1.672 | 0.00847 | 65 | 0.00402 | 9 | 0.01331 | 20 |
| j | 2 | 3 | 1.756 | 0.00842 | 70 | 0.00333 | 8 | 0.01435 | 20 |
| j | 2 | 3 | 1.734 | 0.00532 | 46 | 0.00351 | 7 | 0.01539 | 20 |
| j | 2 | 3 | 1.624 | 0.00814 | 72 | 0.00387 | 8 | 0.01577 | 20 |
| j | 2 | 3 | 1.564 | 0.00836 | 70 | 0.00388 | 8 | 0.0162 | 20 |
| j | 2 | 3 | 1.752 | 0.00918 | 73 | 0.0033 | 9 | 0.01513 | 20 |
| j | 5 | 1 | 1.756 | 0.0089 | 68 | 0.00203 | 8 | 0.01345 | 12 |
| j | 5 | 1 | 1.884 | 0.00873 | 71 | 0.00183 | 8 | 0.01394 | 12 |
| j | 5 | 1 | 1.73 | 0.00949 | 73 | 0.00203 | 8 | 0.01451 | 12 |
| j | 5 | 1 | 1.718 | 0.00995 | 74 | 0.00185 | 8 | 0.01494 | 12 |
| j | 5 | 2 | 1.768 | 0.00947 | 74 | 0.00154 | 8 | 0.01327 | 12 |
| j | 5 | 2 | 1.826 | 0.0097 | 75 | 0.00193 | 8 | 0.0132 | 12 |
| j | 5 | 2 | 1.448 | 0.00914 | 66 | 0.00103 | 7 | 0.01332 | 12 |
| j | 5 | 2 | 1.806 | 0.00752 | 67 | 0.00144 | 8 | 0.01383 | 12 |
| j | 5 | 3 | 1.914 | 0.01024 | 78 | 0.00151 | 7 | 0.01556 | 12 |
| j | 5 | 3 | 1.652 | 0.00879 | 80 | 0.00329 | 8 | 0.01589 | 12 |
| j | 5 | 3 | 2.036 | 0.0087 | 78 | 0.00209 | 8 | 0.01576 | 12 |
| j | 5 | 3 | 1.922 | 0.00885 | 83 | 0.00242 | 9 | 0.01363 | 12 |
| j | 7 | 1 | 1.868 | 0.00476 | 42 | 0.00646 | 11 | 0.01286 | 28 |
| j | 7 | 1 | 1.968 | 0.00625 | 43 | 0.00489 | 11 | 0.01307 | 28 |
| j | 7 | 1 | 1.876 | 0.00532 | 39 | 0.0041 | 12 | 0.01228 | 28 |
| j | 7 | 1 | 1.988 | 0.00716 | 54 | 0.00601 | 13 | 0.01246 | 28 |
| j | 7 | 1 | 1.864 | 0.00686 | 44 | 0.00619 | 8 | 0.01433 | 28 |
| j | 7 | 1 | 1.704 | 0.00694 | 44 | 0.00595 | 9 | 0.0132 | 28 |
| j | 7 | 1 | 2.034 | 0.00593 | 46 | 0.00609 | 10 | 0.01221 | 28 |
| j | 7 | 1 | 1.658 | 0.00797 | 56 | 0.0059 | 9 | 0.01357 | 28 |
| j | 7 | 2 | 1.628 | 0.00788 | 70 | 0.00202 | 5 | 0.0136 | 28 |
| j | 7 | 2 | 2.046 | 0.00558 | 40 | 0.00714 | 14 | 0.01467 | 28 |
| j | 7 | 2 | 1.776 | 0.0048 | 33 | 0.00621 | 9 | 0.01262 | 28 |
| j | 7 | 2 | 1.894 | 0.00725 | 49 | 0.00757 | 14 | 0.01703 | 28 |
| j | 7 | 2 | 1.896 | 0.00769 | 53 | 0.00697 | 13 | 0.01341 | 28 |
| j | 7 | 2 | 1.786 | 0.00782 | 54 | 0.00462 | 12 | 0.01421 | 28 |
| j | 7 | 2 | 1.698 | 0.0076 | 51 | 0.00575 | 13 | 0.0134 | 28 |

| | | | | | | | | | |
|---|---|---|---|---|---|---|---|---|---|
| j | 7 | 2 | 1.406 | 0.00298 | 28 | 0.00634 | 10 | 0.01202 | 28 |
| j | 7 | 2 | 1.864 | 0.00642 | 43 | 0.00705 | 13 | 0.01596 | 28 |
| j | 7 | 2 | 1.854 | 0.00586 | 43 | 0.00457 | 8 | 0.01234 | 28 |
| j | 7 | 3 | 1.86 | 0.00738 | 51 | 0.00568 | 10 | 0.01743 | 28 |
| j | 7 | 3 | 1.864 | 0.00799 | 61 | 0.00363 | 8 | 0.01495 | 28 |
| j | 7 | 3 | 2.072 | 0.00831 | 55 | 0.0054 | 11 | 0.01644 | 28 |
| j | 7 | 3 | 1.888 | 0.00911 | 65 | 0.00647 | 10 | 0.01514 | 28 |
| j | 7 | 3 | 1.928 | 0.0073 | 49 | 0.00509 | 7 | 0.01835 | 28 |
| j | 7 | 3 | 1.97 | 0.00758 | 58 | 0.00519 | 8 | 0.01921 | 28 |
| j | 7 | 3 | 1.692 | 0.00915 | 69 | 0.00503 | 8 | 0.01867 | 28 |
| j | 7 | 3 | 1.692 | 0.00791 | 57 | 0.00446 | 8 | 0.01805 | 28 |
| j | 7 | 3 | 1.782 | 0.01046 | 73 | 0.00624 | 13 | 0.01626 | 28 |
| k | 2 | 1 | 1.854 | 0.00827 | 61 | 0.0062 | 15 | 0.01588 | 15 |
| k | 2 | 1 | 1.932 | 0.00856 | 62 | 0.00618 | 15 | 0.01515 | 15 |
| k | 2 | 1 | 1.692 | 0.00829 | 59 | 0.0056 | 15 | 0.01566 | 15 |
| k | 2 | 1 | 1.752 | 0.00951 | 67 | 0.00539 | 11 | 0.01647 | 15 |
| k | 2 | 1 | 1.866 | 0.00969 | 65 | 0.00758 | 13 | 0.01741 | 15 |
| k | 2 | 2 | 1.766 | 0.01024 | 70 | 0.00453 | 12 | 0.01598 | 15 |
| k | 2 | 2 | 1.758 | 0.01036 | 71 | 0.00523 | 13 | 0.01819 | 15 |
| k | 2 | 2 | 1.852 | 0.00984 | 73 | 0.00456 | 9 | 0.01443 | 15 |
| k | 2 | 2 | 1.988 | 0.0107 | 72 | 0.00425 | 10 | 0.01524 | 15 |
| k | 2 | 2 | 1.618 | 0.01009 | 72 | 0.00454 | 9 | 0.01388 | 15 |
| k | 2 | 3 | 1.944 | 0.00991 | 70 | 0.00455 | 9 | 0.01475 | 15 |
| k | 2 | 3 | 1.652 | 0.01029 | 75 | 0.00375 | 10 | 0.01459 | 15 |
| k | 2 | 3 | 1.692 | 0.00969 | 76 | 0.0038 | 8 | 0.01367 | 15 |
| k | 2 | 3 | 1.534 | 0.00916 | 72 | 0.00375 | 8 | 0.01342 | 15 |
| k | 2 | 3 | 1.688 | 0.00856 | 68 | 0.00309 | 8 | 0.01305 | 15 |
| k | 3 | 1 | 1.816 | 0.00918 | 66 | 0.00495 | 13 | 0.01473 | 17 |
| k | 3 | 1 | 1.82 | 0.00846 | 62 | 0.00584 | 12 | 0.01531 | 17 |
| k | 3 | 1 | 1.726 | 0.00995 | 65 | 0.00655 | 13 | 0.01754 | 17 |
| k | 3 | 1 | 1.824 | 0.01119 | 72 | 0.00509 | 10 | 0.01813 | 17 |
| k | 3 | 1 | 1.954 | 0.00854 | 61 | 0.01133 | 13 | 0.01686 | 17 |
| k | 3 | 2 | 1.992 | 0.01247 | 77 | 0.00825 | 12 | 0.01934 | 17 |
| k | 3 | 2 | 1.94 | 0.00843 | 67 | 0.00696 | 12 | 0.01424 | 17 |
| k | 3 | 2 | 1.906 | 0.0079 | 69 | 0.00597 | 12 | 0.01461 | 17 |
| k | 3 | 2 | 1.708 | 0.00904 | 76 | 0.00301 | 8 | 0.01487 | 17 |
| k | 3 | 2 | 1.864 | 0.00866 | 65 | 0.00339 | 9 | 0.01584 | 17 |
| k | 3 | 2 | 1.894 | 0.00784 | 69 | 0.00376 | 8 | 0.01496 | 17 |
| k | 3 | 2 | 1.784 | 0.00864 | 72 | 0.0039 | 8 | 0.01928 | 17 |
| k | 3 | 3 | 1.87 | 0.00819 | 65 | 0.00304 | 8 | 0.01572 | 17 |
| k | 3 | 3 | 1.712 | 0.00883 | 69 | 0.00303 | 9 | 0.01764 | 17 |
| k | 3 | 3 | 1.878 | 0.00874 | 64 | 0.003 | 8 | 0.01742 | 17 |
| k | 3 | 3 | 1.728 | 0.00811 | 68 | 0.00296 | 8 | 0.01768 | 17 |

| | | | | | | | | | |
|---|---|---|---|---|---|---|---|---|---|
| k | 3 | 3 | 1.71 | 0.00532 | 54 | 0.00271 | 8 | 0.01364 | 17 |
| k | 6 | 1 | 2.062 | 0.01128 | 74 | 0.00731 | 11 | 0.01934 | 27 |
| k | 6 | 1 | 1.846 | 0.01047 | 69 | 0.00598 | 10 | 0.01828 | 27 |
| k | 6 | 1 | 1.868 | 0.01028 | 71 | 0.00533 | 11 | 0.01744 | 27 |
| k | 6 | 1 | 1.962 | 0.01119 | 76 | 0.00801 | 13 | 0.01878 | 27 |
| k | 6 | 1 | 1.854 | 0.00932 | 65 | 0.00455 | 11 | 0.01617 | 27 |
| k | 6 | 1 | 1.922 | 0.00627 | 44 | 0.00434 | 5 | 0.01541 | 27 |
| k | 6 | 1 | 1.786 | 0.00985 | 71 | 0.00528 | 8 | 0.01686 | 27 |
| k | 6 | 1 | 1.784 | 0.00657 | 46 | 0.00203 | 6 | 0.01519 | 27 |
| k | 6 | 1 | 1.754 | 0.01058 | 69 | 0.00316 | 9 | 0.02084 | 27 |
| k | 6 | 2 | 1.844 | 0.01117 | 64 | 0.00487 | 11 | 0.02043 | 27 |
| k | 6 | 2 | 1.914 | 0.01073 | 69 | 0.00401 | 10 | 0.01917 | 27 |
| k | 6 | 2 | 1.782 | 0.01023 | 75 | 0.00191 | 8 | 0.01806 | 27 |
| k | 6 | 2 | 1.87 | 0.01045 | 74 | 0.00341 | 8 | 0.01861 | 27 |
| k | 6 | 2 | 1.894 | 0.00911 | 74 | 0.00282 | 8 | 0.01616 | 27 |
| k | 6 | 2 | 1.986 | 0.00901 | 65 | 0.0081 | 10 | 0.01701 | 27 |
| k | 6 | 2 | 1.836 | 0.00862 | 72 | 0.00425 | 9 | 0.01477 | 27 |
| k | 6 | 2 | 1.922 | 0.00578 | 45 | 0.00195 | 5 | 0.01557 | 27 |
| k | 6 | 2 | 1.712 | 0.0111 | 69 | 0.00329 | 9 | 0.02509 | 27 |
| k | 6 | 3 | 1.802 | 0.00737 | 72 | 0.00396 | 10 | 0.01512 | 27 |
| k | 6 | 3 | 1.932 | 0.01001 | 65 | 0.00277 | 8 | 0.01775 | 27 |
| k | 6 | 3 | 1.984 | 0.00993 | 72 | 0.00284 | 8 | 0.01866 | 27 |
| k | 6 | 3 | 1.652 | 0.01108 | 73 | 0.00392 | 9 | 0.01964 | 27 |
| k | 6 | 3 | 1.756 | 0.00645 | 60 | 0.00219 | 9 | 0.01554 | 27 |
| k | 6 | 3 | 1.882 | 0.01032 | 79 | 0.00407 | 9 | 0.01797 | 27 |
| k | 6 | 3 | 1.87 | 0.01075 | 74 | 0.00281 | 8 | 0.01906 | 27 |
| k | 6 | 3 | 1.938 | 0.01085 | 71 | 0.00548 | 11 | 0.01753 | 27 |
| k | 6 | 3 | 1.93 | 0.01069 | 66 | 0.0027 | 8 | 0.01924 | 27 |
| k | 7 | 1 | 1.974 | 0.00415 | 28 | 0.00568 | 8 | 0.01112 | 28 |
| k | 7 | 1 | 1.792 | 0.00532 | 52 | 0.00211 | 8 | 0.00992 | 10 |
| k | 7 | 1 | 1.784 | 0.00553 | 49 | 0.00133 | 8 | 0.00987 | 10 |
| k | 7 | 1 | 1.694 | 0.00499 | 55 | 0.00143 | 9 | 0.0102 | 10 |
| k | 7 | 2 | 1.744 | 0.00509 | 48 | 0.00116 | 8 | 0.00937 | 10 |
| k | 7 | 2 | 1.742 | 0.00373 | 48 | 0.00102 | 6 | 0.00931 | 10 |
| k | 7 | 2 | 1.468 | 0.00571 | 51 | 0.00112 | 8 | 0.00867 | 10 |
| k | 7 | 2 | 1.678 | 0.00373 | 50 | 0.00122 | 7 | 0.00931 | 10 |
| k | 7 | 3 | 1.464 | 0.00516 | 48 | 0.00139 | 8 | 0.01019 | 10 |
| k | 7 | 3 | 1.786 | 0.00503 | 53 | 0.0011 | 8 | 0.00928 | 10 |
| k | 7 | 3 | 1.702 | 0.00509 | 55 | 0.00096 | 7 | 0.00993 | 10 |
| l | 1 | 1 | 1.928 | 0.01309 | 95 | 0.0032 | 10 | 0.01632 | 27 |
| l | 1 | 1 | 1.968 | 0.01323 | 100 | 0.00313 | 12 | 0.01793 | 27 |
| l | 1 | 1 | 1.968 | 0.01268 | 98 | 0.00243 | 12 | 0.01557 | 27 |
| l | 1 | 1 | 1.972 | 0.00935 | 77 | 0.00341 | 13 | 0.01413 | 27 |

| | | | | | | | | | |
|---|---|---|---|---|---|---|---|---|---|
| l | 1 | 1 | 2.056 | 0.01165 | 91 | 0.00259 | 11 | 0.0143 | 27 |
| l | 1 | 1 | 1.994 | 0.01076 | 85 | 0.00312 | 12 | 0.01654 | 27 |
| l | 1 | 1 | 1.914 | 0.01097 | 78 | 0.00244 | 12 | 0.01471 | 27 |
| l | 1 | 1 | 1.924 | 0.01095 | 81 | 0.00133 | 9 | 0.0148 | 27 |
| l | 1 | 1 | 1.982 | 0.01189 | 100 | 0.00215 | 12 | 0.01727 | 27 |
| l | 1 | 2 | 1.914 | 0.01111 | 84 | 0.00225 | 8 | 0.01552 | 27 |
| l | 1 | 2 | 1.868 | 0.00996 | 70 | 0.00243 | 8 | 0.01546 | 27 |
| l | 1 | 2 | 1.902 | 0.01099 | 85 | 0.00261 | 11 | 0.01477 | 27 |
| l | 1 | 2 | 1.886 | 0.01241 | 95 | 0.00164 | 8 | 0.01521 | 27 |
| l | 1 | 2 | 1.9 | 0.01255 | 101 | 0.00243 | 10 | 0.01624 | 27 |
| l | 1 | 2 | 1.952 | 0.0119 | 87 | 0.00157 | 7 | 0.01801 | 27 |
| l | 1 | 2 | 1.692 | 0.00998 | 85 | 0.00164 | 7 | 0.01722 | 27 |
| l | 1 | 2 | 1.848 | 0.00676 | 75 | 0.00231 | 9 | 0.0148 | 27 |
| l | 1 | 2 | 1.954 | 0.01228 | 82 | 0.00158 | 8 | 0.01795 | 27 |
| l | 1 | 3 | 1.826 | 0.01308 | 98 | 0.00295 | 8 | 0.0178 | 27 |
| l | 1 | 3 | 2.006 | 0.01062 | 94 | 0.00128 | 8 | 0.01386 | 27 |
| l | 1 | 3 | 1.932 | 0.00969 | 75 | 0.00168 | 8 | 0.01597 | 27 |
| l | 1 | 3 | 1.888 | 0.01173 | 91 | 0.00192 | 8 | 0.0165 | 27 |
| l | 1 | 3 | 2.024 | 0.01127 | 88 | 0.00196 | 8 | 0.01614 | 27 |
| l | 1 | 3 | 1.918 | 0.01037 | 95 | 0.00197 | 8 | 0.01597 | 27 |
| l | 1 | 3 | 2.058 | 0.01178 | 93 | 0.00261 | 7 | 0.01824 | 27 |
| l | 1 | 3 | 2.086 | 0.01063 | 81 | 0.00172 | 8 | 0.016 | 27 |
| l | 1 | 3 | 1.978 | 0.01255 | 91 | 0.00173 | 8 | 0.019 | 27 |
| l | 3 | 1 | 1.862 | 0.00957 | 70 | 0.00211 | 8 | 0.01403 | 11 |
| l | 3 | 1 | 1.972 | 0.0084 | 68 | 0.0015 | 7 | 0.01304 | 11 |
| l | 3 | 1 | 1.942 | 0.00882 | 72 | 0.00154 | 7 | 0.01376 | 11 |
| l | 3 | 2 | 1.792 | 0.00875 | 75 | 0.00185 | 6 | 0.01494 | 11 |
| l | 3 | 2 | 1.632 | 0.00977 | 77 | 0.00126 | 6 | 0.01547 | 11 |
| l | 3 | 2 | 1.7 | 0.00869 | 76 | 0.00131 | 6 | 0.01425 | 11 |
| l | 3 | 2 | 1.518 | 0.00922 | 77 | 0.00107 | 6 | 0.0151 | 11 |
| l | 3 | 2 | 1.52 | 0.00894 | 79 | 0.00087 | 5 | 0.01148 | 11 |
| l | 3 | 3 | 1.852 | 0.00866 | 73 | 0.00103 | 6 | 0.01567 | 11 |
| l | 3 | 3 | 1.62 | 0.00889 | 76 | 0.00126 | 7 | 0.01579 | 11 |
| l | 3 | 3 | 1.548 | 0.00883 | 75 | 0.00128 | 5 | 0.01555 | 11 |
| l | 4 | 1 | 1.766 | 0.00812 | 59 | 0.0032 | 11 | 0.01406 | 9 |
| l | 4 | 1 | 1.784 | 0.00821 | 58 | 0.00323 | 12 | 0.01436 | 9 |
| l | 4 | 1 | 1.736 | 0.00805 | 60 | 0.00354 | 12 | 0.01562 | 9 |
| l | 4 | 2 | 1.632 | 0.00814 | 56 | 0.00295 | 11 | 0.01371 | 9 |
| l | 4 | 2 | 1.646 | 0.00561 | 63 | 0.00097 | 6 | 0.00918 | 9 |
| l | 4 | 2 | 1.888 | 0.00535 | 54 | 0.00127 | 6 | 0.00907 | 9 |
| l | 4 | 3 | 1.552 | 0.00621 | 62 | 0.00323 | 12 | 0.00135 | 9 |
| l | 4 | 3 | 1.676 | 0.00766 | 74 | 0.00594 | 12 | 0.01463 | 9 |
| l | 4 | 3 | 1.742 | 0.00752 | 72 | 0.00424 | 10 | 0.01523 | 9 |

| | | | | | | | | | |
|---|---|---|---|---|---|---|---|---|---|
| l | 5 | 1 | 1.888 | 0.00576 | 56 | 0.00201 | 8 | 0.00984 | 10 |
| l | 5 | 1 | 1.768 | 0.00558 | 58 | 0.0016 | 8 | 0.01105 | 10 |
| l | 5 | 1 | 1.775 | 0.0056 | 59 | 0.00174 | 8 | 0.01232 | 10 |
| l | 5 | 1 | 1.743 | 0.00542 | 55 | 0.00156 | 8 | 0.0101 | 10 |
| l | 5 | 2 | 1.608 | 0.00663 | 57 | 0.00146 | 8 | 0.01098 | 10 |
| l | 5 | 2 | 1.746 | 0.00555 | 54 | 0.00156 | 7 | 0.01111 | 10 |
| l | 5 | 2 | 1.522 | 0.00588 | 58 | 0.00113 | 7 | 0.01139 | 10 |
| l | 5 | 3 | 1.678 | 0.00542 | 52 | 0.00127 | 8 | 0.01149 | 10 |
| l | 5 | 3 | 1.752 | 0.00564 | 54 | 0.00119 | 8 | 0.01098 | 10 |
| l | 5 | 3 | 1.746 | 0.00537 | 55 | 0.00102 | 6 | 0.01091 | 10 |
| m | 1 | 1 | 1.644 | 0.00666 | 63 | 0.00182 | 8 | 0.01157 | 9 |
| m | 1 | 1 | 1.648 | 0.006 | 59 | 0.00112 | 8 | 0.0105 | 9 |
| m | 1 | 1 | 1.526 | 0.00751 | 53 | 0.001 | 8 | 0.01079 | 9 |
| m | 1 | 2 | 1.796 | 0.00686 | 60 | 0.00164 | 8 | 0.01066 | 9 |
| m | 1 | 2 | 1.704 | 0.00655 | 62 | 0.00155 | 8 | 0.0101 | 9 |
| m | 1 | 2 | 1.686 | 0.00714 | 59 | 0.00129 | 8 | 0.0099 | 9 |
| m | 1 | 3 | 1.714 | 0.00728 | 55 | 0.00136 | 7 | 0.01007 | 9 |
| m | 1 | 3 | 1.706 | 0.00706 | 67 | 0.00138 | 6 | 0.01042 | 9 |
| m | 1 | 3 | 1.732 | 0.00711 | 64 | 0.00132 | 7 | 0.01024 | 9 |
| m | 2 | 1 | 1.804 | 0.01016 | 75 | 0.00654 | 11 | 0.0166 | 10 |
| m | 2 | 1 | 1.718 | 0.01043 | 75 | 0.00669 | 13 | 0.01564 | 10 |
| m | 2 | 1 | 1.702 | 0.00946 | 74 | 0.00643 | 13 | 0.0163 | 10 |
| m | 2 | 1 | 1.689 | 0.0101 | 76 | 0.00575 | 12 | 0.01612 | 10 |
| m | 2 | 2 | 1.561 | 0.00932 | 72 | 0.00401 | 9 | 0.01637 | 10 |
| m | 2 | 2 | 1.574 | 0.00862 | 68 | 0.00376 | 8 | 0.01632 | 10 |
| m | 2 | 2 | 1.678 | 0.00988 | 75 | 0.00616 | 12 | 0.0167 | 10 |
| m | 2 | 3 | 1.704 | 0.0104 | 79 | 0.00537 | 11 | 0.01661 | 10 |
| m | 2 | 3 | 1.554 | 0.00913 | 71 | 0.00414 | 9 | 0.0161 | 10 |
| m | 2 | 3 | 1.554 | 0.00782 | 66 | 0.0034 | 8 | 0.01614 | 10 |
| m | 3 | 1 | 1.56 | 0.00844 | 63 | 0.00402 | 8 | 0.01324 | 9 |
| m | 3 | 1 | 1.632 | 0.01056 | 71 | 0.00259 | 8 | 0.01496 | 9 |
| m | 3 | 1 | 1.687 | 0.01135 | 70 | 0.00284 | 8 | 0.01512 | 9 |
| m | 3 | 1 | 1.655 | 0.01079 | 67 | 0.00266 | 8 | 0.01505 | 9 |
| m | 3 | 2 | 1.704 | 0.01027 | 77 | 0.00284 | 8 | 0.01509 | 9 |
| m | 3 | 2 | 1.754 | 0.00983 | 79 | 0.00253 | 8 | 0.0121 | 9 |
| m | 3 | 2 | 1.702 | 0.01038 | 76 | 0.00363 | 8 | 0.01345 | 9 |
| m | 3 | 3 | 1.756 | 0.01021 | 77 | 0.00283 | 8 | 0.0137 | 9 |
| m | 3 | 3 | 1.71 | 0.01026 | 77 | 0.00436 | 8 | 0.01587 | 9 |
| m | 4 | 1 | 1.748 | 0.01201 | 72 | 0.0047 | 13 | 0.01932 | 14 |
| m | 4 | 1 | 1.77 | 0.01242 | 73 | 0.00457 | 13 | 0.01963 | 14 |
| m | 4 | 1 | 1.72 | 0.01162 | 74 | 0.00472 | 12 | 0.01873 | 14 |
| m | 4 | 1 | 1.72 | 0.01268 | 76 | 0.0048 | 12 | 0.01738 | 14 |
| m | 4 | 2 | 1.714 | 0.0113 | 70 | 0.00399 | 10 | 0.01811 | 14 |

| | | | | | | | | | |
|---|---|---|---|---|---|---|---|---|---|
| m | 4 | 2 | 1.604 | 0.0115 | 77 | 0.0064 | 13 | 0.01818 | 14 |
| m | 4 | 2 | 1.66 | 0.00943 | 76 | 0.00645 | 11 | 0.01677 | 14 |
| m | 4 | 2 | 1.608 | 0.01108 | 78 | 0.00504 | 11 | 0.01584 | 14 |
| m | 4 | 2 | 1.64 | 0.01103 | 76 | 0.00422 | 12 | 0.01639 | 14 |
| m | 4 | 2 | 1.58 | 0.01165 | 77 | 0.00629 | 12 | 0.01692 | 14 |
| m | 4 | 3 | 1.648 | 0.01146 | 76 | 0.00602 | 12 | 0.01736 | 14 |
| m | 4 | 3 | 1.676 | 0.01154 | 77 | 0.00324 | 9 | 0.01631 | 14 |
| m | 4 | 3 | 1.586 | 0.01163 | 79 | 0.00573 | 12 | 0.01508 | 14 |
| m | 4 | 3 | 1.72 | 0.01233 | 71 | 0.00613 | 10 | 0.01817 | 14 |
| n | 1 | 1 | 1.758 | 0.00752 | 64 | 0.00522 | 13 | 0.01284 | 18 |
| n | 1 | 1 | 1.764 | 0.00709 | 60 | 0.00376 | 11 | 0.01381 | 18 |
| n | 1 | 1 | 1.782 | 0.00654 | 54 | 0.00403 | 10 | 0.01253 | 18 |
| n | 1 | 1 | 1.702 | 0.00566 | 46 | 0.00198 | 8 | 0.01062 | 18 |
| n | 1 | 1 | 1.708 | 0.00816 | 73 | 0.00574 | 14 | 0.01192 | 18 |
| n | 1 | 1 | 1.594 | 0.0066 | 58 | 0.00208 | 8 | 0.01181 | 18 |
| n | 1 | 2 | 1.594 | 0.00757 | 51 | 0.00092 | 7 | 0.01134 | 18 |
| n | 1 | 2 | 1.594 | 0.00726 | 53 | 0.00105 | 8 | 0.01117 | 18 |
| n | 1 | 2 | 1.672 | 0.00668 | 61 | 0.00077 | 6 | 0.01268 | 18 |
| n | 1 | 2 | 1.394 | 0.0063 | 54 | 0.00139 | 8 | 0.01235 | 18 |
| n | 1 | 2 | 1.496 | 0.00649 | 66 | 0.00153 | 8 | 0.01216 | 18 |
| n | 1 | 2 | 1.616 | 0.00584 | 48 | 0.0009 | 7 | 0.01245 | 18 |
| n | 1 | 3 | 1.592 | 0.00953 | 55 | 0.00143 | 8 | 0.01186 | 18 |
| n | 1 | 3 | 1.664 | 0.0068 | 49 | 0.00104 | 6 | 0.01106 | 18 |
| n | 1 | 3 | 1.594 | 0.00678 | 65 | 0.00144 | 7 | 0.01176 | 18 |
| n | 1 | 3 | 1.56 | 0.00712 | 62 | 0.00107 | 8 | 0.01213 | 18 |
| n | 1 | 3 | 1.586 | 0.00632 | 57 | 0.00119 | 6 | 0.01067 | 18 |
| n | 1 | 3 | 1.428 | 0.00679 | 47 | 0.00085 | 8 | 0.01056 | 18 |
| n | 2 | 1 | 1.746 | 0.00528 | 50 | 0.00146 | 6 | 0.01023 | 17 |
| n | 2 | 1 | 1.76 | 0.00604 | 50 | 0.00133 | 8 | 0.01084 | 17 |
| n | 2 | 1 | 1.646 | 0.00847 | 62 | 0.0023 | 8 | 0.01321 | 17 |
| n | 2 | 1 | 1.648 | 0.00627 | 57 | 0.00111 | 8 | 0.01163 | 17 |
| n | 2 | 1 | 1.702 | 0.00667 | 50 | 0.00092 | 5 | 0.01112 | 17 |
| n | 2 | 2 | 1.648 | 0.00667 | 50 | 0.00139 | 8 | 0.01292 | 17 |
| n | 2 | 2 | 1.74 | 0.00565 | 47 | 0.00096 | 6 | 0.0108 | 17 |
| n | 2 | 2 | 1.786 | 0.0056 | 54 | 0.00134 | 7 | 0.01127 | 17 |
| n | 2 | 2 | 1.864 | 0.00607 | 71 | 0.00129 | 8 | 0.01203 | 17 |
| n | 2 | 2 | 1.64 | 0.00627 | 58 | 0.00072 | 5 | 0.01213 | 17 |
| n | 2 | 2 | 1.71 | 0.00606 | 57 | 0.00091 | 5 | 0.01121 | 17 |
| n | 2 | 2 | 1.708 | 0.00619 | 57 | 0.00089 | 5 | 0.01157 | 17 |
| n | 2 | 3 | 1.738 | 0.00699 | 57 | 0.00159 | 7 | 0.01185 | 17 |
| n | 2 | 3 | 1.658 | 0.00575 | 56 | 0.00127 | 5 | 0.01132 | 17 |
| n | 2 | 3 | 1.864 | 0.00609 | 52 | 0.00068 | 5 | 0.01198 | 17 |
| n | 2 | 3 | 1.652 | 0.00597 | 57 | 0.00104 | 6 | 0.0123 | 17 |

| | | | | | | | | | |
|---|---|---|---|---|---|---|---|---|---|
| n | 2 | 3 | 1.808 | 0.00576 | 55 | 0.00114 | 5 | 0.01174 | 17 |
| n | 3 | 1 | 1.746 | 0.00776 | 58 | 0.00256 | 8 | 0.01435 | 9 |
| n | 3 | 1 | 1.786 | 0.00734 | 55 | 0.00511 | 9 | 0.01312 | 9 |
| n | 3 | 1 | 1.674 | 0.00735 | 58 | 0.002666 | 8 | 0.01291 | 9 |
| n | 3 | 2 | 1.51 | 0.00726 | 53 | 0.00192 | 8 | 0.01298 | 9 |
| n | 3 | 2 | 1.694 | 0.00777 | 49 | 0.00236 | 8 | 0.01545 | 9 |
| n | 3 | 2 | 1.556 | 0.00681 | 50 | 0.00171 | 7 | 0.0152 | 9 |
| n | 3 | 3 | 1.614 | 0.00459 | 33 | 0.00159 | 5 | 0.01009 | 9 |
| n | 3 | 3 | 1.578 | 0.00744 | 58 | 0.00315 | 8 | 0.01632 | 9 |
| n | 3 | 3 | 1.596 | 0.00709 | 58 | 0.00273 | 8 | 0.01148 | 9 |
| n | 4 | 1 | 1.516 | 0.0081 | 68 | 0.00242 | 7 | 0.01186 | 10 |
| n | 4 | 1 | 1.654 | 0.00818 | 66 | 0.0021 | 7 | 0.0134 | 10 |
| n | 4 | 1 | 1.846 | 0.00605 | 50 | 0.00182 | 6 | 0.01358 | 10 |
| n | 4 | 1 | 1.732 | 0.00665 | 52 | 0.00179 | 6 | 0.01287 | 10 |
| n | 4 | 2 | 1.618 | 0.00749 | 66 | 0.00151 | 6 | 0.01181 | 10 |
| n | 4 | 2 | 1.676 | 0.00839 | 67 | 0.00185 | 7 | 0.01264 | 10 |
| n | 4 | 2 | 1.728 | 0.00576 | 48 | 0.00146 | 5 | 0.01477 | 10 |
| n | 4 | 3 | 1.766 | 0.00637 | 53 | 0.00164 | 5 | 0.01344 | 10 |
| n | 4 | 3 | 1.614 | 0.00718 | 59 | 0.00105 | 5 | 0.01421 | 10 |
| n | 4 | 3 | 1.792 | 0.00766 | 62 | 0.00195 | 7 | 0.01263 | 10 |
| o | 1 | 1 | 1.85 | 0.01011 | 70 | 0.00761 | 17 | 0.01732 | 18 |
| o | 1 | 1 | 1.702 | 0.01036 | 72 | 0.00782 | 16 | 0.01762 | 18 |
| o | 1 | 1 | 1.792 | 0.00973 | 69 | 0.00661 | 16 | 0.01626 | 18 |
| o | 1 | 1 | 1.696 | 0.0098 | 73 | 0.00681 | 16 | 0.01537 | 18 |
| o | 1 | 1 | 1.778 | 0.01001 | 72 | 0.00927 | 15 | 0.01618 | 18 |
| o | 1 | 1 | 1.694 | 0.0099 | 70 | 0.0066 | 14 | 0.01561 | 18 |
| o | 1 | 2 | 1.682 | 0.01003 | 75 | 0.006 | 13 | 0.01559 | 18 |
| o | 1 | 2 | 1.632 | 0.0095 | 72 | 0.00534 | 14 | 0.01334 | 18 |
| o | 1 | 2 | 1.656 | 0.01003 | 70 | 0.00771 | 15 | 0.01517 | 18 |
| o | 1 | 2 | 1.642 | 0.01048 | 75 | 0.00658 | 15 | 0.01495 | 18 |
| o | 1 | 2 | 1.762 | 0.00958 | 76 | 0.00377 | 12 | 0.01459 | 18 |
| o | 1 | 2 | 1.592 | 0.00911 | 73 | 0.00683 | 15 | 0.01322 | 18 |
| o | 1 | 3 | 1.692 | 0.00915 | 77 | 0.00603 | 11 | 0.01317 | 18 |
| o | 1 | 3 | 1.762 | 0.0093 | 74 | 0.00602 | 13 | 0.01343 | 18 |
| o | 1 | 3 | 1.698 | 0.00907 | 76 | 0.0058 | 14 | 0.01364 | 18 |
| o | 1 | 3 | 1.7 | 0.00921 | 77 | 0.00685 | 13 | 0.0137 | 18 |
| o | 1 | 3 | 1.64 | 0.00903 | 78 | 0.00549 | 12 | 0.01352 | 18 |
| o | 1 | 3 | 1.682 | 0.00694 | 69 | 0.00624 | 12 | 0.01378 | 18 |
| o | 3 | 1 | 1.844 | 0.00949 | 63 | 0.01346 | 15 | 0.01702 | 21 |
| o | 3 | 1 | 1.53 | 0.00712 | 60 | 0.00218 | 8 | 0.01659 | 21 |
| o | 3 | 1 | 1.804 | 0.00922 | 71 | 0.00781 | 13 | 0.01683 | 21 |
| o | 3 | 1 | 1.734 | 0.00798 | 61 | 0.00187 | 7 | 0.01527 | 21 |
| o | 3 | 1 | 1.712 | 0.00972 | 63 | 0.0101 | 16 | 0.01685 | 21 |

| | | | | | | | | | |
|---|---|---|---|---|---|---|---|---|---|
| o | 3 | 1 | 1.894 | 0.01115 | 74 | 0.01203 | 15 | 0.01826 | 21 |
| o | 3 | 1 | 1.562 | 0.00835 | 63 | 0.00191 | 8 | 0.01585 | 21 |
| o | 3 | 2 | 1.744 | 0.00807 | 55 | 0.00162 | 8 | 0.01553 | 21 |
| o | 3 | 2 | 1.74 | 0.00994 | 70 | 0.00502 | 11 | 0.01597 | 21 |
| o | 3 | 2 | 1.672 | 0.01017 | 71 | 0.00697 | 15 | 0.01616 | 21 |
| o | 3 | 2 | 1.706 | 0.01005 | 77 | 0.00791 | 13 | 0.0169 | 21 |
| o | 3 | 2 | 1.804 | 0.01089 | 71 | 0.00771 | 13 | 0.0184 | 21 |
| o | 3 | 2 | 1.512 | 0.00791 | 57 | 0.00205 | 9 | 0.01465 | 21 |
| o | 3 | 2 | 1.768 | 0.01029 | 68 | 0.00515 | 13 | 0.018 | 21 |
| o | 3 | 3 | 1.784 | 0.01009 | 67 | 0.00571 | 13 | 0.01862 | 21 |
| o | 3 | 3 | 1.732 | 0.01042 | 70 | 0.00761 | 14 | 0.01841 | 21 |
| o | 3 | 3 | 1.858 | 0.01129 | 74 | 0.00563 | 9 | 0.01751 | 21 |
| o | 3 | 3 | 1.866 | 0.0112 | 74 | 0.00699 | 12 | 0.01806 | 21 |
| o | 3 | 3 | 1.598 | 0.01089 | 75 | 0.00515 | 11 | 0.01777 | 21 |
| o | 3 | 3 | 1.788 | 0.01172 | 75 | 0.00616 | 10 | 0.01729 | 21 |
| o | 3 | 3 | 1.852 | 0.00959 | 72 | 0.00642 | 10 | 0.01724 | 21 |
| o | 4 | 1 | 2.01 | 0.01102 | 76 | 0.00551 | 13 | 0.01809 | 11 |
| o | 4 | 1 | 1.818 | 0.01065 | 77 | 0.00512 | 12 | 0.01656 | 11 |
| o | 4 | 1 | 1.968 | 0.01036 | 74 | 0.00696 | 13 | 0.0179 | 11 |
| o | 4 | 2 | 1.91 | 0.01133 | 77 | 0.00591 | 11 | 0.01812 | 11 |
| o | 4 | 2 | 1.928 | 0.00956 | 70 | 0.00532 | 13 | 0.01674 | 11 |
| o | 4 | 2 | 1.906 | 0.01016 | 71 | 0.00589 | 13 | 0.01409 | 11 |
| o | 4 | 2 | 1.856 | 0.01067 | 78 | 0.00545 | 10 | 0.01551 | 11 |
| o | 4 | 2 | 1.808 | 0.0104 | 79 | 0.00472 | 10 | 0.01475 | 11 |
| o | 4 | 3 | 1.83 | 0.00885 | 73 | 0.00481 | 9 | 0.01404 | 11 |
| o | 4 | 3 | 1.716 | 0.00959 | 75 | 0.00446 | 10 | 0.01433 | 11 |
| o | 4 | 3 | 1.862 | 0.00931 | 73 | 0.00466 | 8 | 0.01463 | 11 |
| o | 5 | 1 | 1.714 | 0.00892 | 71 | 0.0067 | 13 | 0.0169 | 21 |
| o | 5 | 1 | 1.75 | 0.0096 | 73 | 0.0036 | 12 | 0.01722 | 21 |
| o | 5 | 1 | 1.916 | 0.00958 | 69 | 0.00561 | 12 | 0.01721 | 21 |
| o | 5 | 1 | 1.928 | 0.00934 | 71 | 0.00556 | 14 | 0.01261 | 21 |
| o | 5 | 1 | 1.856 | 0.00964 | 63 | 0.00686 | 11 | 0.01559 | 21 |
| o | 5 | 1 | 1.944 | 0.00961 | 71 | 0.00481 | 14 | 0.01508 | 21 |
| o | 5 | 1 | 1.708 | 0.00947 | 74 | 0.0045 | 13 | 0.01469 | 21 |
| o | 5 | 2 | 1.854 | 0.00922 | 67 | 0.00555 | 14 | 0.01484 | 21 |
| o | 5 | 2 | 1.914 | 0.00893 | 70 | 0.00426 | 13 | 0.01646 | 21 |
| o | 5 | 2 | 1.756 | 0.00956 | 58 | 0.00268 | 11 | 0.01606 | 21 |
| o | 5 | 2 | 1.924 | 0.00814 | 71 | 0.00464 | 13 | 0.01709 | 21 |
| o | 5 | 2 | 1.678 | 0.01069 | 76 | 0.00635 | 13 | 0.01547 | 21 |
| o | 5 | 2 | 1.864 | 0.01066 | 76 | 0.00423 | 10 | 0.01561 | 21 |
| o | 5 | 2 | 1.866 | 0.00923 | 76 | 0.00458 | 12 | 0.0138 | 21 |
| o | 5 | 3 | 1.804 | 0.00846 | 70 | 0.00536 | 10 | 0.01638 | 21 |
| o | 5 | 3 | 1.748 | 0.00729 | 68 | 0.00444 | 13 | 0.0122 | 21 |

| | | | | | | | | | |
|---|---|---|---|---|---|---|---|---|---|
| o | 5 | 3 | 1.88 | 0.00835 | 73 | 0.00436 | 11 | 0.01205 | 21 |
| o | 5 | 3 | 1.806 | 0.00959 | 74 | 0.00468 | 10 | 0.01349 | 21 |
| o | 5 | 3 | 1.762 | 0.00768 | 69 | 0.00489 | 9 | 0.01348 | 21 |
| o | 5 | 3 | 1.46 | 0.00943 | 72 | 0.00564 | 11 | 0.01505 | 21 |
| o | 5 | 3 | 1.678 | 0.00836 | 72 | 0.00428 | 9 | 0.01531 | 21 |
| p | 1 | 1 | 1.654 | 0.00827 | 71 | 0.00248 | 13 | 0.01383 | 9 |
| p | 1 | 1 | 1.698 | 0.00773 | 64 | 0.00174 | 10 | 0.01297 | 9 |
| p | 1 | 1 | 1.868 | 0.00847 | 71 | 0.00206 | 9 | 0.01285 | 9 |
| p | 1 | 2 | 1.658 | 0.00849 | 70 | 0.00254 | 11 | 0.01575 | 9 |
| p | 1 | 2 | 1.616 | 0.00977 | 72 | 0.00372 | 12 | 0.01446 | 9 |
| p | 1 | 2 | 1.566 | 0.00903 | 73 | 0.00303 | 12 | 0.01319 | 9 |
| p | 1 | 3 | 1.492 | 0.00882 | 73 | 0.0019 | 9 | 0.01465 | 9 |
| p | 1 | 3 | 1.744 | 0.0093 | 72 | 0.00212 | 8 | 0.01572 | 9 |
| p | 1 | 3 | 1.514 | 0.00909 | 72 | 0.00192 | 9 | 0.01701 | 9 |
| p | 2 | 1 | 1.59 | 0.01019 | 58 | 0.00684 | 10 | 0.01603 | 40 |
| p | 2 | 1 | 1.924 | 0.00858 | 57 | 0.00545 | 11 | 0.01469 | 40 |
| p | 2 | 1 | 1.638 | 0.00705 | 40 | 0.00544 | 5 | 0.01271 | 40 |
| p | 2 | 1 | 1.864 | 0.01142 | 65 | 0.00581 | 8 | 0.01955 | 40 |
| p | 2 | 1 | 1.726 | 0.01077 | 64 | 0.00684 | 12 | 0.01552 | 40 |
| p | 2 | 1 | 1.726 | 0.00867 | 55 | 0.00624 | 11 | 0.01573 | 40 |
| p | 2 | 1 | 1.824 | 0.01071 | 59 | 0.00567 | 12 | 0.01697 | 40 |
| p | 2 | 1 | 1.934 | 0.00916 | 54 | 0.00641 | 11 | 0.01604 | 40 |
| p | 2 | 1 | 1.616 | 0.00823 | 52 | 0.00549 | 8 | 0.01433 | 40 |
| p | 2 | 1 | 1.718 | 0.01052 | 65 | 0.00529 | 8 | 0.01524 | 40 |
| p | 2 | 1 | 1.728 | 0.0104 | 66 | 0.00629 | 12 | 0.01504 | 40 |
| p | 2 | 1 | 1.798 | 0.01075 | 63 | 0.00563 | 8 | 0.01592 | 40 |
| p | 2 | 1 | 1.806 | 0.0102 | 61 | 0.00683 | 8 | 0.01661 | 40 |
| p | 2 | 2 | 1.714 | 0.01189 | 75 | 0.00417 | 8 | 0.01836 | 40 |
| p | 2 | 2 | 1.786 | 0.01167 | 69 | 0.00437 | 8 | 0.01821 | 40 |
| p | 2 | 2 | 1.876 | 0.01065 | 60 | 0.00343 | 8 | 0.01835 | 40 |
| p | 2 | 2 | 1.702 | 0.01176 | 68 | 0.00473 | 8 | 0.01857 | 40 |
| p | 2 | 2 | 1.792 | 0.01016 | 64 | 0.00352 | 8 | 0.01693 | 40 |
| p | 2 | 2 | 1.722 | 0.00964 | 61 | 0.00595 | 8 | 0.01506 | 40 |
| p | 2 | 2 | 1.452 | 0.01151 | 70 | 0.00411 | 8 | 0.01725 | 40 |
| p | 2 | 2 | 1.642 | 0.00793 | 50 | 0.00408 | 8 | 0.01374 | 40 |
| p | 2 | 2 | 1.6 | 0.01023 | 67 | 0.00393 | 7 | 0.01625 | 40 |
| p | 2 | 2 | 1.648 | 0.00684 | 47 | 0.00513 | 8 | 0.01359 | 40 |
| p | 2 | 2 | 1.768 | 0.00964 | 59 | 0.00543 | 10 | 0.01665 | 40 |
| p | 2 | 2 | 1.666 | 0.00979 | 61 | 0.00432 | 8 | 0.01632 | 40 |
| p | 2 | 2 | 1.796 | 0.01095 | 67 | 0.00377 | 9 | 0.01628 | 40 |
| p | 2 | 2 | 1.658 | 0.0091 | 55 | 0.00341 | 8 | 0.01514 | 40 |
| p | 2 | 3 | 1.788 | 0.01179 | 71 | 0.00455 | 8 | 0.01761 | 40 |
| p | 2 | 3 | 1.688 | 0.00994 | 63 | 0.00608 | 8 | 0.01664 | 40 |

| | | | | | | | | | |
|---|---|---|---|---|---|---|---|---|---|
| p | 2 | 3 | 1.708 | 0.00796 | 57 | 0.00416 | 8 | 0.01687 | 40 |
| p | 2 | 3 | 1.406 | 0.00875 | 63 | 0.0036 | 8 | 0.01647 | 40 |
| p | 2 | 3 | 1.762 | 0.01139 | 70 | 0.00662 | 8 | 0.02112 | 40 |
| p | 2 | 3 | 1.668 | 0.01016 | 54 | 0.00314 | 9 | 0.01912 | 40 |
| p | 2 | 3 | 1.638 | 0.00893 | 65 | 0.00273 | 8 | 0.01857 | 40 |
| p | 2 | 3 | 1.812 | 0.00927 | 55 | 0.00388 | 8 | 0.01712 | 40 |
| p | 2 | 3 | 1.69 | 0.01025 | 63 | 0.00362 | 8 | 0.02152 | 40 |
| p | 2 | 3 | 1.728 | 0.01373 | 79 | 0.01109 | 11 | 0.02192 | 40 |
| p | 2 | 3 | 1.556 | 0.00643 | 48 | 0.00396 | 8 | 0.0173 | 40 |
| p | 2 | 3 | 1.574 | 0.01141 | 61 | 0.00356 | 7 | 0.02361 | 40 |
| p | 2 | 3 | 1.854 | 0.01208 | 69 | 0.00442 | 8 | 0.02268 | 40 |
| p | 3 | 1 | 1.874 | 0.01009 | 74 | 0.00627 | 13 | 0.0159 | 20 |
| p | 3 | 1 | 1.822 | 0.00976 | 71 | 0.00719 | 13 | 0.01667 | 20 |
| p | 3 | 1 | 2.054 | 0.01065 | 69 | 0.00781 | 14 | 0.01903 | 20 |
| p | 3 | 1 | 1.858 | 0.0098 | 71 | 0.00546 | 14 | 0.01516 | 20 |
| p | 3 | 1 | 1.7 | 0.00984 | 71 | 0.00737 | 14 | 0.01539 | 20 |
| p | 3 | 1 | 1.868 | 0.00987 | 77 | 0.00675 | 13 | 0.01505 | 20 |
| p | 3 | 2 | 1.532 | 0.00948 | 71 | 0.00729 | 13 | 0.01408 | 20 |
| p | 3 | 2 | 1.72 | 0.00955 | 75 | 0.00566 | 11 | 0.01425 | 20 |
| p | 3 | 2 | 1.948 | 0.00671 | 60 | 0.00189 | 8 | 0.01257 | 20 |
| p | 3 | 2 | 1.594 | 0.00661 | 65 | 0.00182 | 8 | 0.00971 | 20 |
| p | 3 | 2 | 1.61 | 0.00685 | 66 | 0.00224 | 8 | 0.01086 | 20 |
| p | 3 | 2 | 1.568 | 0.00701 | 66 | 0.00208 | 7 | 0.01022 | 20 |
| p | 3 | 2 | 1.548 | 0.00752 | 65 | 0.0021 | 8 | 0.01055 | 20 |
| p | 3 | 2 | 1.418 | 0.00691 | 57 | 0.00169 | 8 | 0.01052 | 20 |
| p | 3 | 3 | 1.554 | 0.00729 | 75 | 0.00176 | 8 | 0.0105 | 20 |
| p | 3 | 3 | 1.578 | 0.00623 | 55 | 0.00141 | 8 | 0.01044 | 20 |
| p | 3 | 3 | 1.748 | 0.00763 | 64 | 0.00178 | 8 | 0.01085 | 20 |
| p | 3 | 3 | 1.744 | 0.00707 | 71 | 0.00168 | 8 | 0.01119 | 20 |
| p | 3 | 3 | 1.59 | 0.00743 | 66 | 0.00215 | 8 | 0.0123 | 20 |
| p | 3 | 3 | 1.752 | 0.00722 | 66 | 0.00168 | 6 | 0.01178 | 20 |
| p | 6 | 1 | 1.928 | 0.0075 | 62 | 0.00471 | 12 | 0.01762 | 13 |
| p | 6 | 1 | 1.716 | 0.00459 | 49 | 0.00199 | 7 | 0.01046 | 13 |
| p | 6 | 1 | 1.832 | 0.00876 | 71 | 0.00573 | 12 | 0.01637 | 13 |
| p | 6 | 1 | 1.828 | 0.01047 | 71 | 0.00605 | 11 | 0.01992 | 13 |
| p | 6 | 2 | 1.922 | 0.00944 | 77 | 0.00534 | 13 | 0.01836 | 13 |
| p | 6 | 2 | 1.722 | 0.00806 | 74 | 0.00521 | 9 | 0.01446 | 13 |
| p | 6 | 2 | 1.874 | 0.00922 | 70 | 0.0064 | 9 | 0.01578 | 13 |
| p | 6 | 2 | 1.468 | 0.00545 | 53 | 0.00225 | 8 | 0.01147 | 13 |
| p | 6 | 2 | 1.592 | 0.00516 | 45 | 0.00185 | 8 | 0.0114 | 13 |
| p | 6 | 3 | 1.468 | 0.00578 | 52 | 0.00134 | 8 | 0.01159 | 13 |
| p | 6 | 3 | 1.694 | 0.00613 | 53 | 0.00172 | 8 | 0.01261 | 13 |
| p | 6 | 3 | 1.574 | 0.00662 | 72 | 0.00503 | 9 | 0.01406 | 13 |

| | | | | | | | | | |
|---|---|---|---|---|---|---|---|---|---|
| p | 6 | 3 | 1.576 | 0.00749 | 78 | 0.00513 | 8 | 0.01582 | 13 |
| q | 1 | 1 | 1.646 | 0.00675 | 63 | 0.00245 | 8 | 0.01218 | 9 |
| q | 1 | 1 | 1.482 | 0.0066 | 60 | 0.00254 | 8 | 0.01146 | 9 |
| q | 1 | 1 | 1.582 | 0.00716 | 52 | 0.0019 | 8 | 0.01163 | 9 |
| q | 1 | 2 | 1.614 | 0.00647 | 48 | 0.00165 | 8 | 0.01104 | 9 |
| q | 1 | 2 | 1.712 | 0.00658 | 57 | 0.00396 | 8 | 0.01278 | 9 |
| q | 1 | 2 | 1.648 | 0.00813 | 62 | 0.00149 | 8 | 0.01304 | 9 |
| q | 1 | 3 | 1.576 | 0.00844 | 57 | 0.00124 | 8 | 0.01312 | 9 |
| q | 1 | 3 | 1.514 | 0.00838 | 56 | 0.00178 | 8 | 0.01395 | 9 |
| q | 1 | 3 | 1.535 | 0.00808 | 58 | 0.00167 | 8 | 0.01344 | 9 |
| q | 2 | 1 | 1.748 | 0.00986 | 70 | 0.00768 | 13 | 0.01461 | 13 |
| q | 2 | 1 | 1.744 | 0.0111 | 78 | 0.00622 | 13 | 0.01653 | 13 |
| q | 2 | 1 | 1.616 | 0.01157 | 73 | 0.00267 | 8 | 0.01731 | 13 |
| q | 2 | 1 | 1.648 | 0.01097 | 77 | 0.0023 | 9 | 0.01553 | 13 |
| q | 2 | 2 | 1.628 | 0.01132 | 80 | 0.00265 | 8 | 0.01667 | 13 |
| q | 2 | 2 | 1.568 | 0.01093 | 77 | 0.00291 | 8 | 0.01534 | 13 |
| q | 2 | 2 | 1.532 | 0.01074 | 79 | 0.00222 | 7 | 0.01437 | 13 |
| q | 2 | 2 | 1.776 | 0.01002 | 78 | 0.00304 | 8 | 0.01445 | 13 |
| q | 2 | 2 | 1.458 | 0.00991 | 78 | 0.00316 | 7 | 0.01449 | 13 |
| q | 2 | 3 | 1.628 | 0.00713 | 54 | 0.00202 | 5 | 0.01371 | 13 |
| q | 2 | 3 | 1.566 | 0.00717 | 57 | 0.00239 | 5 | 0.0114 | 13 |
| q | 2 | 3 | 1.428 | 0.00748 | 54 | 0.00178 | 5 | 0.01537 | 13 |
| q | 2 | 3 | 1.726 | 0.00768 | 60 | 0.00118 | 5 | 0.0136 | 13 |
| q | 4 | 1 | 1.268 | 0.00457 | 60 | 0.00043 | 5 | 0.00631 | 10 |
| q | 4 | 1 | 1.41 | 0.00714 | 62 | 0.00098 | 7 | 0.01043 | 10 |
| q | 4 | 1 | 1.344 | 0.00736 | 65 | 0.00122 | 6 | 0.01107 | 10 |
| q | 4 | 1 | 1.426 | 0.00695 | 69 | 0.00136 | 6 | 0.00972 | 10 |
| q | 4 | 2 | 1.214 | 0.00434 | 59 | 0.00023 | 5 | 0.00538 | 10 |
| q | 4 | 2 | 1.294 | 0.00665 | 66 | 0.00142 | 7 | 0.01097 | 10 |
| q | 4 | 2 | 1.226 | 0.00739 | 64 | 0.00135 | 7 | 0.01095 | 10 |
| q | 4 | 3 | 1.178 | 0.00543 | 62 | 0.00025 | 5 | 0.00514 | 10 |
| q | 4 | 3 | 1.228 | 0.00684 | 68 | 0.00159 | 6 | 0.01094 | 10 |
| q | 4 | 3 | 1.452 | 0.00709 | 65 | 0.00113 | 5 | 0.01128 | 10 |
| q | 5 | 1 | 1.802 | 0.00492 | 53 | 0.00107 | 7 | 0.00825 | 9 |
| q | 5 | 1 | 1.865 | 0.00512 | 51 | 0.00112 | 8 | 0.00811 | 9 |
| q | 5 | 1 | 1.787 | 0.00564 | 52 | 0.00099 | 7 | 0.00797 | 9 |
| q | 5 | 2 | 1.746 | 0.00434 | 55 | 0.00087 | 6 | 0.00725 | 9 |
| q | 5 | 2 | 1.723 | 0.00455 | 53 | 0.00082 | 6 | 0.00665 | 9 |
| q | 5 | 2 | 1.776 | 0.00489 | 56 | 0.0009 | 6 | 0.00712 | 9 |
| q | 5 | 3 | 1.656 | 0.00504 | 62 | 0.00095 | 6 | 0.00812 | 9 |
| q | 5 | 3 | 1.621 | 0.00554 | 60 | 0.00091 | 6 | 0.00824 | 9 |
| q | 5 | 3 | 1.611 | 0.00532 | 58 | 0.00088 | 6 | 0.00788 | 9 |
| r | 1 | 1 | 1.77 | 0.01369 | 81 | 0.00586 | 12 | 0.01924 | 35 |

| | | | | | | | | | |
|---|---|---|---|---|---|---|---|---|---|
| r | 1 | 1 | 1.858 | 0.01335 | 81 | 0.00817 | 14 | 0.01797 | 35 |
| r | 1 | 1 | 1.718 | 0.01244 | 71 | 0.0067 | 13 | 0.01785 | 35 |
| r | 1 | 1 | 1.618 | 0.00933 | 72 | 0.00779 | 10 | 0.01846 | 35 |
| r | 1 | 1 | 1.706 | 0.01208 | 86 | 0.00433 | 8 | 0.01755 | 35 |
| r | 1 | 1 | 1.826 | 0.01275 | 87 | 0.00757 | 10 | 0.01772 | 35 |
| r | 1 | 1 | 1.804 | 0.01033 | 68 | 0.00784 | 13 | 0.01746 | 35 |
| r | 1 | 1 | 1.616 | 0.01111 | 72 | 0.00499 | 8 | 0.01752 | 35 |
| r | 1 | 1 | 1.796 | 0.01249 | 85 | 0.00908 | 12 | 0.01898 | 35 |
| r | 1 | 1 | 1.564 | 0.00504 | 40 | 0.00435 | 7 | 0.01661 | 35 |
| r | 1 | 1 | 1.652 | 0.01259 | 87 | 0.00565 | 12 | 0.01894 | 35 |
| r | 1 | 2 | 1.514 | 0.01167 | 90 | 0.00537 | 12 | 0.01796 | 35 |
| r | 1 | 2 | 1.65 | 0.01311 | 77 | 0.00912 | 10 | 0.02014 | 35 |
| r | 1 | 2 | 1.58 | 0.0092 | 56 | 0.00959 | 11 | 0.0179 | 35 |
| r | 1 | 2 | 1.56 | 0.01236 | 90 | 0.00745 | 13 | 0.01868 | 35 |
| r | 1 | 2 | 1.776 | 0.01382 | 93 | 0.00716 | 11 | 0.02496 | 35 |
| r | 1 | 2 | 1.726 | 0.00981 | 73 | 0.00316 | 8 | 0.01831 | 35 |
| r | 1 | 2 | 1.68 | 0.00826 | 48 | 0.00236 | 4 | 0.01962 | 35 |
| r | 1 | 2 | 1.478 | 0.01378 | 94 | 0.00559 | 9 | 0.01911 | 35 |
| r | 1 | 2 | 1.438 | 0.01025 | 84 | 0.0028 | 8 | 0.01821 | 35 |
| r | 1 | 2 | 1.752 | 0.00996 | 84 | 0.00381 | 8 | 0.0185 | 35 |
| r | 1 | 2 | 1.576 | 0.01236 | 89 | 0.00389 | 8 | 0.02171 | 35 |
| r | 1 | 2 | 1.432 | 0.00872 | 75 | 0.00358 | 8 | 0.01588 | 35 |
| r | 1 | 2 | 1.648 | 0.01284 | 82 | 0.00398 | 8 | 0.02197 | 35 |
| r | 1 | 3 | 1.654 | 0.01142 | 73 | 0.00552 | 13 | 0.02051 | 35 |
| r | 1 | 3 | 1.696 | 0.01332 | 80 | 0.00471 | 8 | 0.02547 | 35 |
| r | 1 | 3 | 1.686 | 0.00806 | 51 | 0.00473 | 9 | 0.01757 | 35 |
| r | 1 | 3 | 1.672 | 0.00827 | 56 | 0.00263 | 9 | 0.0175 | 35 |
| r | 1 | 3 | 1.668 | 0.01453 | 84 | 0.00336 | 8 | 0.0223 | 35 |
| r | 1 | 3 | 1.716 | 0.01276 | 68 | 0.00257 | 8 | 0.0214 | 35 |
| r | 1 | 3 | 1.7 | 0.0094 | 65 | 0.00327 | 9 | 0.01744 | 35 |
| r | 1 | 3 | 1.682 | 0.01121 | 73 | 0.00483 | 10 | 0.02269 | 35 |
| r | 1 | 3 | 1.538 | 0.01306 | 79 | 0.00165 | 7 | 0.02479 | 35 |
| r | 1 | 3 | 1.544 | 0.01652 | 91 | 0.00328 | 8 | 0.0287 | 35 |
| r | 1 | 3 | 1.612 | 0.01216 | 80 | 0.00262 | 7 | 0.02173 | 35 |
| r | 2 | 1 | 1.672 | 0.00852 | 53 | 0.00565 | 8 | 0.01448 | 9 |
| r | 2 | 1 | 1.888 | 0.0084 | 51 | 0.00491 | 8 | 0.01492 | 9 |
| r | 2 | 1 | 1.742 | 0.00875 | 50 | 0.00504 | 8 | 0.01652 | 9 |
| r | 2 | 2 | 1.852 | 0.00826 | 58 | 0.00365 | 8 | 0.01388 | 9 |
| r | 2 | 2 | 1.888 | 0.00843 | 56 | 0.00334 | 8 | 0.01401 | 9 |
| r | 2 | 2 | 1.906 | 0.00871 | 53 | 0.00516 | 8 | 0.01569 | 9 |
| r | 2 | 3 | 1.788 | 0.00792 | 58 | 0.00337 | 8 | 0.01585 | 9 |
| r | 2 | 3 | 1.866 | 0.00877 | 55 | 0.00452 | 8 | 0.01685 | 9 |
| r | 2 | 3 | 1.632 | 0.00909 | 58 | 0.0043 | 8 | 0.0156 | 9 |

| | | | | | | | | | |
|---|---|---|---|---|---|---|---|---|---|
| r | 3 | 1 | 1.826 | 0.0092 | 65 | 0.00553 | 14 | 0.01393 | 23 |
| r | 3 | 1 | 1.928 | 0.00902 | 67 | 0.00481 | 14 | 0.0147 | 23 |
| r | 3 | 1 | 2.2 | 0.01058 | 68 | 0.00883 | 14 | 0.01399 | 23 |
| r | 3 | 1 | 1.884 | 0.0104 | 70 | 0.00582 | 11 | 0.01322 | 23 |
| r | 3 | 1 | 1.68 | 0.01049 | 71 | 0.00478 | 11 | 0.01373 | 23 |
| r | 3 | 1 | 1.792 | 0.00808 | 57 | 0.00637 | 11 | 0.01439 | 23 |
| r | 3 | 1 | 1.684 | 0.00993 | 68 | 0.00556 | 13 | 0.01476 | 23 |
| r | 3 | 2 | 1.73 | 0.01033 | 68 | 0.00681 | 9 | 0.01467 | 23 |
| r | 3 | 2 | 1.986 | 0.01088 | 73 | 0.00386 | 8 | 0.01552 | 23 |
| r | 3 | 2 | 1.674 | 0.00893 | 73 | 0.00467 | 9 | 0.01318 | 23 |
| r | 3 | 2 | 1.628 | 0.00687 | 64 | 0.00525 | 12 | 0.01226 | 23 |
| r | 3 | 2 | 1.968 | 0.01046 | 75 | 0.00453 | 8 | 0.01433 | 23 |
| r | 3 | 2 | 1.614 | 0.00817 | 55 | 0.0061 | 8 | 0.01424 | 23 |
| r | 3 | 2 | 1.62 | 0.00897 | 65 | 0.00575 | 9 | 0.0145 | 23 |
| r | 3 | 2 | 1.718 | 0.00943 | 71 | 0.00464 | 10 | 0.01338 | 23 |
| r | 3 | 2 | 1.584 | 0.0086 | 73 | 0.00477 | 8 | 0.01257 | 23 |
| r | 3 | 3 | 1.744 | 0.00933 | 73 | 0.00527 | 8 | 0.01385 | 23 |
| r | 3 | 3 | 1.644 | 0.00815 | 63 | 0.00437 | 7 | 0.0137 | 23 |
| r | 3 | 3 | 1.578 | 0.00839 | 71 | 0.00287 | 8 | 0.01387 | 23 |
| r | 3 | 3 | 1.87 | 0.00808 | 60 | 0.01231 | 9 | 0.01472 | 23 |
| r | 3 | 3 | 1.686 | 0.00737 | 60 | 0.0022 | 7 | 0.01421 | 23 |
| r | 3 | 3 | 1.794 | 0.00843 | 62 | 0.00283 | 8 | 0.01643 | 23 |
| r | 3 | 3 | 1.678 | 0.00796 | 72 | 0.00264 | 8 | 0.01592 | 23 |
| r | 4 | 1 | 1.778 | 0.00612 | 58 | 0.00224 | 8 | 0.01055 | 9 |
| r | 4 | 1 | 1.542 | 0.00654 | 62 | 0.00145 | 8 | 0.01027 | 9 |
| r | 4 | 1 | 1.61 | 0.00666 | 63 | 0.0017 | 8 | 0.00976 | 9 |
| r | 4 | 2 | 1.72 | 0.00659 | 65 | 0.00217 | 8 | 0.00997 | 9 |
| r | 4 | 2 | 1.642 | 0.00598 | 64 | 0.00166 | 7 | 0.00983 | 9 |
| r | 4 | 2 | 1.728 | 0.00542 | 50 | 0.00211 | 8 | 0.00974 | 9 |
| r | 4 | 3 | 1.858 | 0.0063 | 64 | 0.00187 | 7 | 0.00891 | 9 |
| r | 4 | 3 | 1.768 | 0.00617 | 69 | 0.00243 | 8 | 0.0101 | 9 |
| r | 4 | 3 | 1.694 | 0.00625 | 63 | 0.00203 | 8 | 0.01016 | 9 |
| s | 2 | 1 | 1.674 | 0.00673 | 56 | 0.00216 | 8 | 0.01107 | 10 |
| s | 2 | 1 | 1.676 | 0.00648 | 58 | 0.00269 | 8 | 0.01099 | 10 |
| s | 2 | 1 | 1.41 | 0.00536 | 57 | 0.00235 | 8 | 0.00906 | 10 |
| s | 2 | 2 | 1.674 | 0.00568 | 65 | 0.00185 | 8 | 0.00943 | 10 |
| s | 2 | 2 | 1.468 | 0.00598 | 63 | 0.0015 | 7 | 0.01002 | 10 |
| s | 2 | 2 | 1.622 | 0.00717 | 54 | 0.00179 | 8 | 0.01117 | 10 |
| s | 2 | 2 | 1.668 | 0.00778 | 68 | 0.00117 | 7 | 0.01157 | 10 |
| s | 2 | 3 | 1.522 | 0.00971 | 59 | 0.00133 | 8 | 0.00713 | 10 |
| s | 2 | 3 | 1.786 | 0.00734 | 65 | 0.00153 | 7 | 0.00917 | 10 |
| s | 2 | 3 | 1.338 | 0.00645 | 70 | 0.00111 | 5 | 0.00985 | 10 |
| s | 3 | 1 | 1.4 | 0.00545 | 58 | 0.00172 | 7 | 0.00764 | 12 |

| | | | | | | | | | |
|---|---|---|---|---|---|---|---|---|---|
| s | 3 | 1 | 1.562 | 0.00529 | 60 | 0.00132 | 7 | 0.00789 | 12 |
| s | 3 | 1 | 1.456 | 0.00544 | 60 | 0.00149 | 8 | 0.0069 | 12 |
| s | 3 | 1 | 1.56 | 0.00547 | 61 | 0.00132 | 7 | 0.00772 | 12 |
| s | 3 | 2 | 1.538 | 0.00563 | 60 | 0.00144 | 8 | 0.00788 | 12 |
| s | 3 | 2 | 1.506 | 0.00581 | 61 | 0.00111 | 8 | 0.00812 | 12 |
| s | 3 | 2 | 1.618 | 0.00607 | 59 | 0.00109 | 8 | 0.00809 | 12 |
| s | 3 | 2 | 1.514 | 0.00559 | 60 | 0.00101 | 7 | 0.00801 | 12 |
| s | 3 | 3 | 1.72 | 0.0051 | 59 | 0.00132 | 7 | 0.00811 | 12 |
| s | 3 | 3 | 1.596 | 0.00554 | 62 | 0.00161 | 7 | 0.00868 | 12 |
| s | 3 | 3 | 1.728 | 0.00565 | 63 | 0.00097 | 6 | 0.0083 | 12 |
| s | 3 | 3 | 1.656 | 0.00597 | 59 | 0.00098 | 7 | 0.00913 | 12 |
| s | 4 | 1 | 1.874 | 0.00533 | 44 | 0.00086 | 7 | 0.01009 | 15 |
| s | 4 | 1 | 1.82 | 0.00616 | 52 | 0.00128 | 6 | 0.01107 | 15 |
| s | 4 | 1 | 1.676 | 0.01195 | 73 | 0.00909 | 18 | 0.02318 | 15 |
| s | 4 | 1 | 1.924 | 0.01129 | 64 | 0.0105 | 18 | 0.02288 | 15 |
| s | 4 | 1 | 1.78 | 0.01089 | 76 | 0.0052 | 12 | 0.01805 | 15 |
| s | 4 | 2 | 1.774 | 0.0122 | 73 | 0.00732 | 15 | 0.02014 | 15 |
| s | 4 | 2 | 1.7 | 0.01063 | 72 | 0.00884 | 16 | 0.0195 | 15 |
| s | 4 | 2 | 1.738 | 0.01146 | 77 | 0.00739 | 15 | 0.02281 | 15 |
| s | 4 | 2 | 1.58 | 0.00546 | 58 | 0.00216 | 8 | 0.01009 | 15 |
| s | 4 | 2 | 1.746 | 0.00666 | 62 | 0.0014 | 8 | 0.01136 | 15 |
| s | 4 | 3 | 1.648 | 0.00672 | 62 | 0.00185 | 8 | 0.01056 | 15 |
| s | 4 | 3 | 1.916 | 0.00586 | 64 | 0.00175 | 8 | 0.01169 | 15 |
| s | 4 | 3 | 1.588 | 0.00645 | 64 | 0.00153 | 7 | 0.01206 | 15 |
| s | 4 | 3 | 1.854 | 0.01111 | 81 | 0.00549 | 13 | 0.01845 | 15 |
| s | 4 | 3 | 1.834 | 0.01078 | 74 | 0.00558 | 13 | 0.01854 | 15 |
| s | 6 | 1 | 1.63 | 0.0133 | 85 | 0.00544 | 14 | 0.02051 | 12 |
| s | 6 | 1 | 1.694 | 0.0116 | 79 | 0.00512 | 13 | 0.01866 | 12 |
| s | 6 | 1 | 1.732 | 0.01228 | 79 | 0.0076 | 14 | 0.01946 | 12 |
| s | 6 | 1 | 1.792 | 0.01231 | 80 | 0.00499 | 13 | 0.01993 | 12 |
| s | 6 | 2 | 1.648 | 0.0131 | 82 | 0.00455 | 12 | 0.01982 | 12 |
| s | 6 | 2 | 1.748 | 0.01207 | 79 | 0.00558 | 12 | 0.02036 | 12 |
| s | 6 | 2 | 1.534 | 0.01274 | 81 | 0.00343 | 10 | 0.02022 | 12 |
| s | 6 | 2 | 1.694 | 0.01321 | 82 | 0.00357 | 9 | 0.02228 | 12 |
| s | 6 | 3 | 1.776 | 0.0124 | 83 | 0.00447 | 12 | 0.01852 | 12 |
| s | 6 | 3 | 1.572 | 0.01154 | 80 | 0.00466 | 9 | 0.01964 | 12 |
| s | 6 | 3 | 1.768 | 0.0116 | 81 | 0.00461 | 9 | 0.01844 | 12 |
| s | 6 | 3 | 1.5 | 0.01047 | 78 | 0.00406 | 9 | 0.01826 | 12 |
| t | 1 | 1 | 1.562 | 0.00647 | 56 | 0.00311 | 8 | 0.01333 | 14 |
| t | 1 | 1 | 1.464 | 0.00687 | 60 | 0.00247 | 8 | 0.01249 | 14 |
| t | 1 | 1 | 1.578 | 0.0062 | 56 | 0.00335 | 8 | 0.01321 | 14 |
| t | 1 | 1 | 1.542 | 0.00631 | 58 | 0.0024 | 8 | 0.01196 | 14 |
| t | 1 | 2 | 1.492 | 0.00657 | 58 | 0.00338 | 8 | 0.01336 | 14 |

| | | | | | | | | | |
|---|---|---|---|---|---|---|---|---|---|
| t | 1 | 2 | 1.466 | 0.00567 | 58 | 0.00212 | 8 | 0.01079 | 14 |
| t | 1 | 2 | 1.672 | 0.00659 | 60 | 0.00237 | 8 | 0.01171 | 14 |
| t | 1 | 2 | 1.75 | 0.00623 | 56 | 0.00345 | 8 | 0.01209 | 14 |
| t | 1 | 2 | 1.594 | 0.00582 | 58 | 0.00293 | 8 | 0.01137 | 14 |
| t | 1 | 2 | 1.62 | 0.0081 | 64 | 0.0023 | 7 | 0.01346 | 14 |
| t | 1 | 3 | 1.356 | 0.00739 | 63 | 0.00142 | 6 | 0.01329 | 14 |
| t | 1 | 3 | 1.414 | 0.00556 | 53 | 0.00294 | 8 | 0.01192 | 14 |
| t | 1 | 3 | 1.438 | 0.0048 | 57 | 0.00242 | 8 | 0.01283 | 14 |
| t | 1 | 3 | 1.422 | 0.00538 | 61 | 0.00262 | 8 | 0.01339 | 14 |
| t | 4 | 1 | 1.258 | 0.00447 | 48 | 0.00158 | 9 | 0.00931 | 10 |
| t | 4 | 1 | 1.548 | 0.00523 | 56 | 0.00132 | 9 | 0.00811 | 10 |
| t | 4 | 1 | 1.492 | 0.00619 | 62 | 0.00132 | 8 | 0.00986 | 10 |
| t | 4 | 2 | 1.162 | 0.00619 | 51 | 0.00115 | 7 | 0.00944 | 10 |
| t | 4 | 2 | 1.532 | 0.00582 | 59 | 0.00112 | 8 | 0.0087 | 10 |
| t | 4 | 2 | 1.512 | 0.00531 | 57 | 0.00124 | 8 | 0.00825 | 10 |
| t | 4 | 2 | 1.534 | 0.00544 | 56 | 0.00097 | 8 | 0.00831 | 10 |
| t | 4 | 3 | 1.68 | 0.00509 | 59 | 0.00118 | 8 | 0.00868 | 10 |
| t | 4 | 3 | 1.464 | 0.00557 | 56 | 0.00083 | 7 | 0.0086 | 10 |
| t | 4 | 3 | 1.492 | 0.00516 | 64 | 0.00116 | 7 | 0.00839 | 10 |
| t | 5 | 1 | 1.62 | 0.00831 | 58 | 0.00223 | 8 | 0.01329 | 11 |
| t | 5 | 1 | 1.748 | 0.00901 | 61 | 0.0024 | 8 | 0.01353 | 11 |
| t | 5 | 1 | 1.76 | 0.00825 | 61 | 0.00188 | 7 | 0.01658 | 11 |
| t | 5 | 2 | 1.814 | 0.00859 | 63 | 0.00217 | 8 | 0.01395 | 11 |
| t | 5 | 2 | 1.81 | 0.00907 | 61 | 0.00192 | 8 | 0.01409 | 11 |
| t | 5 | 2 | 1.806 | 0.00821 | 60 | 0.00141 | 7 | 0.01323 | 11 |
| t | 5 | 2 | 1.81 | 0.00833 | 66 | 0.00174 | 8 | 0.01482 | 11 |
| t | 5 | 2 | 1.466 | 0.00799 | 65 | 0.00213 | 8 | 0.01479 | 11 |
| t | 5 | 3 | 1.616 | 0.00798 | 61 | 0.00122 | 5 | 0.01355 | 11 |
| t | 5 | 3 | 1.492 | 0.00569 | 59 | 0.00238 | 8 | 0.01247 | 11 |
| t | 5 | 3 | 1.354 | 0.00602 | 58 | 0.0023 | 8 | 0.01187 | 11 |
| t | 6 | 1 | 1.652 | 0.00771 | 63 | 0.00228 | 8 | 0.01367 | 10 |
| t | 6 | 1 | 1.658 | 0.00824 | 66 | 0.00248 | 8 | 0.01339 | 10 |
| t | 6 | 1 | 1.756 | 0.00734 | 66 | 0.00175 | 8 | 0.01378 | 10 |
| t | 6 | 2 | 1.638 | 0.00816 | 65 | 0.00164 | 8 | 0.01357 | 10 |
| t | 6 | 2 | 1.694 | 0.00792 | 61 | 0.00161 | 7 | 0.01367 | 10 |
| t | 6 | 2 | 1.854 | 0.00828 | 66 | 0.00214 | 8 | 0.01503 | 10 |
| t | 6 | 2 | 1.612 | 0.00773 | 58 | 0.00154 | 6 | 0.01462 | 10 |
| t | 6 | 3 | 1.736 | 0.00791 | 65 | 0.00224 | 6 | 0.01471 | 10 |
| t | 6 | 3 | 1.58 | 0.00744 | 63 | 0.00181 | 7 | 0.01385 | 10 |
| t | 6 | 3 | 1.846 | 0.00887 | 69 | 0.00386 | 8 | 0.01438 | 10 |
| u | 1 | 1 | 1.892 | 0.00699 | 55 | 0.00459 | 11 | 0.013 | 12 |
| u | 1 | 1 | 1.878 | 0.00799 | 56 | 0.0067 | 11 | 0.01549 | 12 |
| u | 1 | 1 | 1.958 | 0.0082 | 59 | 0.0046 | 8 | 0.011 | 12 |

| | | | | | | | | | |
|---|---|---|---|---|---|---|---|---|---|
| u | 1 | 1 | 1.912 | 0.00696 | 51 | 0.00603 | 13 | 0.01402 | 12 |
| u | 1 | 2 | 1.708 | 0.00874 | 62 | 0.00432 | 8 | 0.0166 | 12 |
| u | 1 | 2 | 1.988 | 0.00859 | 63 | 0.0027 | 8 | 0.01652 | 12 |
| u | 1 | 2 | 2.004 | 0.00711 | 56 | 0.00279 | 9 | 0.01474 | 12 |
| u | 1 | 2 | 2.06 | 0.00837 | 60 | 0.00635 | 13 | 0.01463 | 12 |
| u | 1 | 3 | 1.984 | 0.00755 | 62 | 0.00314 | 9 | 0.01635 | 12 |
| u | 1 | 3 | 1.79 | 0.00793 | 55 | 0.00561 | 13 | 0.01523 | 12 |
| u | 1 | 3 | 1.768 | 0.00847 | 64 | 0.00308 | 8 | 0.01632 | 12 |
| u | 1 | 3 | 1.868 | 0.00787 | 61 | 0.00256 | 8 | 0.01587 | 12 |
| u | 2 | 1 | 1.75 | 0.00576 | 48 | 0.00237 | 8 | 0.0125 | 11 |
| u | 2 | 1 | 1.716 | 0.00745 | 59 | 0.00151 | 8 | 0.01269 | 11 |
| u | 2 | 1 | 1.654 | 0.00646 | 58 | 0.00228 | 8 | 0.01189 | 11 |
| u | 2 | 2 | 1.786 | 0.00733 | 58 | 0.00222 | 8 | 0.01183 | 11 |
| u | 2 | 2 | 1.75 | 0.00832 | 63 | 0.00327 | 13 | 0.01304 | 11 |
| u | 2 | 2 | 1.622 | 0.00873 | 64 | 0.00315 | 13 | 0.01348 | 11 |
| u | 2 | 2 | 1.782 | 0.009 | 65 | 0.00392 | 13 | 0.01527 | 11 |
| u | 2 | 2 | 1.618 | 0.0066 | 59 | 0.00176 | 8 | 0.0124 | 11 |
| u | 2 | 3 | 1.678 | 0.0075 | 67 | 0.00166 | 8 | 0.0127 | 11 |
| u | 2 | 3 | 1.7 | 0.0067 | 62 | 0.0017 | 7 | 0.0105 | 11 |
| u | 2 | 3 | 1.846 | 0.0066 | 56 | 0.0017 | 8 | 0.0132 | 11 |
| u | 3 | 1 | 1.656 | 0.0066 | 55 | 0.00235 | 8 | 0.0158 | 15 |
| u | 3 | 1 | 1.746 | 0.0063 | 50 | 0.00293 | 8 | 0.0137 | 15 |
| u | 3 | 1 | 1.882 | 0.0066 | 52 | 0.0018 | 7 | 0.0142 | 15 |
| u | 3 | 1 | 1.83 | 0.0062 | 55 | 0.00187 | 8 | 0.014 | 15 |
| u | 3 | 1 | 1.77 | 0.0062 | 60 | 0.00142 | 8 | 0.0109 | 15 |
| u | 3 | 2 | 1.776 | 0.0063 | 55 | 0.00164 | 8 | 0.0124 | 15 |
| u | 3 | 2 | 1.728 | 0.0064 | 63 | 0.00214 | 8 | 0.0115 | 15 |
| u | 3 | 2 | 1.682 | 0.0065 | 54 | 0.00137 | 8 | 0.0117 | 15 |
| u | 3 | 2 | 1.712 | 0.0062 | 59 | 0.00267 | 8 | 0.0108 | 15 |
| u | 3 | 2 | 1.67 | 0.0049 | 58 | 0.00171 | 8 | 0.0113 | 15 |
| u | 3 | 3 | 1.692 | 0.0066 | 56 | 0.002 | 8 | 0.0123 | 15 |
| u | 3 | 3 | 1.684 | 0.0064 | 56 | 0.00199 | 8 | 0.0123 | 15 |
| u | 3 | 3 | 1.692 | 0.0066 | 52 | 0.00193 | 8 | 0.0117 | 15 |
| u | 3 | 3 | 1.63 | 0.0062 | 52 | 0.00135 | 7 | 0.0117 | 15 |
| u | 3 | 3 | 1.624 | 0.0054 | 50 | 0.00169 | 7 | 0.014 | 15 |
| u | 4 | 1 | 1.608 | 0.0056 | 51 | 0.00207 | 8 | 0.0115 | 11 |
| u | 4 | 1 | 1.546 | 0.0052 | 51 | 0.00231 | 8 | 0.0112 | 11 |
| u | 4 | 1 | 1.438 | 0.0053 | 49 | 0.00195 | 8 | 0.0113 | 11 |
| u | 4 | 2 | 1.62 | 0.005 | 52 | 0.00215 | 8 | 0.0117 | 11 |
| u | 4 | 2 | 1.716 | 0.0056 | 51 | 0.00132 | 8 | 0.0115 | 11 |
| u | 4 | 2 | 1.46 | 0.0049 | 50 | 0.00127 | 7 | 0.0106 | 11 |
| u | 4 | 2 | 1.656 | 0.0053 | 52 | 0.00186 | 8 | 0.0116 | 11 |
| u | 4 | 2 | 1.552 | 0.0057 | 52 | 0.00141 | 7 | 0.0116 | 11 |

| | | | | | | | | | |
|---|---|---|---|---|---|---|---|---|---|
| u | 4 | 3 | 1.694 | 0.0066 | 53 | 0.00135 | 7 | 0.0109 | 11 |
| u | 4 | 3 | 1.854 | 0.0059 | 58 | 0.00194 | 8 | 0.0119 | 11 |
| u | 4 | 3 | 1.584 | 0.0059 | 58 | 0.00162 | 7 | 0.0116 | 11 |
| v | 1 | 1 | 1.774 | 0.00933 | 51 | 0.00563 | 14 | 0.0177 | 9 |
| v | 1 | 1 | 1.754 | 0.0073 | 53 | 0.00357 | 8 | 0.01311 | 9 |
| v | 1 | 1 | 1.787 | 0.00834 | 53 | 0.00333 | 8 | 0.01422 | 9 |
| v | 1 | 2 | 1.796 | 0.00815 | 59 | 0.00312 | 8 | 0.01347 | 9 |
| v | 1 | 2 | 1.768 | 0.00798 | 60 | 0.00393 | 8 | 0.01372 | 9 |
| v | 1 | 2 | 1.72 | 0.00766 | 61 | 0.00319 | 8 | 0.0128 | 9 |
| v | 1 | 3 | 1.732 | 0.00699 | 63 | 0.00342 | 8 | 0.01294 | 9 |
| v | 1 | 3 | 1.672 | 0.00765 | 60 | 0.00251 | 8 | 0.01313 | 9 |
| v | 1 | 3 | 1.734 | 0.00636 | 58 | 0.00313 | 8 | 0.01323 | 9 |
| v | 2 | 1 | 1.822 | 0.00929 | 67 | 0.00548 | 9 | 0.01511 | 17 |
| v | 2 | 1 | 1.654 | 0.0085 | 69 | 0.00622 | 12 | 0.01452 | 17 |
| v | 2 | 1 | 1.622 | 0.00894 | 70 | 0.00513 | 11 | 0.01432 | 17 |
| v | 2 | 1 | 1.74 | 0.00962 | 73 | 0.00407 | 11 | 0.01444 | 17 |
| v | 2 | 1 | 1.522 | 0.01075 | 78 | 0.00474 | 10 | 0.01409 | 17 |
| v | 2 | 2 | 1.732 | 0.00962 | 78 | 0.00385 | 8 | 0.01333 | 17 |
| v | 2 | 2 | 1.494 | 0.0099 | 73 | 0.00316 | 8 | 0.01405 | 17 |
| v | 2 | 2 | 1.632 | 0.0088 | 75 | 0.00337 | 8 | 0.0145 | 17 |
| v | 2 | 2 | 1.562 | 0.01051 | 76 | 0.00293 | 8 | 0.0138 | 17 |
| v | 2 | 2 | 1.734 | 0.01012 | 76 | 0.00327 | 8 | 0.01368 | 17 |
| v | 2 | 2 | 1.61 | 0.0099 | 78 | 0.00363 | 9 | 0.01367 | 17 |
| v | 2 | 2 | 1.492 | 0.01007 | 75 | 0.00295 | 8 | 0.01438 | 17 |
| v | 2 | 3 | 1.506 | 0.01024 | 77 | 0.00283 | 8 | 0.01477 | 17 |
| v | 2 | 3 | 1.568 | 0.00968 | 76 | 0.00328 | 7 | 0.01435 | 17 |
| v | 2 | 3 | 1.516 | 0.00928 | 76 | 0.00305 | 8 | 0.01397 | 17 |
| v | 2 | 3 | 1.482 | 0.00994 | 71 | 0.00301 | 8 | 0.0146 | 17 |
| v | 2 | 3 | 1.57 | 0.01044 | 78 | 0.00329 | 8 | 0.01542 | 17 |
| v | 3 | 1 | 1.69 | 0.01094 | 73 | 0.00209 | 8 | 0.01361 | 15 |
| v | 3 | 1 | 1.842 | 0.01026 | 73 | 0.00195 | 8 | 0.0142 | 15 |
| v | 3 | 1 | 1.756 | 0.01068 | 72 | 0.00224 | 8 | 0.01384 | 15 |
| v | 3 | 1 | 1.71 | 0.01121 | 78 | 0.00243 | 8 | 0.01414 | 15 |
| v | 3 | 1 | 1.814 | 0.01079 | 77 | 0.00226 | 8 | 0.0143 | 15 |
| v | 3 | 2 | 1.8 | 0.01159 | 77 | 0.00206 | 8 | 0.01403 | 15 |
| v | 3 | 2 | 1.576 | 0.01109 | 84 | 0.00268 | 8 | 0.01322 | 15 |
| v | 3 | 2 | 1.676 | 0.01114 | 82 | 0.00231 | 8 | 0.01318 | 15 |
| v | 3 | 2 | 1.698 | 0.01026 | 86 | 0.00203 | 7 | 0.01355 | 15 |
| v | 3 | 2 | 1.66 | 0.01132 | 80 | 0.00251 | 8 | 0.01398 | 15 |
| v | 3 | 3 | 1.67 | 0.01284 | 83 | 0.00186 | 7 | 0.01427 | 15 |
| v | 3 | 3 | 1.794 | 0.01044 | 82 | 0.00215 | 7 | 0.01416 | 15 |
| v | 3 | 3 | 1.604 | 0.01056 | 80 | 0.00236 | 8 | 0.01455 | 15 |
| v | 3 | 3 | 1.926 | 0.01101 | 80 | 0.00238 | 8 | 0.01578 | 15 |

| | | | | | | | | | |
|---|---|---|---|---|---|---|---|---|---|
| v | 3 | 3 | 2.152 | 0.01002 | 68 | 0.00248 | 8 | 0.01524 | 15 |
| v | 4 | 1 | 1.682 | 0.01184 | 72 | 0.00914 | 14 | 0.01824 | 25 |
| v | 4 | 1 | 1.81 | 0.01108 | 74 | 0.00817 | 16 | 0.01823 | 25 |
| v | 4 | 1 | 1.74 | 0.0115 | 73 | 0.00946 | 16 | 0.01724 | 25 |
| v | 4 | 1 | 1.742 | 0.0114 | 75 | 0.00794 | 13 | 0.01845 | 25 |
| v | 4 | 1 | 1.828 | 0.01176 | 70 | 0.01164 | 19 | 0.02105 | 25 |
| v | 4 | 1 | 1.726 | 0.01132 | 73 | 0.0084 | 13 | 0.01673 | 25 |
| v | 4 | 1 | 1.634 | 0.01253 | 81 | 0.00634 | 12 | 0.01844 | 25 |
| v | 4 | 1 | 1.78 | 0.01184 | 75 | 0.00823 | 14 | 0.01809 | 25 |
| v | 4 | 2 | 1.678 | 0.01164 | 77 | 0.00658 | 11 | 0.01646 | 25 |
| v | 4 | 2 | 1.656 | 0.01132 | 78 | 0.00586 | 12 | 0.01553 | 25 |
| v | 4 | 2 | 1.636 | 0.01193 | 80 | 0.0054 | 9 | 0.01756 | 25 |
| v | 4 | 2 | 1.634 | 0.01047 | 78 | 0.00788 | 12 | 0.01632 | 25 |
| v | 4 | 2 | 1.756 | 0.01158 | 83 | 0.00661 | 11 | 0.01597 | 25 |
| v | 4 | 2 | 1.634 | 0.01086 | 82 | 0.00471 | 9 | 0.01627 | 25 |
| v | 4 | 2 | 1.706 | 0.01154 | 80 | 0.0063 | 10 | 0.01629 | 25 |
| v | 4 | 2 | 1.616 | 0.00968 | 78 | 0.00524 | 10 | 0.0176 | 25 |
| v | 4 | 2 | 1.672 | 0.01096 | 80 | 0.00535 | 10 | 0.01661 | 25 |
| v | 4 | 3 | 1.648 | 0.01076 | 78 | 0.00494 | 8 | 0.0168 | 25 |
| v | 4 | 3 | 1.702 | 0.00929 | 75 | 0.00432 | 8 | 0.01644 | 25 |
| v | 4 | 3 | 1.694 | 0.00708 | 55 | 0.00326 | 9 | 0.01307 | 25 |
| v | 4 | 3 | 1.786 | 0.00983 | 74 | 0.0061 | 9 | 0.01705 | 25 |
| v | 4 | 3 | 1.648 | 0.01086 | 79 | 0.00576 | 10 | 0.01739 | 25 |
| v | 4 | 3 | 1.638 | 0.00713 | 52 | 0.00459 | 7 | 0.01644 | 25 |
| v | 4 | 3 | 1.694 | 0.01036 | 76 | 0.00536 | 9 | 0.01756 | 25 |
| v | 4 | 3 | 1.674 | 0.00938 | 69 | 0.00514 | 8 | 0.01549 | 25 |
| w | 1 | 1 | 1.866 | 0.0048 | 48 | 0.00182 | 8 | 0.0102 | 21 |
| w | 1 | 1 | 1.748 | 0.0052 | 54 | 0.00193 | 8 | 0.0098 | 21 |
| w | 1 | 1 | 2.026 | 0.0051 | 53 | 0.00173 | 8 | 0.0111 | 21 |
| w | 1 | 1 | 1.622 | 0.0054 | 54 | 0.00169 | 8 | 0.0102 | 21 |
| w | 1 | 1 | 1.838 | 0.0064 | 52 | 0.00205 | 6 | 0.0124 | 21 |
| w | 1 | 1 | 1.726 | 0.0065 | 56 | 0.0024 | 8 | 0.0117 | 21 |
| w | 1 | 1 | 1.846 | 0.0069 | 57 | 0.00235 | 8 | 0.0118 | 21 |
| w | 1 | 2 | 1.726 | 0.0052 | 47 | 0.00188 | 7 | 0.0121 | 21 |
| w | 1 | 2 | 1.608 | 0.0073 | 52 | 0.00152 | 8 | 0.0124 | 21 |
| w | 1 | 2 | 1.844 | 0.0056 | 51 | 0.00154 | 7 | 0.0092 | 21 |
| w | 1 | 2 | 1.942 | 0.0057 | 55 | 0.0018 | 6 | 0.0092 | 21 |
| w | 1 | 2 | 1.836 | 0.0054 | 47 | 0.00166 | 8 | 0.0095 | 21 |
| w | 1 | 2 | 1.796 | 0.006 | 50 | 0.00167 | 8 | 0.0118 | 21 |
| w | 1 | 2 | 1.79 | 0.005 | 52 | 0.00076 | 6 | 0.0103 | 21 |
| w | 1 | 3 | 1.782 | 0.0067 | 60 | 0.00258 | 8 | 0.0115 | 21 |
| w | 1 | 3 | 1.826 | 0.0084 | 64 | 0.00165 | 8 | 0.013 | 21 |
| w | 1 | 3 | 1.642 | 0.0078 | 69 | 0.00157 | 8 | 0.0133 | 21 |

| | | | | | | | | | |
|---|---|---|---|---|---|---|---|---|---|
| w | 1 | 3 | 1.65 | 0.0072 | 57 | 0.00174 | 7 | 0.0132 | 21 |
| w | 1 | 3 | 1.728 | 0.0064 | 60 | 0.00113 | 5 | 0.013 | 21 |
| w | 1 | 3 | 1.722 | 0.0078 | 65 | 0.00162 | 5 | 0.0134 | 21 |
| w | 1 | 3 | 1.908 | 0.008 | 59 | 0.0013 | 5 | 0.0115 | 21 |
| w | 2 | 1 | 1.926 | 0.0097 | 66 | 0.00562 | 13 | 0.0177 | 11 |
| w | 2 | 1 | 1.864 | 0.0096 | 71 | 0.00415 | 13 | 0.016 | 11 |
| w | 2 | 1 | 1.952 | 0.0094 | 69 | 0.00482 | 14 | 0.0157 | 11 |
| w | 2 | 2 | 2.206 | 0.0103 | 76 | 0.0057 | 13 | 0.0145 | 11 |
| w | 2 | 2 | 1.772 | 0.0109 | 82 | 0.0044 | 13 | 0.0157 | 11 |
| w | 2 | 2 | 1.808 | 0.0104 | 87 | 0.004 | 10 | 0.0152 | 11 |
| w | 2 | 2 | 1.862 | 0.0106 | 84 | 0.00317 | 8 | 0.0141 | 11 |
| w | 2 | 2 | 1.844 | 0.011 | 85 | 0.00371 | 8 | 0.0157 | 11 |
| w | 2 | 3 | 1.8 | 0.0102 | 82 | 0.00446 | 8 | 0.0146 | 11 |
| w | 2 | 3 | 1.832 | 0.0116 | 88 | 0.00298 | 8 | 0.0155 | 11 |
| w | 2 | 3 | 1.97 | 0.0106 | 79 | 0.00315 | 8 | 0.0159 | 11 |
| w | 3 | 1 | 1.832 | 0.0082 | 57 | 0.00385 | 11 | 0.0146 | 17 |
| w | 3 | 1 | 1.692 | 0.0079 | 59 | 0.00394 | 13 | 0.0152 | 17 |
| w | 3 | 1 | 1.724 | 0.0085 | 59 | 0.00448 | 12 | 0.014 | 17 |
| w | 3 | 1 | 1.764 | 0.0088 | 69 | 0.00423 | 13 | 0.0142 | 17 |
| w | 3 | 1 | 1.89 | 0.0087 | 66 | 0.00358 | 11 | 0.0133 | 17 |
| w | 3 | 2 | 1.978 | 0.0075 | 65 | 0.00362 | 12 | 0.0138 | 17 |
| w | 3 | 2 | 1.836 | 0.0082 | 69 | 0.00358 | 10 | 0.014 | 17 |
| w | 3 | 2 | 1.872 | 0.0085 | 68 | 0.00358 | 8 | 0.0136 | 17 |
| w | 3 | 2 | 1.744 | 0.0083 | 74 | 0.00381 | 10 | 0.0121 | 17 |
| w | 3 | 2 | 2.04 | 0.0085 | 73 | 0.00325 | 8 | 0.0129 | 17 |
| w | 3 | 2 | 1.852 | 0.007 | 70 | 0.00279 | 8 | 0.0118 | 17 |
| w | 3 | 2 | 1.612 | 0.0086 | 73 | 0.00363 | 8 | 0.0131 | 17 |
| w | 3 | 3 | 1.664 | 0.008 | 68 | 0.0034 | 8 | 0.0141 | 17 |
| w | 3 | 3 | 1.944 | 0.0083 | 72 | 0.00347 | 8 | 0.015 | 17 |
| w | 3 | 3 | 1.83 | 0.008 | 68 | 0.00305 | 8 | 0.0141 | 17 |
| w | 3 | 3 | 1.836 | 0.0079 | 60 | 0.00298 | 8 | 0.0149 | 17 |
| w | 3 | 3 | 1.932 | 0.008 | 70 | 0.00376 | 8 | 0.014 | 17 |
| w | 5 | 1 | 1.492 | 0.0047 | 51 | 0.00184 | 8 | 0.0098 | 9 |
| w | 5 | 1 | 1.61 | 0.005 | 58 | 0.00234 | 8 | 0.009 | 9 |
| w | 5 | 1 | 1.514 | 0.0056 | 61 | 0.00263 | 6 | 0.0102 | 9 |
| w | 5 | 2 | 1.67 | 0.0059 | 59 | 0.00101 | 5 | 0.0097 | 9 |
| w | 5 | 2 | 1.724 | 0.0052 | 53 | 0.00137 | 5 | 0.0095 | 9 |
| w | 5 | 2 | 1.504 | 0.0056 | 62 | 0.00116 | 5 | 0.0089 | 9 |
| w | 5 | 3 | 1.672 | 0.0057 | 62 | 0.00131 | 5 | 0.0099 | 9 |
| w | 5 | 3 | 1.612 | 0.0051 | 62 | 0.00087 | 5 | 0.0094 | 9 |
| w | 5 | 3 | 1.572 | 0.005 | 52 | 0.00085 | 5 | 0.001 | 9 |
| x | 1 | 1 | 1.748 | 0.0095 | 62 | 0.00209 | 7 | 0.0128 | 9 |
| x | 1 | 1 | 1.522 | 0.00935 | 57 | 0.00228 | 9 | 0.01483 | 9 |

| | | | | | | | | | |
|---|---|---|---|---|---|---|---|---|---|
| x | 1 | 1 | 1.706 | 0.01075 | 66 | 0.00194 | 8 | 0.01535 | 9 |
| x | 1 | 2 | 1.688 | 0.00989 | 63 | 0.00202 | 8 | 0.01511 | 9 |
| x | 1 | 2 | 1.632 | 0.01002 | 68 | 0.00234 | 7 | 0.01334 | 9 |
| x | 1 | 2 | 1.788 | 0.00952 | 65 | 0.00211 | 8 | 0.01326 | 9 |
| x | 1 | 3 | 1.668 | 0.00939 | 71 | 0.00203 | 7 | 0.01218 | 9 |
| x | 1 | 3 | 1.548 | 0.00828 | 67 | 0.00259 | 5 | 0.01247 | 9 |
| x | 1 | 3 | 1.604 | 0.00755 | 66 | 0.00209 | 8 | 0.012 | 9 |
| x | 2 | 1 | 1.598 | 0.01162 | 80 | 0.00398 | 8 | 0.01523 | 12 |
| x | 2 | 1 | 1.704 | 0.00972 | 73 | 0.00636 | 12 | 0.01632 | 12 |
| x | 2 | 1 | 1.624 | 0.01103 | 82 | 0.00618 | 12 | 0.0158 | 12 |
| x | 2 | 1 | 1.732 | 0.01106 | 76 | 0.00566 | 11 | 0.01587 | 12 |
| x | 2 | 2 | 1.796 | 0.01154 | 83 | 0.00622 | 10 | 0.01613 | 12 |
| x | 2 | 2 | 1.542 | 0.01314 | 78 | 0.00381 | 8 | 0.01538 | 12 |
| x | 2 | 2 | 1.62 | 0.01274 | 83 | 0.0039 | 8 | 0.01661 | 12 |
| x | 2 | 2 | 1.608 | 0.01169 | 84 | 0.00516 | 8 | 0.01692 | 12 |
| x | 2 | 3 | 1.804 | 0.01139 | 81 | 0.00491 | 8 | 0.01546 | 12 |
| x | 2 | 3 | 1.708 | 0.01152 | 78 | 0.00333 | 8 | 0.01498 | 12 |
| x | 2 | 3 | 1.368 | 0.01167 | 80 | 0.00391 | 8 | 0.01459 | 12 |
| x | 2 | 3 | 1.69 | 0.01176 | 76 | 0.00402 | 8 | 0.01935 | 12 |
| x | 4 | 1 | 1.746 | 0.00677 | 44 | 0.00356 | 8 | 0.01498 | 10 |
| x | 4 | 1 | 1.812 | 0.00716 | 52 | 0.00358 | 8 | 0.01534 | 10 |
| x | 4 | 1 | 1.748 | 0.00707 | 52 | 0.00306 | 8 | 0.01545 | 10 |
| x | 4 | 2 | 1.834 | 0.00797 | 55 | 0.00307 | 8 | 0.01582 | 10 |
| x | 4 | 2 | 1.836 | 0.00789 | 52 | 0.00251 | 8 | 0.01681 | 10 |
| x | 4 | 2 | 1.91 | 0.00806 | 53 | 0.00271 | 8 | 0.01605 | 10 |
| x | 4 | 2 | 1.8 | 0.00837 | 57 | 0.00339 | 8 | 0.01576 | 10 |
| x | 4 | 3 | 1.938 | 0.00839 | 54 | 0.00284 | 8 | 0.01623 | 10 |
| x | 4 | 3 | 1.966 | 0.00892 | 59 | 0.00256 | 8 | 0.01678 | 10 |
| x | 4 | 3 | 1.768 | 0.00893 | 57 | 0.0028 | 7 | 0.01356 | 10 |
| x | 5 | 1 | 1.564 | 0.00835 | 74 | 0.00301 | 9 | 0.01119 | 23 |
| x | 5 | 1 | 1.568 | 0.00892 | 73 | 0.00291 | 7 | 0.01232 | 23 |
| x | 5 | 1 | 1.622 | 0.00603 | 58 | 0.00558 | 13 | 0.01016 | 23 |
| x | 5 | 1 | 1.776 | 0.00794 | 77 | 0.00391 | 12 | 0.01295 | 23 |
| x | 5 | 1 | 1.746 | 0.00858 | 68 | 0.00199 | 8 | 0.01243 | 23 |
| x | 5 | 1 | 1.708 | 0.00862 | 76 | 0.00271 | 8 | 0.01193 | 23 |
| x | 5 | 1 | 1.692 | 0.00843 | 70 | 0.00314 | 8 | 0.01149 | 23 |
| x | 5 | 2 | 1.734 | 0.00962 | 75 | 0.00282 | 8 | 0.01275 | 23 |
| x | 5 | 2 | 1.79 | 0.00936 | 78 | 0.00251 | 8 | 0.01248 | 23 |
| x | 5 | 2 | 1.578 | 0.00769 | 56 | 0.00512 | 12 | 0.01509 | 23 |
| x | 5 | 2 | 1.454 | 0.00834 | 56 | 0.00418 | 11 | 0.01439 | 23 |
| x | 5 | 2 | 1.58 | 0.00833 | 56 | 0.00568 | 13 | 0.01361 | 23 |
| x | 5 | 2 | 1.418 | 0.00807 | 52 | 0.00307 | 12 | 0.01454 | 23 |
| x | 5 | 2 | 1.734 | 0.00781 | 80 | 0.00242 | 8 | 0.01167 | 23 |

| | | | | | | | | | |
|---|---|---|---|---|---|---|---|---|---|
| x | 5 | 2 | 1.52 | 0.0075 | 70 | 0.0027 | 8 | 0.01161 | 23 |
| x | 5 | 2 | 1.584 | 0.00659 | 68 | 0.00179 | 7 | 0.00875 | 23 |
| x | 5 | 3 | 1.728 | 0.00681 | 67 | 0.00121 | 6 | 0.00954 | 23 |
| x | 5 | 3 | 1.672 | 0.00754 | 62 | 0.00107 | 6 | 0.00952 | 23 |
| x | 5 | 3 | 1.404 | 0.00575 | 66 | 0.00073 | 5 | 0.00953 | 23 |
| x | 5 | 3 | 1.584 | 0.00617 | 61 | 0.00089 | 5 | 0.0093 | 23 |
| x | 5 | 3 | 1.474 | 0.00678 | 67 | 0.00099 | 5 | 0.0088 | 23 |
| x | 5 | 3 | 1.402 | 0.00611 | 66 | 0.00075 | 5 | 0.00943 | 23 |
| x | 5 | 3 | 1.586 | 0.00629 | 72 | 0.00084 | 5 | 0.00977 | 23 |
| y | 1 | 1 | 1.77 | 0.00969 | 73 | 0.005 | 13 | 0.01602 | 13 |
| y | 1 | 1 | 1.692 | 0.00949 | 71 | 0.00399 | 12 | 0.01586 | 13 |
| y | 1 | 1 | 1.546 | 0.01004 | 73 | 0.00403 | 12 | 0.01752 | 13 |
| y | 1 | 1 | 1.956 | 0.00696 | 67 | 0.0046 | 11 | 0.01386 | 13 |
| y | 1 | 2 | 1.564 | 0.00922 | 71 | 0.00469 | 13 | 0.01602 | 13 |
| y | 1 | 2 | 1.738 | 0.00972 | 80 | 0.00326 | 12 | 0.01502 | 13 |
| y | 1 | 2 | 1.836 | 0.00885 | 73 | 0.00421 | 11 | 0.01613 | 13 |
| y | 1 | 2 | 1.744 | 0.00874 | 75 | 0.00512 | 11 | 0.01366 | 13 |
| y | 1 | 2 | 1.738 | 0.00788 | 77 | 0.00417 | 11 | 0.01448 | 13 |
| y | 1 | 3 | 1.478 | 0.01 | 80 | 0.00259 | 9 | 0.01471 | 13 |
| y | 1 | 3 | 1.62 | 0.00801 | 67 | 0.00378 | 8 | 0.01375 | 13 |
| y | 1 | 3 | 1.838 | 0.0074 | 69 | 0.00398 | 8 | 0.01454 | 13 |
| y | 1 | 3 | 1.524 | 0.00701 | 64 | 0.00205 | 6 | 0.01312 | 13 |
| y | 4 | 1 | 1.582 | 0.008 | 59 | 0.00692 | 13 | 0.0173 | 9 |
| y | 4 | 1 | 1.546 | 0.0074 | 56 | 0.00604 | 15 | 0.0158 | 9 |
| y | 4 | 1 | 1.834 | 0.0089 | 72 | 0.00614 | 12 | 0.0152 | 9 |
| y | 4 | 2 | 1.756 | 0.0071 | 58 | 0.00426 | 10 | 0.0143 | 9 |
| y | 4 | 2 | 1.698 | 0.0101 | 77 | 0.00606 | 11 | 0.016 | 9 |
| y | 4 | 2 | 1.574 | 0.0091 | 74 | 0.00388 | 10 | 0.015 | 9 |
| y | 4 | 3 | 1.762 | 0.00543 | 56 | 0.00196 | 5 | 0.0174 | 9 |
| y | 4 | 3 | 1.612 | 0.0092 | 73 | 0.00262 | 8 | 0.015 | 9 |
| y | 4 | 3 | 1.92 | 0.0037 | 38 | 0.00158 | 3 | 0.0097 | 9 |
| y | 3 | 1 | 1.734 | 0.0067 | 59 | 0.00719 | 17 | 0.0145 | 10 |
| y | 3 | 1 | 1.79 | 0.0069 | 61 | 0.00527 | 15 | 0.0141 | 10 |
| y | 3 | 1 | 1.75 | 0.0075 | 62 | 0.00512 | 16 | 0.0146 | 10 |
| y | 3 | 2 | 1.738 | 0.0068 | 56 | 0.00567 | 16 | 0.0129 | 10 |
| y | 3 | 2 | 1.884 | 0.0067 | 69 | 0.00576 | 14 | 0.0129 | 10 |
| y | 3 | 2 | 1.81 | 0.0069 | 67 | 0.00671 | 15 | 0.012 | 10 |
| y | 3 | 2 | 1.718 | 0.0073 | 68 | 0.00533 | 12 | 0.0115 | 10 |
| y | 3 | 3 | 1.648 | 0.0072 | 65 | 0.00529 | 13 | 0.012 | 10 |
| y | 3 | 3 | 1.442 | 0.0043 | 38 | 0.00249 | 7 | 0.0125 | 10 |
| y | 3 | 3 | 1.644 | 0.005 | 54 | 0.00318 | 9 | 0.0123 | 10 |
| y | 5 | 1 | 1.78 | 0.0101 | 65 | 0.0063 | 13 | 0.0189 | 16 |
| y | 5 | 1 | 1.73 | 0.0105 | 69 | 0.0071 | 14 | 0.0185 | 16 |

| | | | | | | | | | |
|---|---|---|---|---|---|---|---|---|---|
| y | 5 | 1 | 1.86 | 0.01 | 63 | 0.0059 | 13 | 0.0195 | 16 |
| y | 5 | 1 | 1.692 | 0.0105 | 63 | 0.0046 | 12 | 0.019 | 16 |
| y | 5 | 1 | 1.754 | 0.0093 | 60 | 0.0068 | 13 | 0.0169 | 16 |
| y | 5 | 2 | 1.636 | 0.0092 | 67 | 0.006 | 13 | 0.0145 | 16 |
| y | 5 | 2 | 1.772 | 0.0095 | 68 | 0.0069 | 13 | 0.0147 | 16 |
| y | 5 | 2 | 1.75 | 0.001 | 69 | 0.0055 | 12 | 0.0156 | 16 |
| y | 5 | 2 | 1.702 | 0.0092 | 75 | 0.0036 | 12 | 0.0146 | 16 |
| y | 5 | 2 | 1.616 | 0.0096 | 77 | 0.0051 | 12 | 0.0151 | 16 |
| y | 5 | 2 | 1.646 | 0.0104 | 72 | 0.0057 | 12 | 0.0168 | 16 |
| y | 5 | 3 | 1.732 | 0.008 | 65 | 0.0046 | 11 | 0.0137 | 16 |
| y | 5 | 3 | 1.756 | 0.009 | 73 | 0.0055 | 13 | 0.0141 | 16 |
| y | 5 | 3 | 1.52 | 0.0081 | 69 | 0.0047 | 12 | 0.0149 | 16 |
| y | 5 | 3 | 1.58 | 0.0091 | 71 | 0.0038 | 12 | 0.0145 | 16 |
| y | 5 | 3 | 1.734 | 0.0035 | 27 | 0.0031 | 5 | 0.0147 | 16 |
